# Targeted Chemical Modifications Identify Key Features of Carbohydrate Assemblies and Generate Tailored Carbohydrate Materials

**DOI:** 10.1002/chem.202102164

**Published:** 2021-08-04

**Authors:** Soeun Gim, Giulio Fittolani, Yang Yu, Yuntao Zhu, Peter H. Seeberger, Yu Ogawa, Martina Delbianco

**Affiliations:** ^1^ Department of Biomolecular Systems Max-Planck-Institute of Colloids and Interfaces Am Mühlenberg 1 14476 Potsdam Germany; ^2^ Department of Chemistry and Biochemistry Freie Universität Berlin Arnimallee 22 14195 Berlin Germany; ^3^ Simpson Querrey Institute Northwestern University 2145 Sheridan Road Evanston IL 60208 USA; ^4^ Univ. Grenoble Alpes CNRS, CERMAV 38000 Grenoble France

**Keywords:** carbohydrates, electron diffraction, electron tomography, site-specific modifications, supramolecular assemblies

## Abstract

The molecular level description of carbohydrate assemblies is hampered by their structural complexity and the lack of suitable analytical methods. Here, we employed systematic chemical modifications to identify key non‐covalent interactions that triggered the supramolecular assembly of a disaccharide model. While some modifications disrupted the supramolecular organization, others were tolerated, delivering important information on the aggregation process. The screening identified new geometries, including nanotubes, and twisted ribbons that were characterized with electron tomography and electron diffraction (ED) methods. This work demonstrates that the combination of synthetic chemistry and ED methods is a powerful tool to draw correlations between the molecular structure and the nanoscale architecture of carbohydrate assemblies.

## Graphical abstract

**Site‐specific modifications** identified key non‐covalent interactions responsible for the supramolecular assembly of a disaccharide model. Electron diffraction (ED) and tomography enabled the correlation between molecular structure and nanoscale architecture, confirming that C−H⋅⋅⋅π edge‐to‐face type interactions between the aromatic rings and water‐bridged hydrogen bonds between specific hydroxyl groups stabilized the assembly. The synthetic compounds were used to generate carbohydrate‐based nanotubes, twisted ribbons, as well as spherical particles that were characterized with molecular level resolution.

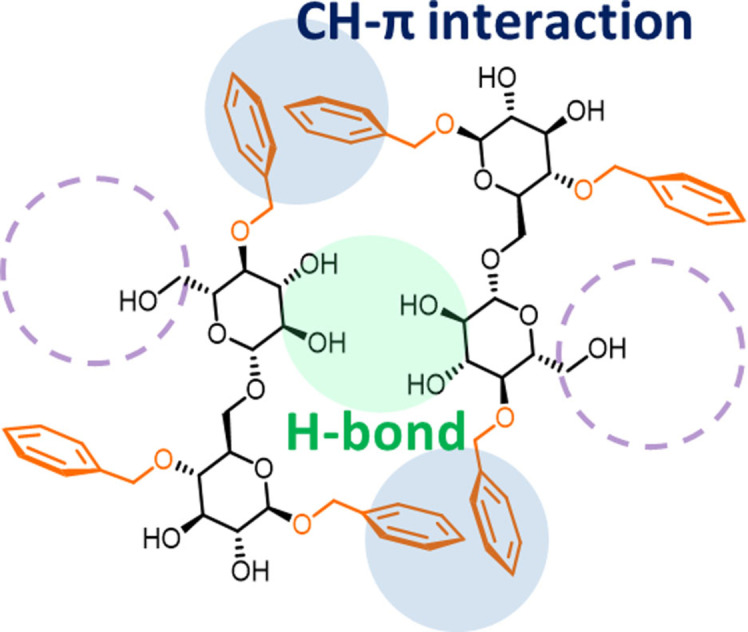

Self‐assembly is a key process to build functional structures in nature.^[1]^ Assemblies of biomolecules, such as peptides^[2]^ and nucleic acids^[3]^, have been extensively studied, often relying on well‐defined synthetic analogues. These compounds simplified the analysis and identified the key interactions responsible for the assembly of the natural counterpart.^[4]^ Moreover, synthetic systems enabled fine‐tuning of functional groups to achieve functions on demand.^[5]^ Despite the tendency of polysaccharides to form materials with outstanding mechanical and photophysical properties,^[6]^ only few examples of synthetic carbohydrate‐based assemblies were reported.^[7]^ The hydrophilic nature of simple monosaccharides has been mainly exploited to decorate supramolecular systems^[8]^ or to generate glycoamphiphiles forming low molecular weight gelators.^[9]^ Still, the structural role of the carbohydrate part remained mostly overlooked.

Carbohydrates could guide and stabilize the formation of supramolecular structures due to their ability to form non‐covalent directional interactions.^[10]^ Still, understanding their mode of aggregation and types of intermolecular interactions remains a challenge. Lack of broad libraries of well‐defined compounds and suitable analytical techniques have prevented extended systematic studies.^[11]^ Model systems are essential to capture molecular details responsible for the formation of carbohydrate materials and to develop reliable analytical methods.^[12]^ Systematic site‐specific modifications could prompt the identification of key features of the supramolecular organization, such as particular H‐bonds^[13]^ and carbohydrate‐aromatic interactions,^[14]^ and could generate novel geometries.

Disaccharide **1** self‐assembles into highly crystalline twisted fibers (Figure 1A) and is the ideal standard to implement microcrystal electron diffraction (microED) to study carbohydrate materials.^[7b]^ A microED‐based molecular packing model (Figure 1A) identified key intermolecular interactions responsible for the formation of the supramolecular fiber: C−H⋅⋅⋅π edge‐to‐face type interactions between the aromatic rings and water‐bridged hydrogen bonds between specific hydroxyl groups stabilized the assembly.^[12b]^

**Figure 1:**
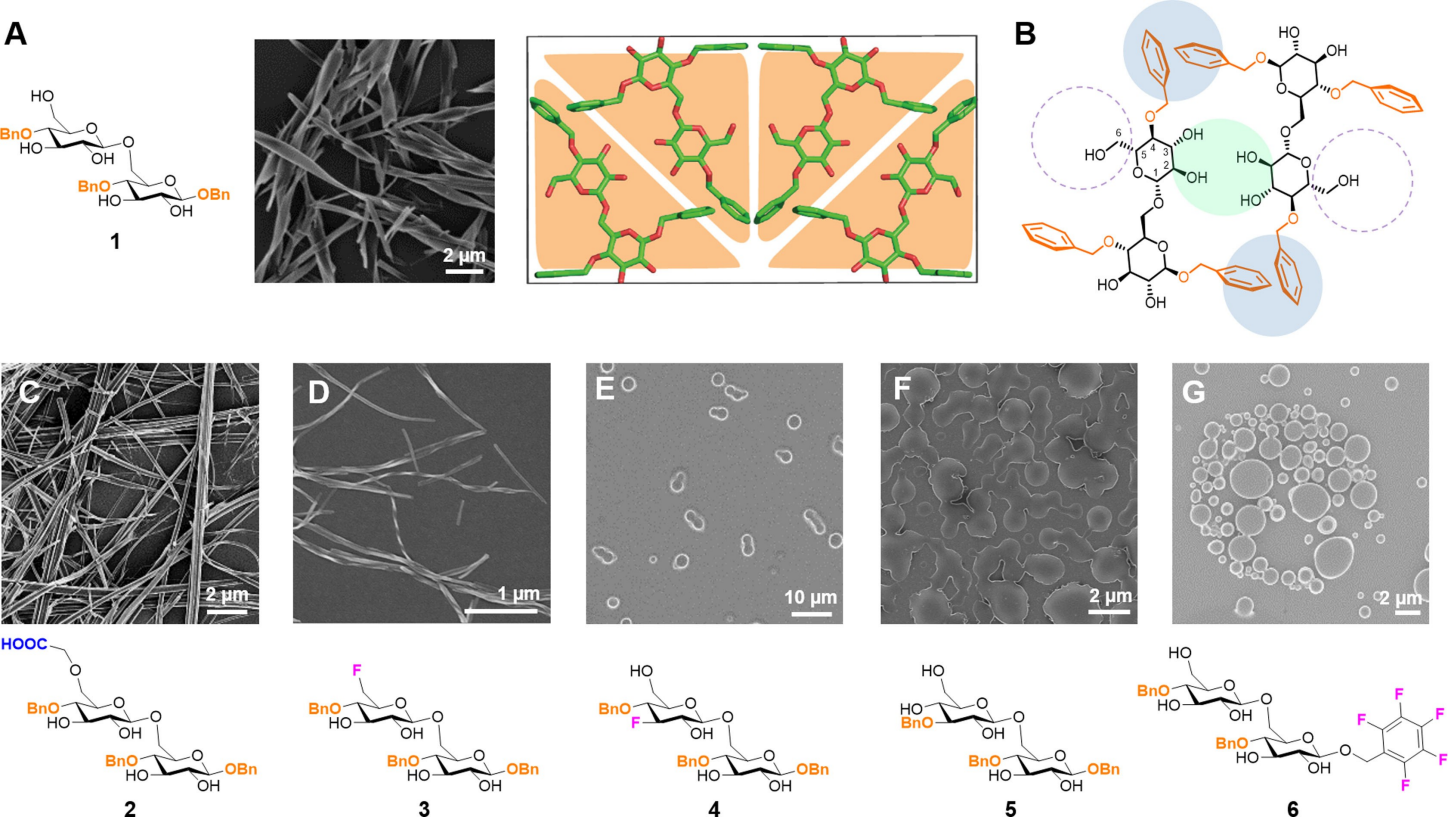
Chemical structure of **1**, SEM image of its assembly and tentative molecular packing model of **1** in the unit cell (A).^[12b]^ Schematic representation of the unit cell to highlight the site of modifications (B). Chemical structures and SEM images of their assemblies for **2** (C), **3** (D), **4** (E), **5** (F), and **6** (G).

In order to validate the proposed crystal organization and explore flexibility and tuneability of the system, we designed seven analogues of compound **1** (Figure 1C−G and S1, compounds **2**–**8**). Each modification was aimed to disrupt a particular interaction and highlight the importance of the replaced moiety in the assembly process. Compounds **2**, **3**, and **4** possess the same benzyl (Bn) group pattern as **1** and are modified at the hydroxyl groups of the glucose unit at the non‐reducing end. In compound **2**, the hydroxyl group at the C‐6 position is alkylated with a carboxymethyl group. This moiety introduces a negative charge in the structure and could be exploited for conjugation. Additionally, the carboxylic acid should be still available for H‐bonding, albeit with an increased steric bulk.^[15]^ Compounds **3** and **4** are the C‐6 and C‐3 deoxyfluorinated analogues, respectively. Deoxyfluorination was designed to selectively disrupt specific H‐bonds.^[16]^ Additionally, the substitution of a hydroxyl group with a fluorine atom should increase lipophilicity and affect the dipole of the molecule.^[17]^ Compound **5** has a different Bn pattern to explore how the position of the Bn groups affect the supramolecular assembly. Compound **6** bears a pentafluorobenzyl (PFB) group at the anomeric position, resulting in enhanced hydrophobicity and altered electrostatic interactions. The PFB group does not engage in C−H⋅⋅⋅π edge‐to‐face stacking because the electron rich *para*‐F does not interact with electron rich phenyl rings.^[18]^ Compounds **7** and **8** (Figure S2) are more heavily modified to explore the flexibility of the system.

Upon solvent switch (HFIP to water, see Supporting Information), **2** and **3** formed insoluble fibrous structures (Figure 1C and D), whereas **4**–**8** remained soluble (Figure 1E–G), suggesting that the assembly process tolerates modifications at the C‐6 position at the non‐reducing end (Figure 1B, highlighted with dotted circles). Compound **2** developed into longer and thicker fibers without visible helicity when compared to the assembly of **1**. Fibrils obtained from the C‐6 deoxyfluorinated analogue **3** were shorter, thinner, and twisted (Figure 1D and S6). The assembly outcome was drastically different for the monofluorinated compounds **3** and **4** (Figure 1D and E), demonstrating the importance of the C‐3 hydroxyl group for the supramolecular assembly (Figure 1B, highlighted with a green circle). For the latter, colloidal particles rather than fibers were observed with cryogenic SEM measurement (Figure S11A). Alteration of the Bn pattern (compound **5**) prevented the formation of any defined structure (Figure 1F). Thus, the Bn pattern in **1** is essential to arrange the aromatic groups in a particular spatial orientation while maintaining the hydroxyl group at C‐3 available for H‐bonds, promoting self‐assembly into fibers. The lack of supramolecular aggregation observed for **6** (Figure 1G) supports the presence of C−H⋅⋅⋅π type edge‐to‐face interactions in the fiber obtained from **1** (Figure 1B, highlighted with blue circles).

This first screening identified **2** and **3** as the only compounds able to maintain the fibrous structure. Thus, we focused on their self‐assembly processes and crystalline structures. We previously captured the real‐time assembly of **1**, showing that the fibers generated from highly concentrated HFIP droplets containing the oligosaccharide, upon addition of water (Figure S3A).^[7b]^ The same phenomenon was observed for **3** (Figure S3B), whereas precipitation of **2** followed slower kinetics and could not be monitored in real time. Crystallization of **2** was followed with polarized optical microscopy on a surface, revealing that the formation of highly organized crystalline fibers, with length of hundreds of μm, was completed after a week (Figure S4). The powder XRD and microED patterns of **3** were similar to **1** (Figure S5). Still, the fibrils from **3** retained a higher level of flexibility (i. e. twist and curvature), likely arising from displacement between stacked molecules along the fiber axis.

The assembly of **2** was tested at different pH. Fiber‐like structures were obtained regardless of the pH; yet, showing a faster growth rate and a larger amount of precipitation at lower pH (Figure S7). TEM observation revealed that different pH conditions resulted in distinctive internal structures of the fibers: tube‐like fibers were dominant at pH 3 and pH 7, while single‐crystal fibers similar to that of compound **1** were prevalent at pH 11. In the latter, thick crystalline fibers with a diameter larger than 500 nm were observed along with thin and flexible fibrils (Figure S7E and S7F), likely resulting from the slower crystal growth in basic condition. Electron tomography demonstrated that the tubes obtained at pH 3 and pH 7 are composed of multiple fibrous crystallites with a hollow core, sometimes connected with thin lamella‐like crystals (Figure 2 and S8). The structure differs from a typical tube structure with a well‐defined hole, often found in inorganic systems,^[19]^ but adopts a dome‐like arrangement. The component crystallites, with a width of approximately 100 nm, tightly assemble into a larger fiber with several internal tunnel‐like holes (Figure 2A, green arrows). The component crystallites align almost perfectly parallel along the fiber axis, while they do not merge with each other to form a bigger single crystallite. In these tube structures, the crystal growth of individual crystallites is restricted in the lateral directions compared to the case of single‐crystal fibers, resulting in holes in the middle of the structure.

**Figure 2:**
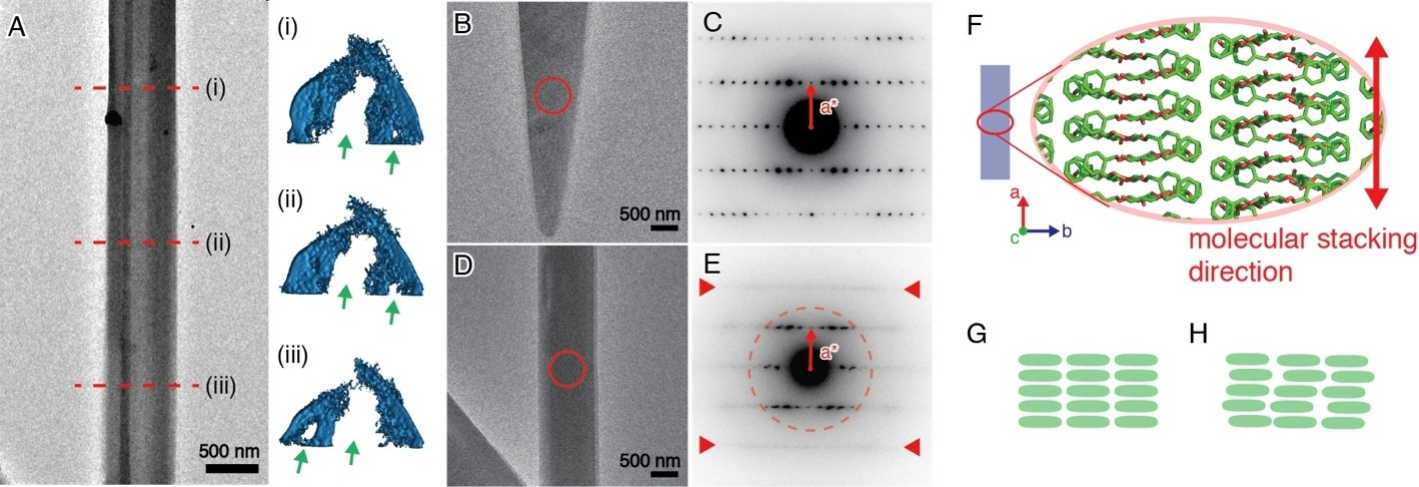
TEM image of the assembly of **2** at pH 3 with electron tomographic cross sections at different positions along its fiber axis (A). Green arrows indicate internal holes in the assembly. TEM images of the assembly of **2** at pH 3 (B, D) and electron diffraction patterns obtained from the circled areas in panel B (C) and in panel D (E), respectively. In panel E, spot‐like reflections are observed only within the circled area up to the resolution of only about 3 Å. Red arrow heads indicate the streak lines. Schematic illustration of the molecular packing manner in the fibrous assembly, adapted from the packing model of the assembly of **1** (F).^[12b]^ Schematic illustrations of molecular packing of **2** with (G) and without (H) long‐range structural order in the lateral plane of the fibrous assembly.

Some single‐crystal fibers provided high‐resolution ED patterns (Figure 2C, the resolution can reach higher than 1 Å), indicating the crystalline organization of the molecules in the fibers (Figure 2G). The diffraction pattern was essentially identical to that from the assembly of **1**. Thus, **1** and **2** share similar unit cell dimensions and molecular packing. Many fibers, especially those with tube‐like morphology, are polycrystalline, as ED patterns from such fibers contained more than one crystallographic projection (Figure 2E). This indicates that the component crystallites have different crystallographic orientations in the lateral plane of the fiber. Spot‐like reflections appeared only in the inner part of the diffraction diagram (Figure 2E, red dashed circled area) and rapidly disappeared upon electron radiation. The loss of spots in the higher angle part indicates the lack of strict long‐range structural order. In the outer side, streaks were visible along the direction transverse to the a* vector. Considering the molecular packing model (Figure 2F) where the *a*‐axis (fiber axis) corresponds to the molecular stacking direction, the presence of the streak implies that the periodicity along the stacking direction is preserved. On the other hand, disorder should be present in the molecular organization in the lateral plane of the fiber (Figure 2H). This observation supports the hypothesis that molecular packing in the lateral plane is stabilized by the C‐6 hydroxyl group involved in H‐bonds. The disorder might be induced by steric hindrance resulting from the bulky carboxymethyl group or by additional water molecules solvating the carboxylic moiety.

To further explore the flexibility of this system, we performed a co‐assembly between **1** and other modified analogues (1 : 1 ratio by mass).^[20]^ While most of the compounds were self‐sorted or resulted in random aggregation (Figure S10), the co‐assembly with C‐6 substituted samples **2** and **3** resulted in fiber formation. The assembly proceeded with slower aggregation rates (one day for completion), forming left‐handed chiral fibers with enhanced helicity (Figure 3A and B and S10C). Twisted ribbon‐like structures were obtained from the co‐assembly of **1** and **2** (Figure 3A); these aggregates had similar dimensions to the flat fibers obtained from **2** alone, but increased helicity. The co‐assembly of **1** and **3** generated long and highly twisted left‐handed fibers with a regular pitch of 1–2 μm (Figure 3B). Since the supramolecular helicity originates from a slight rotation between the stacked molecules along the fiber direction,^[12b]^ we speculate that co‐assembly generates “defects” during crystallization, giving rise to an increased twist. The decreased rate of crystallization may also play an important role in the modulation of the twist.^[21]^

**Figure 3:**
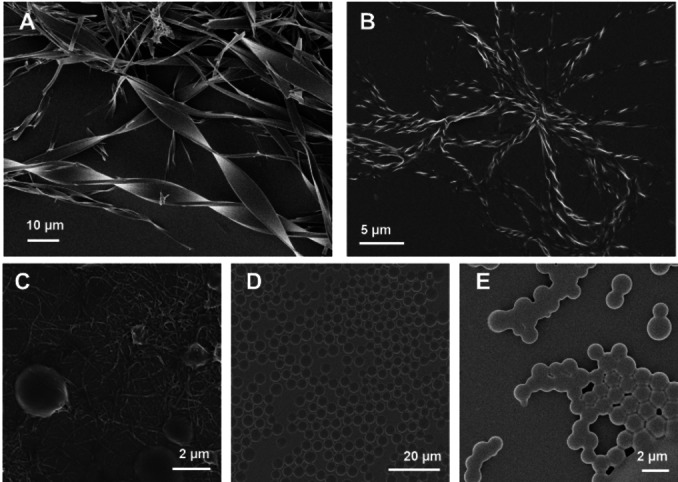
SEM images of the co‐assembly between **1** and **2** (A), **3** (B) in 1:1 mass ratios. SEM images of the co‐assembly between **1** and **4** with the mass ratios of 1:1 (C), 1:2 (D), and 1:4 (E).

Surprisingly, no precipitate was observed upon co‐assembly of **1** and **4**, resulting in an interconnected colloidal network composed of spherical particles and fibers (Figure 3C, S11B, and S12A). This result prompted us to screen different mass ratios, revealing that, in the presence of higher amounts of **4**, colloidal particles could be generated. The colloidal solutions were stable for over six months. The 1 : 2 mass ratio generated homogeneous spherical particles that retained the spherical shape even upon drying (Figure 3D, S11C, and S12B). In contrast, higher amounts of **4** decreased the particle stability that merged upon drying (Figure 3E, S11D, and S12C).

In conclusion, we prepared a collection of disaccharides as models to understand carbohydrate assembly and to generate tailored supramolecular architectures. Targeted chemical modifications permitted to identify key features responsible for the formation of supramolecular fibers. Substitutions at the hydroxyl group at C‐6 position were tolerated by the assembly process, offering an opportunity for further functionalization. In contrast, modifications at the aromatic groups or at the hydroxyl group at the C‐3 position disrupted the assembly. Co‐assembly experiments supported the hypothesis that the introduction of defects during crystallization could modulate the fiber helicity and pitch, suggesting a mode to design tunable chiral structures. Site‐specific modifications of **1** generated novel geometries including hollow tubular structures, spherical colloidal particles, as well as highly twisted fibers/ribbons. Electron tomography and electron diffraction enabled the characterization of the self‐assembled structures with molecular level resolution. While these techniques are relatively underused for structural characterization of carbohydrates, apart from some examples in nanocellulose science^[22]^, we demonstrated that ED‐methods are powerful tools to investigate the correlation between molecular structure and nanoscale architecture of carbohydrates. The high‐resolution view of the self‐assembled structures offered by ED is hardly achievable with other methods.

## Conflict of interest

The authors declare no conflict of interest.

## Supporting information

As a service to our authors and readers, this journal provides supporting information supplied by the authors. Such materials are peer reviewed and may be re‐organized for online delivery, but are not copy‐edited or typeset. Technical support issues arising from supporting information (other than missing files) should be addressed to the authors.

Supporting Information
