# Supplementary material for: Targeted Chemical Modifications Identify Key Features of Carbohydrate Assemblies and Generate Tailored Carbohydrate Materials
Source: Chemistry. 2021 Aug 4;27(52):13139–43. doi: 10.1002/chem.202102164 (PMC8518775; doi:10.1002/chem.202102164)
Supplement: Supplementary file 1 — Supporting Information [file CHEM-27-13139-s001.pdf]

Chemistry–A European Journal

Supporting Information

Targeted Chemical Modifications Identify Key Features of Carbohydrate Assemblies and Generate Tailored Carbohydrate Materials

Soeun Gim, Giulio Fittolani, Yang Yu, Yuntao Zhu, Peter H. Seeberger, Yu Ogawa, and Martina Delbianco*

Supporting information

Table of contents

1. General materials and methods 2
2. Synthesis 3
   2.1. Synthesis of 2 3
   2.2. Synthesis of 3 7
   2.3. Synthesis of 4 12
   2.4. Synthesis of 5 22
   2.5. Synthesis of 6 30
   2.6. Synthesis of 7 39
   2.7. Synthesis of 8 43
3. Assembly of carbohydrates 46
4. References 50

1. General materials and methods

All chemicals used were reagent grade and used as supplied unless otherwise noted. Analytical thin-layer chromatography (TLC) was performed on Merck silica gel 60 F254 plates (0.25 mm). Compounds were visualized by UV irradiation or dipping the plate in a staining solution (sugar stain: 10% H_2SO_4 in EtOH; CAM: 48 g/L ammonium molybdate, 60 g/L ceric ammonium molybdate in 6% H_2SO_4 aqueous solution). Flash column chromatography was carried out by using forced flow of the indicated solvent on Fluka Kieselgel 60 M (0.04 – 0.063 mm). Analysis and purification by normal and reverse phase HPLC was performed by using an Agilent 1200 series. Products were lyophilized using a Christ Alpha 2-4 LD plus freeze dryer. ^{1}H, ^{13}C, HSQC, and COSY NMR spectra were recorded on a Varian 400-MR (400 MHz), Varian 600-MR (600 MHz), or Bruker Biospin AVANCE700 (700 MHz) spectrometer. Spectra were recorded in $CDCl_3$ by using the solvent residual peak chemical shift as the internal standard ($CDCl_3$: 7.26 ppm ^{1}H, 77.0 ppm ^{13}C), in D_2O using the solvent as the internal standard (D_2O: 4.79 ppm ^{1}H) or in MeOD using the solvent as the internal standard (MeOD: 4.87 ppm ^{1}H, 49.0 ppm ^{13}C). High resolution mass spectra were obtained using a 6210 ESI-TOF mass spectrometer (Agilent) and a MALDI-TOF autoflexTM (Bruker). MALDI and ESI mass spectra were run on IonSpec Ultima instruments. IR spectra were recorded on a Perkin-Elmer 1600 FTIR spectrometer. Optical rotations were measured by using a Perkin-Elmer 241 and Unipol L1000 polarimeter.

Scanning electron microscopy (SEM) images were obtained with a Gemini SEM, LEO 1550 system with cold field emission gun operation at 3 kV. All the samples were coated with Au/Pd. Transmission electron microscopy (TEM) images were obtained on carbon-coated copper grids with a Zeiss EM 912Ω instrument at 120 kV. Electron diffraction (ED) and electron tomography experiments were performed on a JEM 2100Plus transmission electron microscope (Jeol, Japan) operated at 200 kV. Selected area ED patterns were recorded at cryogenic temperature using an Elsa cryo holder (Gatan, the U.S.A). The experimental procedure of ED has been described elsewhere.[1] Tilt series images were acquired using SerialEM software[2] between tilt angles of ±65° with an angle increment of 2°. To avoid the diffraction contrast effect, the crystals were first irradiated with electron beam of 4 $e^{-}/Å^{-1}$ for 8 s. Thanks to their electron sensitivity, the crystals lost the diffraction contrast after this initial exposure. The image alignments and 3D reconstructions were done using IMOD software suit. The tomograms were visualized using 3dmod. The Cryogenic scanning electron microscopy (cryo-SEM) images were obtained from JEOL JSM 7500 F with frozen and Pt-coated droplet samples. Fiber formation in liquid was observed with a LEICA DMi8 Confocal Laser Microscope (20X dry objective). Fluorescence images were acquired with an excitation wavelength at 405 nm. Polarized optical microscopy images were obtained with an Olympus BX41 (40X) system. XRD profiles were recorded with an X-ray diffractometer Bruker D8 with Cu Kα radiation.

2. Synthesis

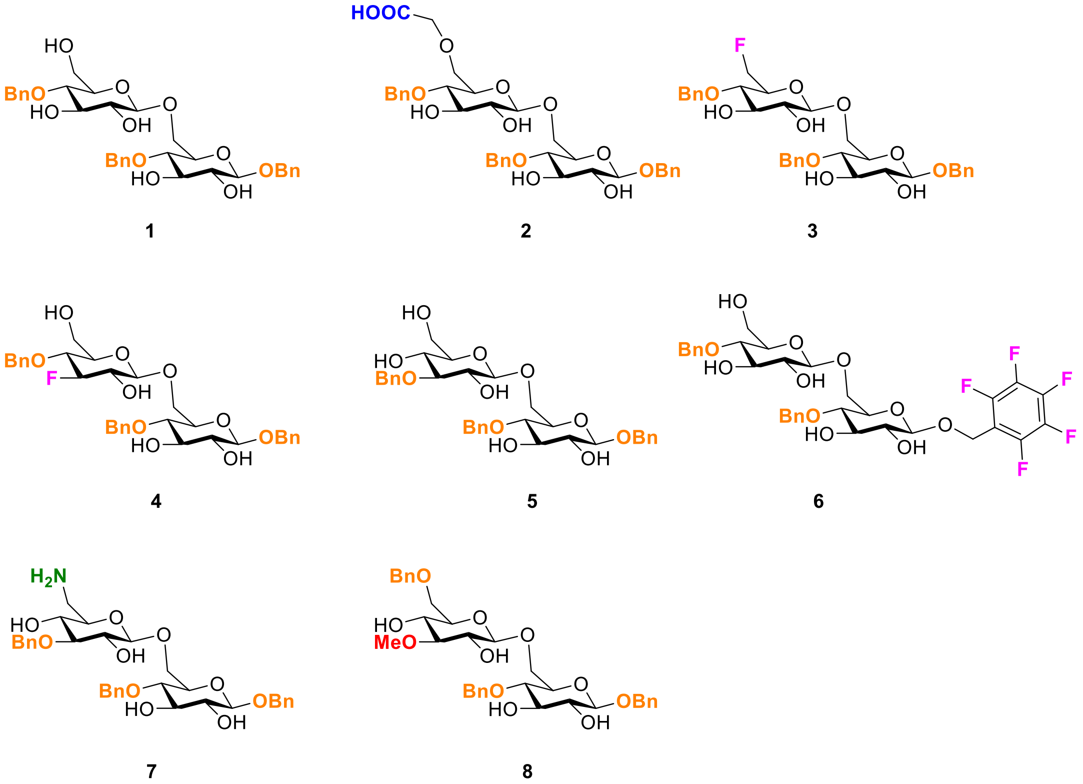

Figure S1 Chemical structure of the disaccharides used in this work. Compound **1** was synthesized according to a previously reported procedure.[3]

2.1. Synthesis of 2

*Synthesis of **S1***

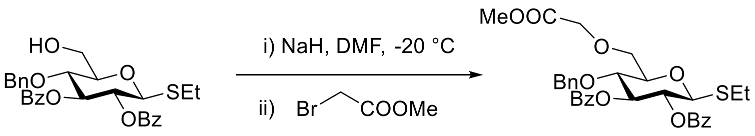

S0 **S1**

S0 was prepared according to previously established procedures.[4]

Ethyl 2,3-*O*-benzoyl-4-*O*-benzyl-1-thio-β-D-glucopyranoside, **S0** (50 mg, 0.10 mmol), previously coevaporated with toluene (3 x 10 mL), was dissolved in DMF (5 mL) at low temperature under N_2 atmosphere. Multiple additions of NaH (3 x 9.8 mg, 3 x 2.5 equiv, 60% in mineral oil) and methyl-bromoaceate (3 x 29 µL, 3 x 3 equiv) were performed every 4 h at -20 °C. The reaction was then quenched with acetic acid (1 mL). The mixture was diluted with DCM and washed with sat. aq. $NaHCO_3$. The organic layer was dried over anhydrous Na_2SO_4, filtered and concentrated *in vacuo*. The crude product was purified by column chromatography (Hexane:EtOAc = 5:1→2:1) to give **S1** as yellow oil (17 mg, 30 %). ^{1}H NMR (400 MHz, $CDCl_3$) δ 7.92 (ddd, *J* = 8.8, 7.6, 1.4 Hz, 4H), 7.48 (dddd, *J* = 11.3, 6.2, 3.2, 1.9 Hz, 2H), 7.39 – 7.32 (m, 4H), 7.15 (s, 5H), 5.73 (t, *J* = 9.4 Hz, 1H), 5.39 (t, *J* = 9.8 Hz, 1H), 4.73 – 4.65 (m, 2H), 4.61 (d, *J* = 10.9 Hz, 1H), 4.32 – 4.17 (m, 2H), 4.06 – 4.00 (m, 1H), 4.00 – 3.96 (m, 1H), 3.85 (dd, *J* = 11.4, 1.9 Hz,

1H), 3.77 (s, 3H), 3.70 (ddt, J = 9.8, 3.9, 2.1 Hz, 1H), 2.80 – 2.66 (m, 2H), 1.25 (t, J = 7.4 Hz, 3H); ^{13}C NMR (101 MHz, $CDCl_3$) δ 170.88, 170.19, 165.49, 137.46, 133.26, 129.82, 129.49, 128.40, 127.93, 83.73, 79.56, 77.36, 76.39, 75.53, 74.80, 70.81, 70.21, 69.28, 68.18, 52.71, 52.13, 51.96, 32.04, 29.81, 24.34, 22.81, 14.96, 14.26; $[\alpha]_D^{25}$ 32.9 (c = 1, $CHCl_3$); IR (neat) v_{max} = 3022, 1731, 1215 cm^{-1}; (ESI-HRMS) m/z 617.1821 $[M + Na]^+$ ($C_{32}H_{34}O_9SNa$ requires 617.1816).

^{1}H NMR of S1 (400 MHz, $CDCl_3$)

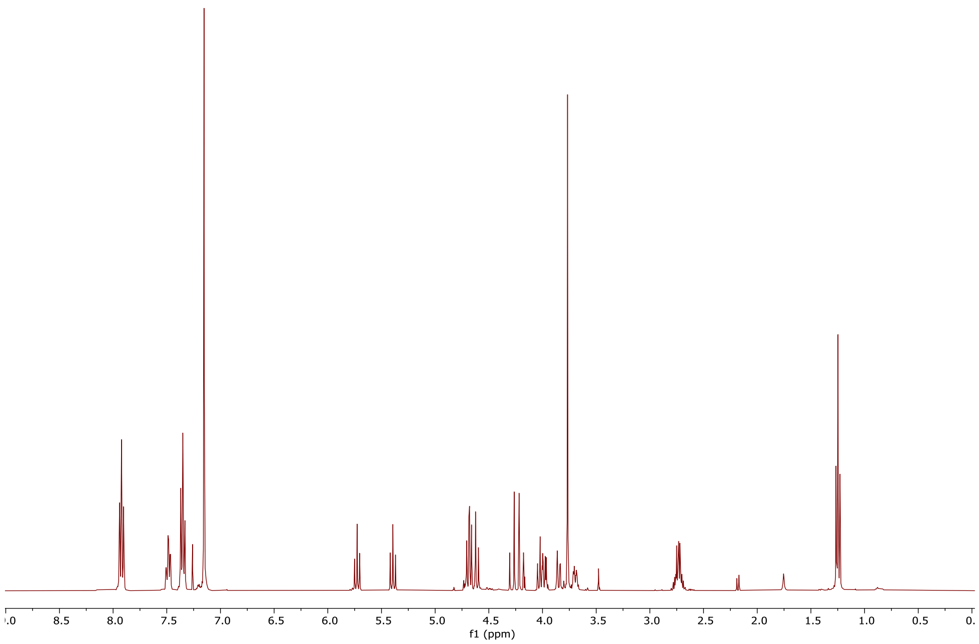

^{13}C NMR of S1 (101 MHz, $CDCl_3$)

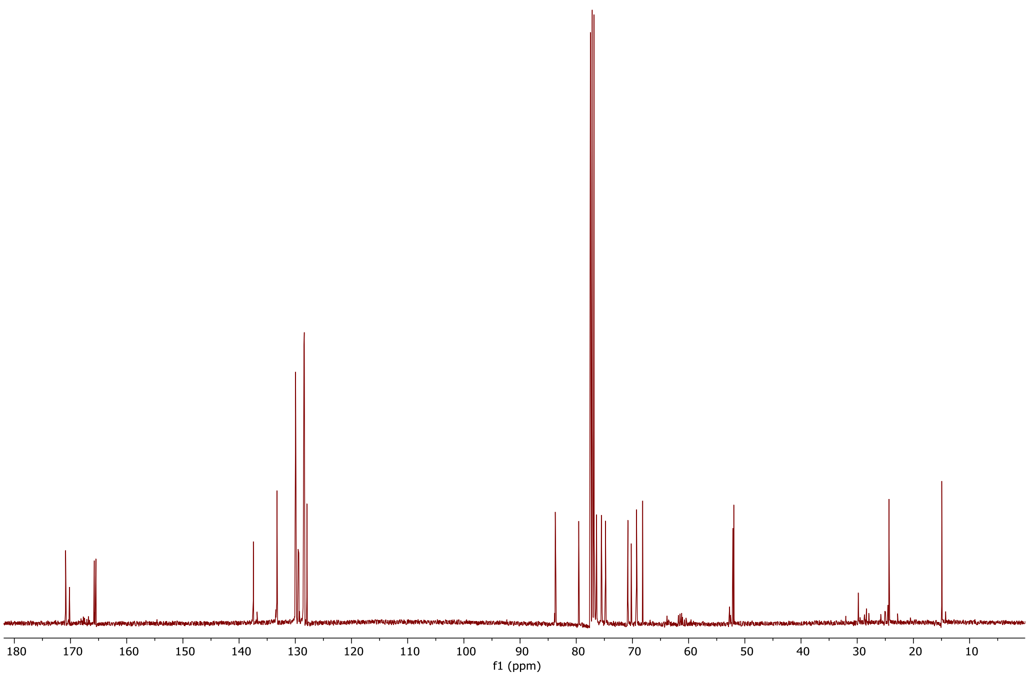

HSQC NMR of S1 ($CDCl_3$)

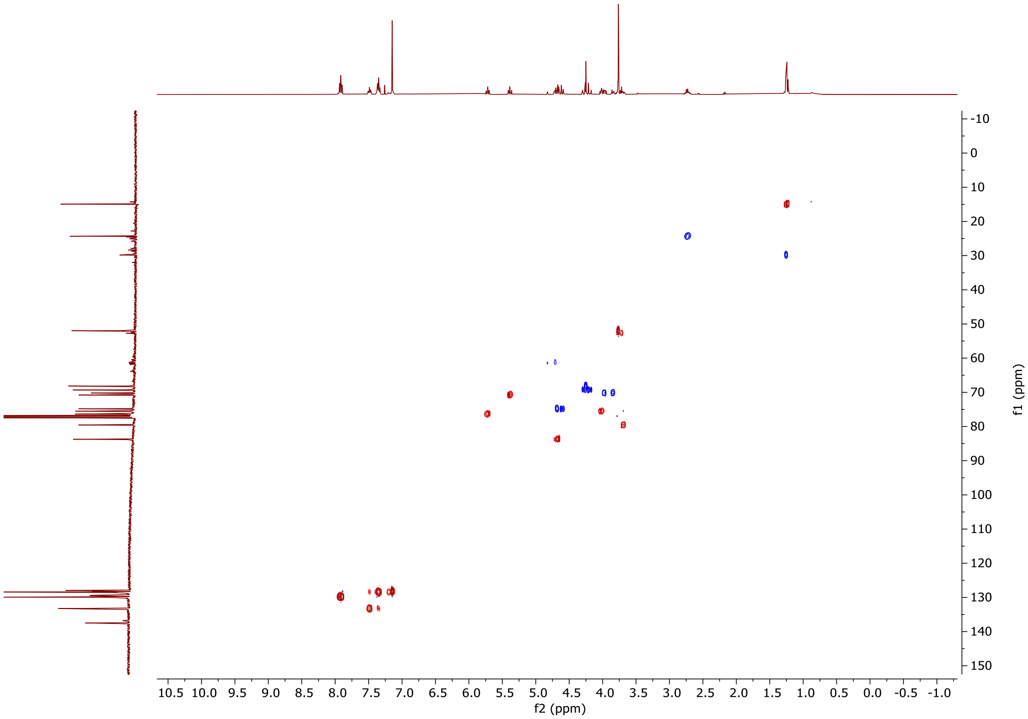

*Synthesis of **2***

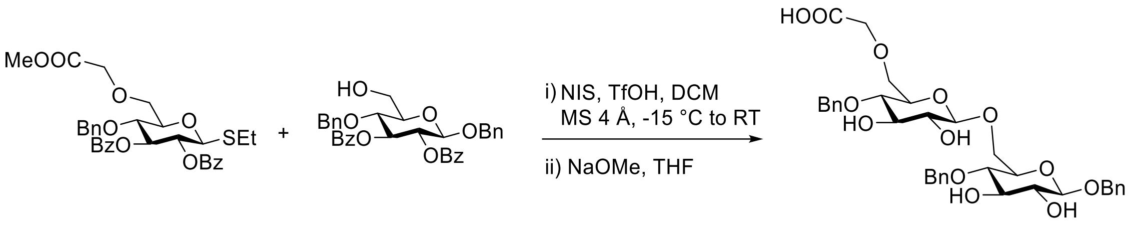

S1 **S2** **2**

S1 (17.0 mg, 0.0286 mmol), **S2**[3] (16.0 mg, 0.0282 mmol), and *N*-iodosuccinimide (6.6 mg, 0.029 mmol) were dissolved in anhydrous DCM (2.0 mL). The solution was stirred with molecular sieve (4 Å) for 1 h at RT under N_2 atmosphere and then cooled to -15 °C. A 1% solution of TfOH in DCM (10 μL) was added and the reaction was stirred for 30 min at -15 °C before the removal of cooling bath to allow the reaction to RT. After TLC indicated the disappearance of **S3**, the reaction was diluted with DCM (10 mL) and washed with H_2O, then brine. The organic layer was dried over Na_2SO_4, filtered, and evaporated. The resulting yellow oil was purified by column chromatography (hexane:EtOAc = 3:1) to give the fully protected disaccharide as white solid, which was then dissolved in THF (2 mL). MeONa in MeOH (0.5 M, 3 equiv. per benzoyl ester) was added to the solution and the mixture was stirred at RT overnight, neutralized with Amberlite IR-120 (H^+ form) resin, filtered, and concentrated *in vacuo*. The resulting yellow oil was purified by column chromatography (DCM:MeOH = 20:1) to give **2** as white solid (7.2 mg, 38%). ^1H NMR (600 MHz, CD_3OD) δ 7.43 (d, *J* = 7.5 Hz, 2H), 7.39 (d, *J* = 7.3 Hz, 4H), 7.33 (td, *J* = 7.7, 2.5 Hz, 6H), 7.30 – 7.25 (m, 3H), 4.97 (d, *J* = 11.1 Hz, 1H), 4.93 (d, *J* = 11.5 Hz, 2H), 4.73 – 4.65 (m, 3H), 4.38 (d, *J* = 7.8 Hz, 1H), 4.31 (d, *J* = 7.8 Hz, 1H), 4.14 – 4.03 (m, 3H), 3.79 – 3.70 (m, 3H), 3.60 – 3.55 (m, 1H), 3.53 (t, *J* = 9.0 Hz, 2H), 3.50 – 3.34 (m, 4H), 3.34 – 3.25 (m, 5H).^{13}C NMR (151 MHz, CD_3OD) δ 174.03, 140.18, 140.10, 139.23, 129.42,

129.39, 129.38, 129.29, 129.26, 128.77, 105.12, 103.47, 79.53, 79.12, 78.60, 78.47, 77.31, 76.22, 75.78, 75.75, 75.48, 75.29, 72.06, 71.61, 69.90, 69.66, 67.75, 67.50, 49.53, 49.38, 49.24, 49.10, 48.96, 48.82, 48.67. (ESI-HRMS) m/z 693.2551 $[M+Na]^+$ ($C_{35}H_{42}O_{13}Na$ requires 693.2518).

^1H NMR of 2 (600 MHz, CD_3OD)

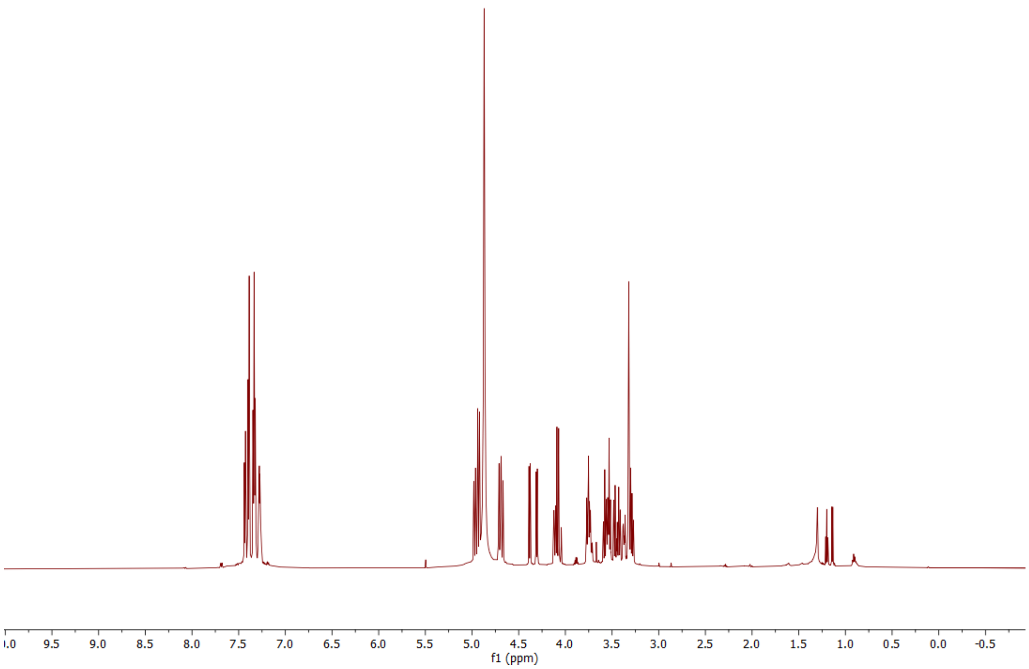

^{13}C NMR of 2 (151 MHz, CD_3OD)

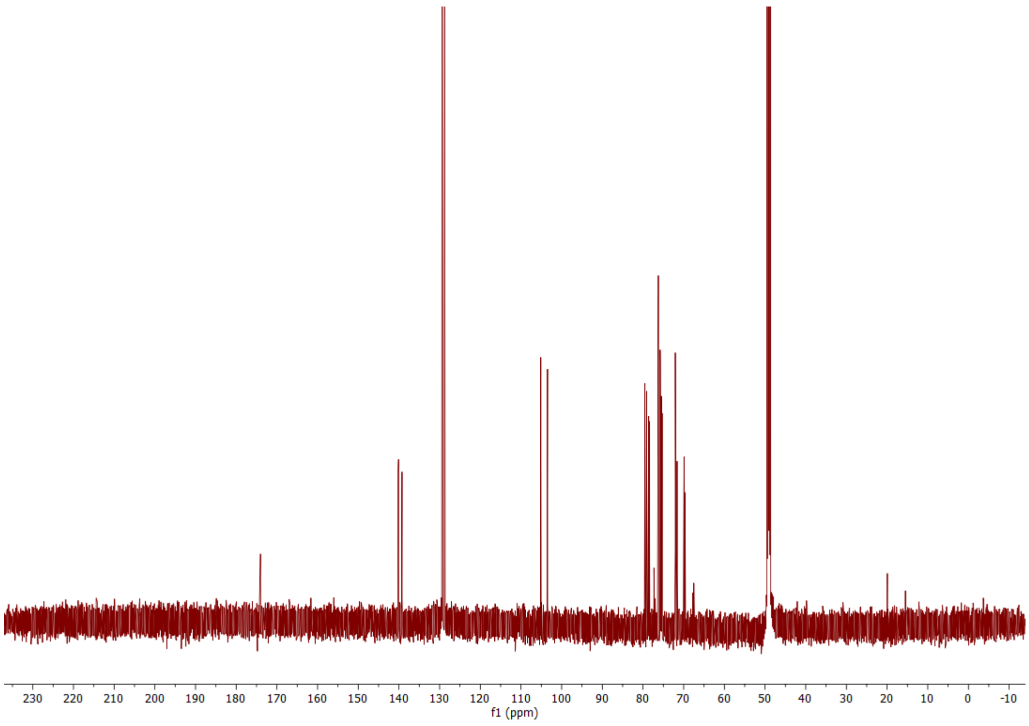

HSQC NMR of 2 (CD_3OD)

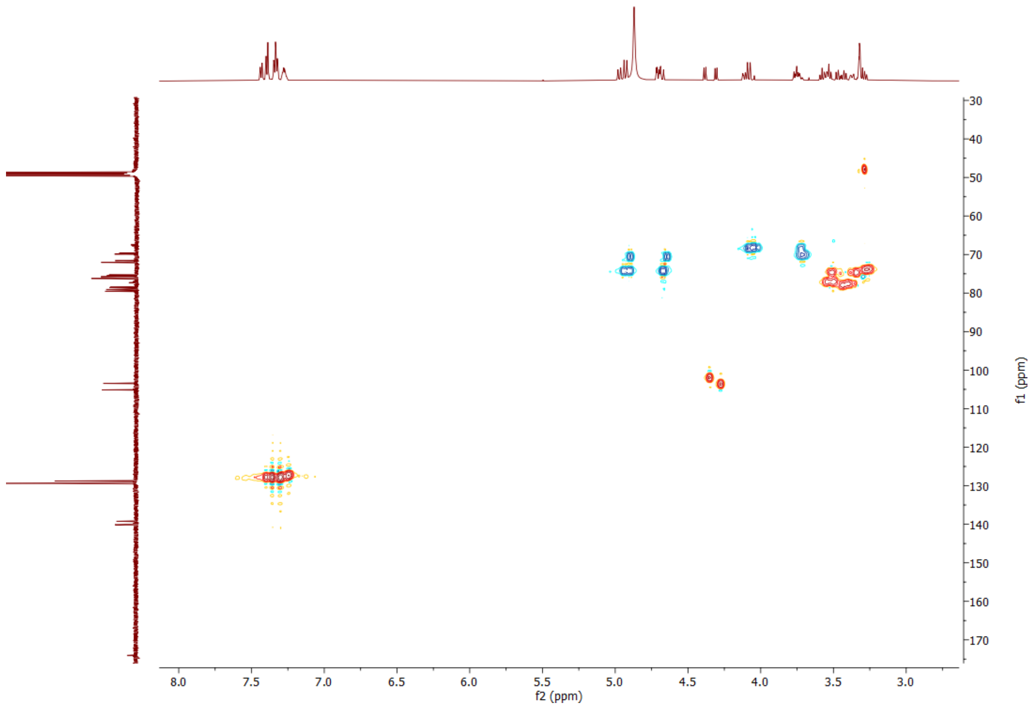

2.2. Synthesis of 3

Synthesis of ***S4***

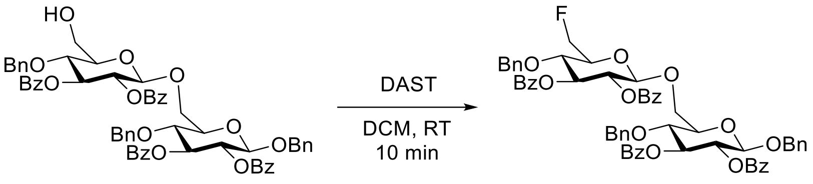

S3 **S4**

S3 was synthesized according to a previously reported procedure.[3]

S3 (30.0 mg, 0.029 mmol) was dissolved in DCM (2 mL). DAST (7.8 µL, 0.058 mmol) was added dropwise to the reaction mixture at RT. The solution was stirred for 10 min and quenched with MeOH. The crude reaction mixture was diluted with DCM, washed with brine once, and then concentrated. The crude compound was purified with flash column chromatography (Hexane:EtOAc = 2:1) to give **S4** as a colorless oil (25.2 mg, 84%). ^{1}H NMR (400 MHz, $CDCl_3$) δ 7.87 (ddd, *J* = 9.7, 7.6, 1.3 Hz, 4H), 7.80 (ddd, *J* = 8.5, 5.2, 1.4 Hz, 4H), 7.45 – 7.39 (m, 3H), 7.34 – 7.30 (m, 3H), 7.30 – 7.24 (m, 4H), 7.21 – 7.15 (m, 3H), 7.14 – 7.10 (m, 4H), 7.07 (dddd, *J* = 6.6, 5.5, 4.1, 1.5 Hz, 8H), 6.93 (dq, *J* = 5.3, 2.1, 1.6 Hz, 2H), 5.68 (t, *J* = 9.3 Hz, 1H), 5.54 – 5.47 (m, 1H), 5.40 (dd, *J* = 9.6, 7.7 Hz, 1H), 5.29 (dd, *J* = 9.8, 7.9 Hz, 1H), 4.72 (t, *J* = 6.2 Hz, 2H), 4.65 – 4.56 (m, 2H), 4.55 – 4.47 (m, 3H), 4.41 (d, *J* = 12.7 Hz, 1H), 4.29 (s, 2H), 4.14 (dd, *J* = 11.2, 1.8 Hz, 1H), 3.93 (t, *J* = 9.5 Hz, 1H), 3.75 – 3.59 (m, 3H), 3.58 – 3.52 (m, 1H); ^{13}C NMR (101 MHz, $CDCl_3$) δ 165.74, 165.60, 165.28, 165.21, 137.12, 136.93, 136.86, 133.36, 133.23, 133.19, 133.11, 129.92, 129.89, 129.86, 129.80, 129.77, 129.42, 129.34, 129.27, 129.24, 128.51, 128.48, 128.44, 128.41, 128.37, 128.32, 128.31, 128.28, 128.25, 128.13, 127.97, 127.95, 127.87, 127.80, 127.75, 101.03, 99.04, 82.38, 80.64, 77.28, 76.09, 75.09, 74.99, 74.95, 74.88, 74.64, 74.41, 74.22, 71.98, 71.91, 70.12, 67.99. ^{19}F NMR (376

MHz, $CDCl_3$) δ -232.86 (td, *J* = 47.4, 26.7 Hz). (ESI-HRMS) m/z 1053.375 [M + Na]$^+$ ($C_{61}H_{55}FO_{14}Na$ requires 1053.347).

^{1}H NMR of S4 (400 MHz, $CDCl_3$)

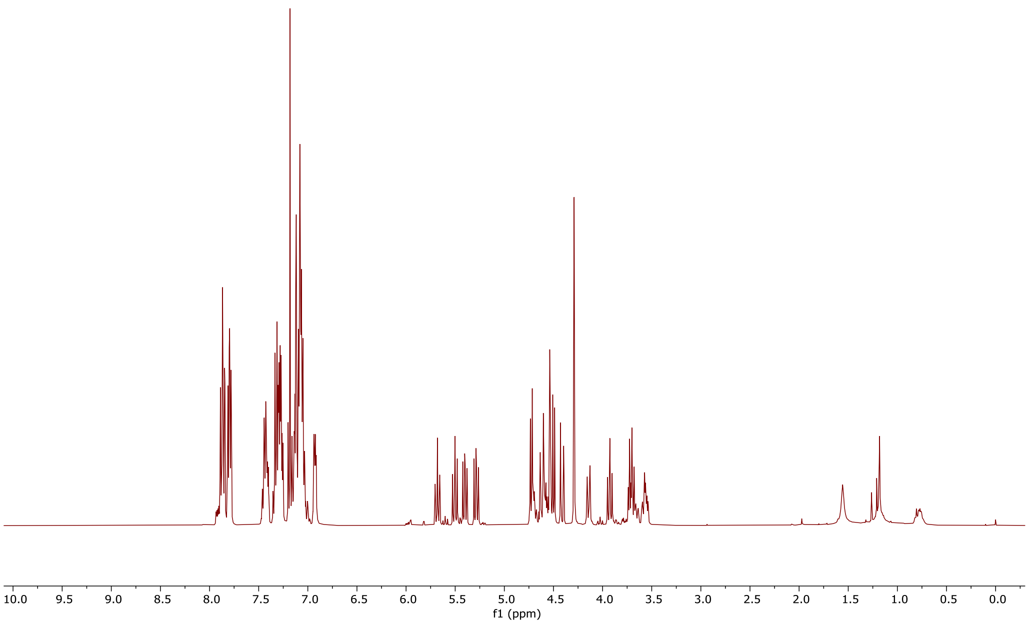

^{13}C NMR of S4 (101 MHz, $CDCl_3$)

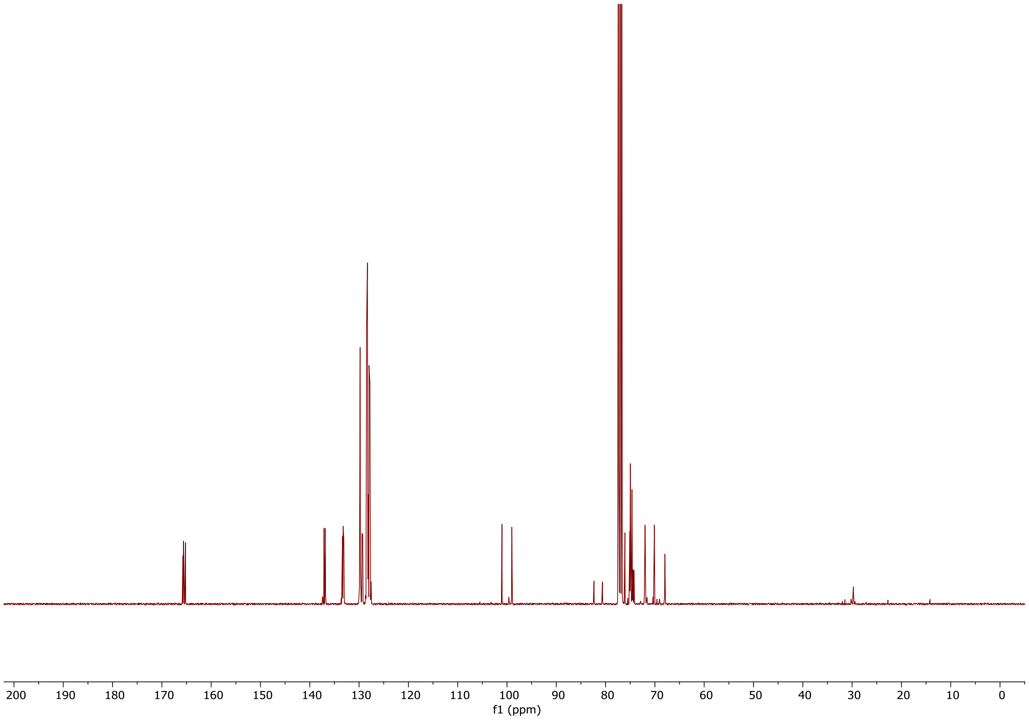

^{19}F NMR of S4 (376 MHz, $CDCl_3$)

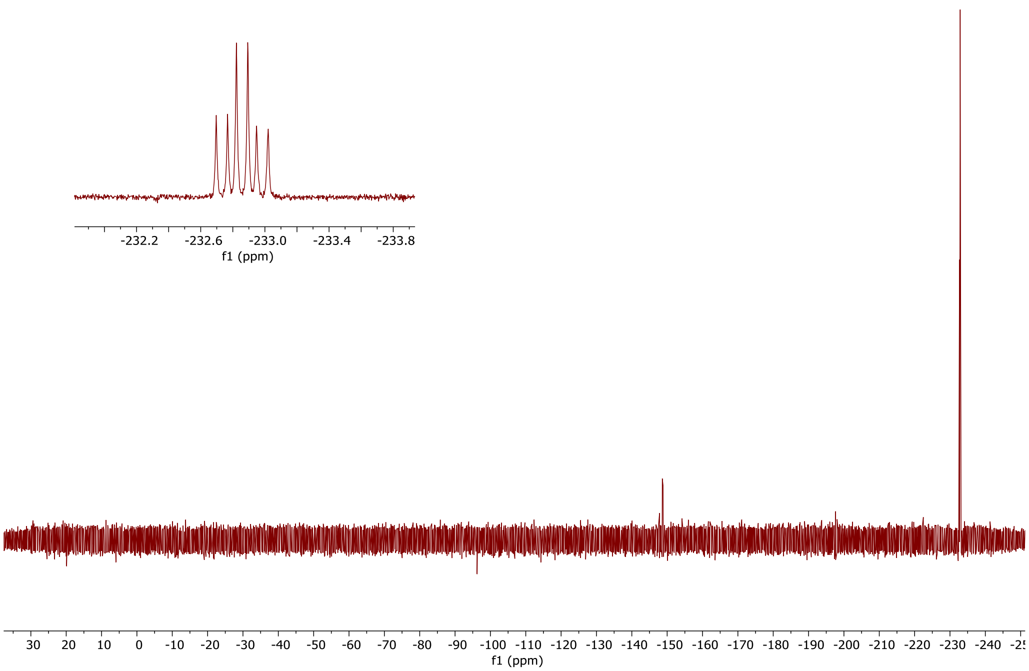

HSQC NMR of S4 ($CDCl_3$)

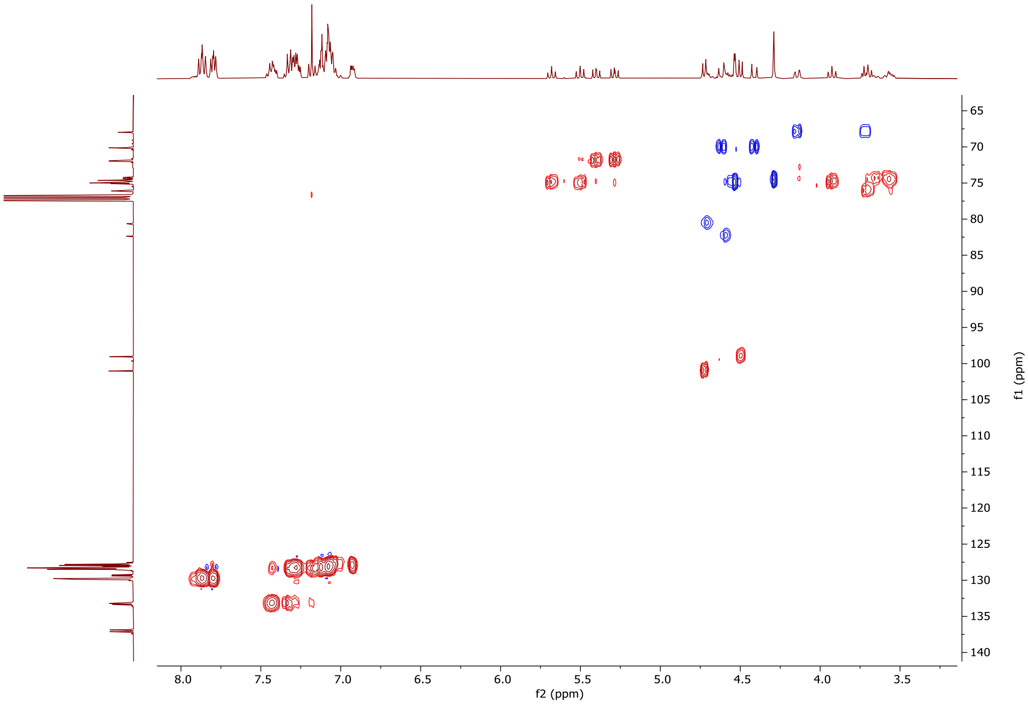

*Synthesis of **3***

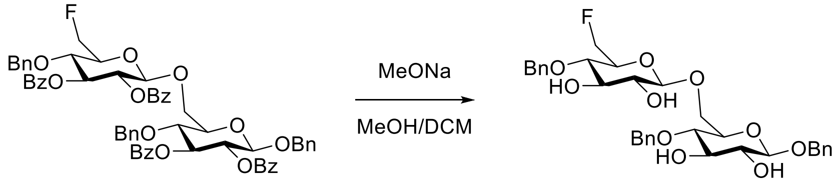

S4 **3**

S4 (25.2 mg, 0.0245 mmol) was dissolved in a 1:1 mixture of DCM:MeOH (4 mL). A solution of NaOMe (0.5 mL, 0.5 M in MeOH) was added. The solution was stirred overnight at RT and then quenched with Amberlite IR-120 (H^+ form) resin, filtered, and concentrated. The crude compound was purified with flash column chromatography (DCM:MeOH = 25:1) to give **3** as white solid (10.6 mg, 70%). ^{1}H NMR (400 MHz, $CDCl_3$) δ 7.25 (h, *J* = 7.5, 6.2 Hz, 15H), 4.89 – 4.77 (m, 3H), 4.65 – 4.39 (m, 5H), 4.28 (d, *J* = 7.5 Hz, 1H), 4.18 (d, *J* = 7.7 Hz, 1H), 4.09 (dd, *J* = 11.8, 2.3 Hz, 1H), 3.69 (dd, *J* = 11.8, 4.4 Hz, 1H), 3.62 (td, *J* = 9.0, 3.5 Hz, 2H), 3.56 – 3.43 (m, 2H), 3.38 (t, *J* = 7.7 Hz, 3H), 3.25 (ddd, *J* = 26.9, 9.8, 3.2 Hz, 1H), 2.78 (s, 4H); ^{13}C NMR (101 MHz, $CDCl_3$) δ 138.21, 138.00, 136.82, 128.56, 128.24, 128.12, 128.09, 128.02, 127.93, 127.91, 103.09, 101.46, 82.70, 80.98, 77.26, 76.46, 76.19, 76.13, 74.82, 74.61, 74.49, 74.26, 74.08, 73.76, 73.35, 71.27, 67.55. ^{19}F NMR (376 MHz, $CDCl_3$) δ -232.84 (td, *J* = 47.9, 25.8 Hz). (ESI-HRMS) m/z 637.2604 [M + Na]$^+$ ($C_{33}H_{39}FO_{10}Na$ requires 637.2419).

^{1}H NMR of 3 (400 MHz, $CDCl_3$)

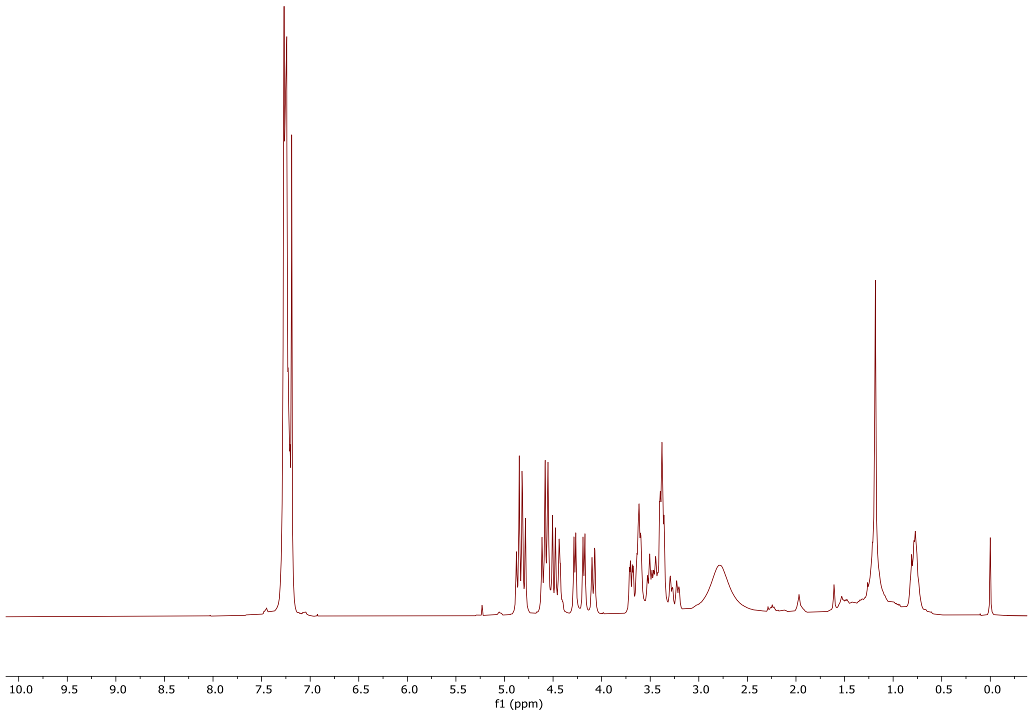

^{13}C NMR of 3 (101 MHz, $CDCl_3$)

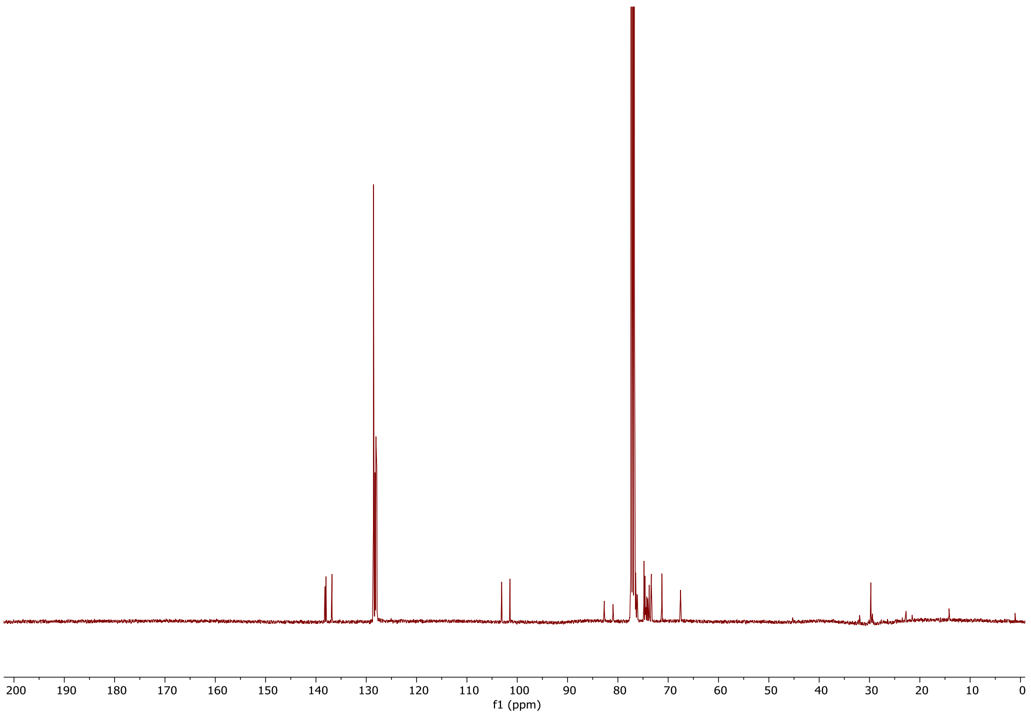

^{19}F NMR of 3 (376 MHz, $CDCl_3$)

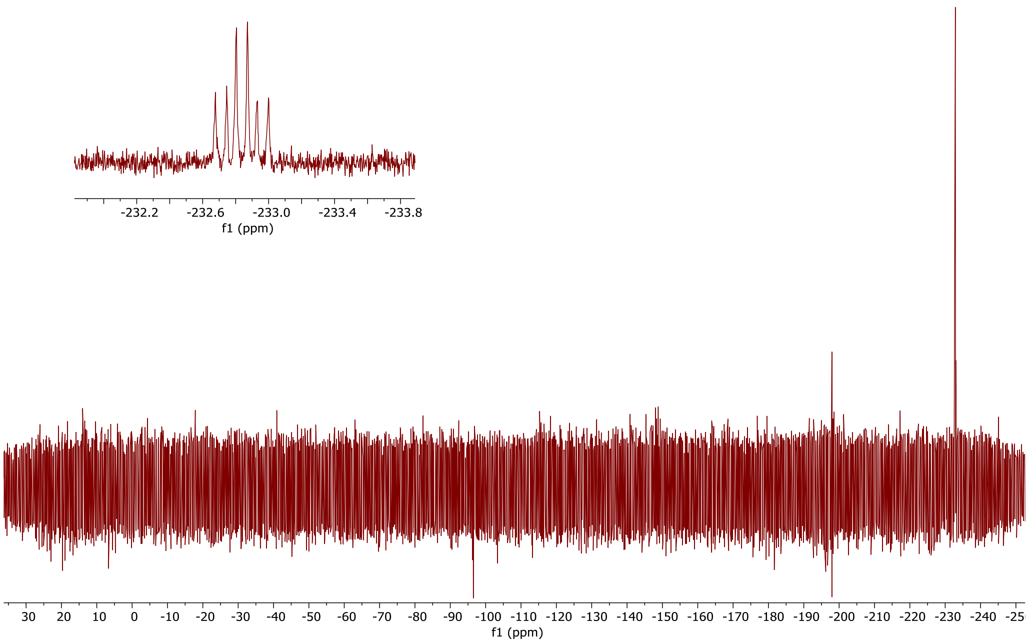

HSQC NMR of 3 ($CDCl_3$)

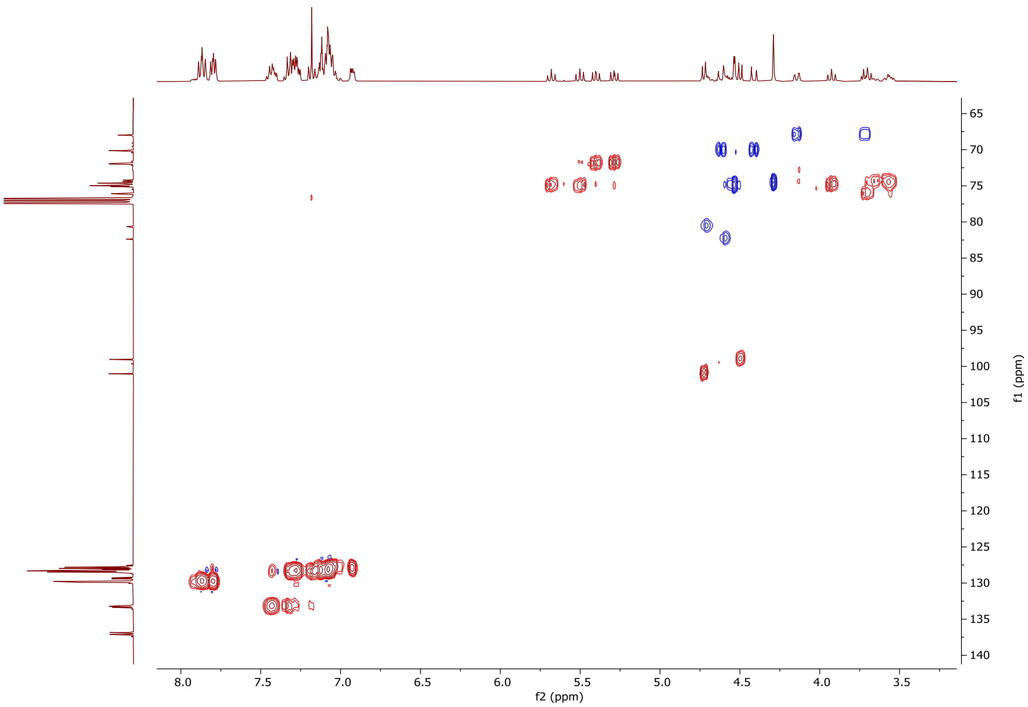

2.3. Synthesis of 4

*Synthesis of **S6***

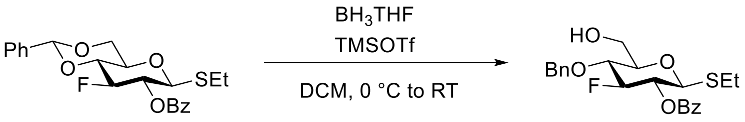

S5 **S6**

S5 was prepared according to previously established procedures.[5].

Ethyl 2-*O*-benzoyl-4,6-*O*-benzylidene-3-deoxy-3-fluoro-1-thio-β-D-glucopyranoside **S5** (460 mg, 1.1 mmol) was dissolved in DCM (20 mL). A 0.5 M solution of BH_3-THF in THF (2.0 mL, 12.5 mmol) and TMSOTf (100 µL, 0.55 mmol) were sequentially added to the stirred solution at 0 °C. The reaction was allowed to RT and, after 4 h, quenched with $NaHCO_3$ sat. aq. solution. The organic layer was washed twice with $NaHCO_3$ sat. aq. solution and once with brine. The crude product was purified by flash column chromatography on silica (Hex/EtOAc 4:1→2:1) to give **S6** as a white solid (432 mg, 92%). ^{1}H NMR (400 MHz, $CDCl_3$) δ 8.12 – 8.05 (m, 2H), 7.63 – 7.57 (m, 1H), 7.47 (t, *J* = 7.8 Hz, 2H), 7.41 – 7.28 (m, 5H), 5.43 – 5.30 (m, 1H), 4.96 – 4.73 (m, 2H), 4.67 (d, *J* = 11.1 Hz, 1H), 4.58 (d, *J* = 10.1 Hz, 1H), 4.00 – 3.90 (m, 1H), 3.88 – 3.73 (m, 2H), 3.51 – 3.44 (m, 1H), 2.79 – 2.64 (m, 2H), 1.24 (t, *J* = 7.4 Hz, 3H); ^{13}C NMR (101 MHz, $CDCl_3$) δ 165.35, 137.47, 133.56, 130.07, 129.50, 128.69, 128.59, 128.44, 128.31, 96.75 (d, *J* = 189.9 Hz), 83.06 (d, *J* = 8.0 Hz), 78.64 (d, *J* = 8.2 Hz), 75.47 (d, *J* = 16.5 Hz), 74.76 (d, *J* = 2.9 Hz), 70.69 (d, *J* = 18.6 Hz), 61.97, 24.40, 14.98. ^{19}F NMR (376 MHz, $CDCl_3$) δ -187.82 (dt, *J* = 52.5, 12.6 Hz); $[\alpha]_D^{20}$ +28.14 (*c* 0.62 g/100 mL, $CHCl_3$) IR *v* = 3480, 2931, 1728, 1264, 1089, 1071, 1027, 710 cm^{-1}. (ESI-HRMS+) m/z 443.1303 $[M+Na]^+$ ($C_{22}H_{25}FO_5SNa$ requires 443.1304).

^{1}H NMR of S6 (400 MHz, $CDCl_3$)

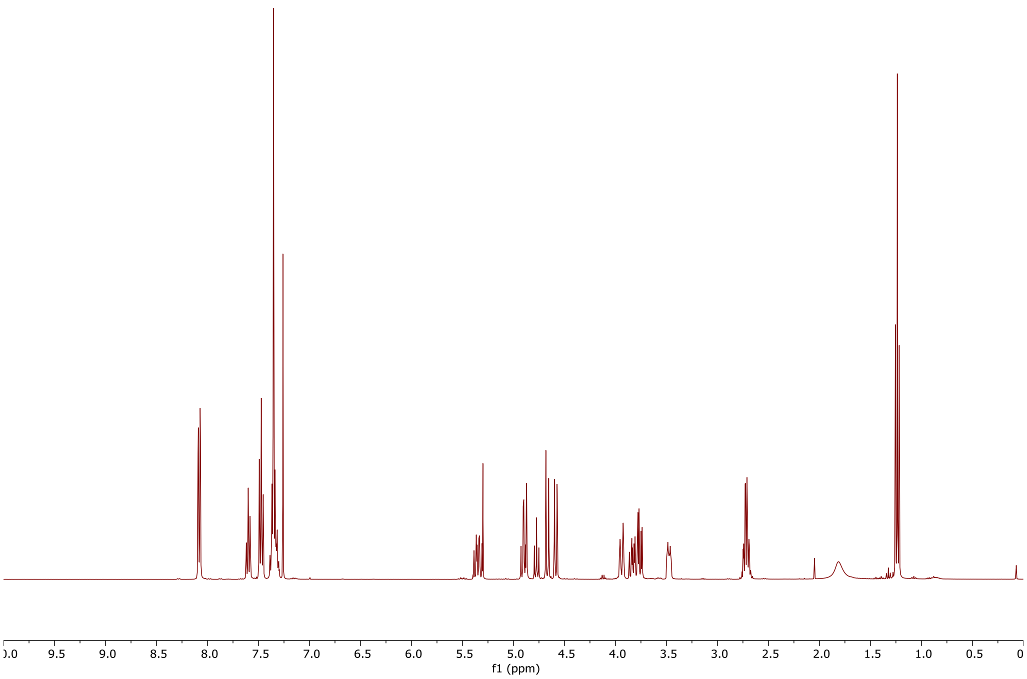

^{13}C NMR of S6 (101 MHz, $CDCl_3$)

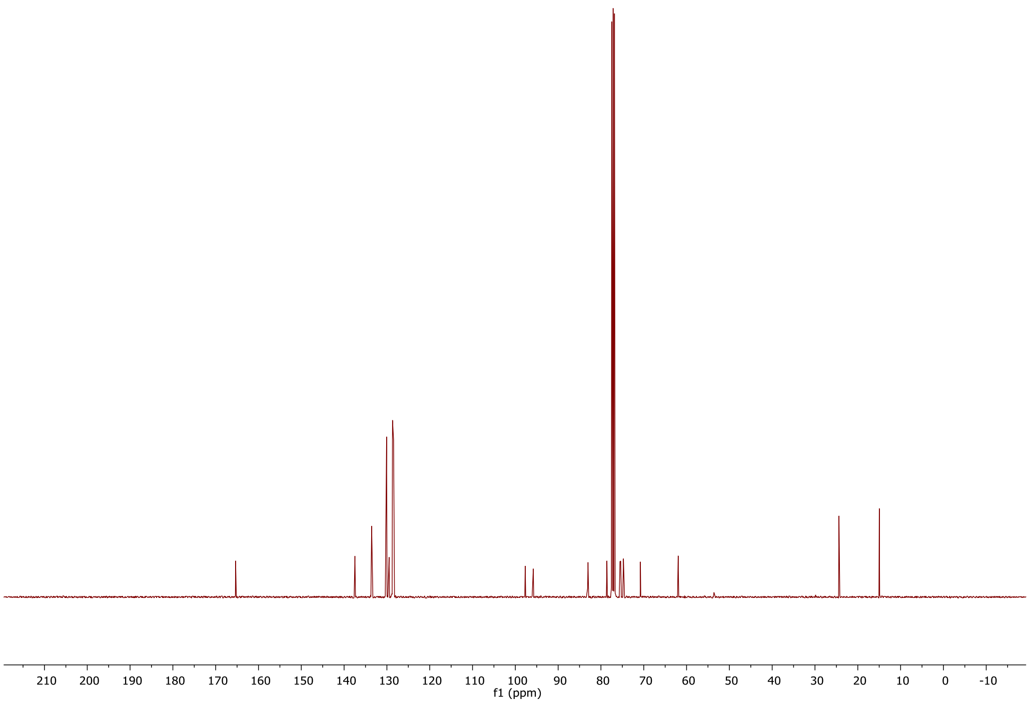

^{19}F NMR of S6 (376 MHz, $CDCl_3$)

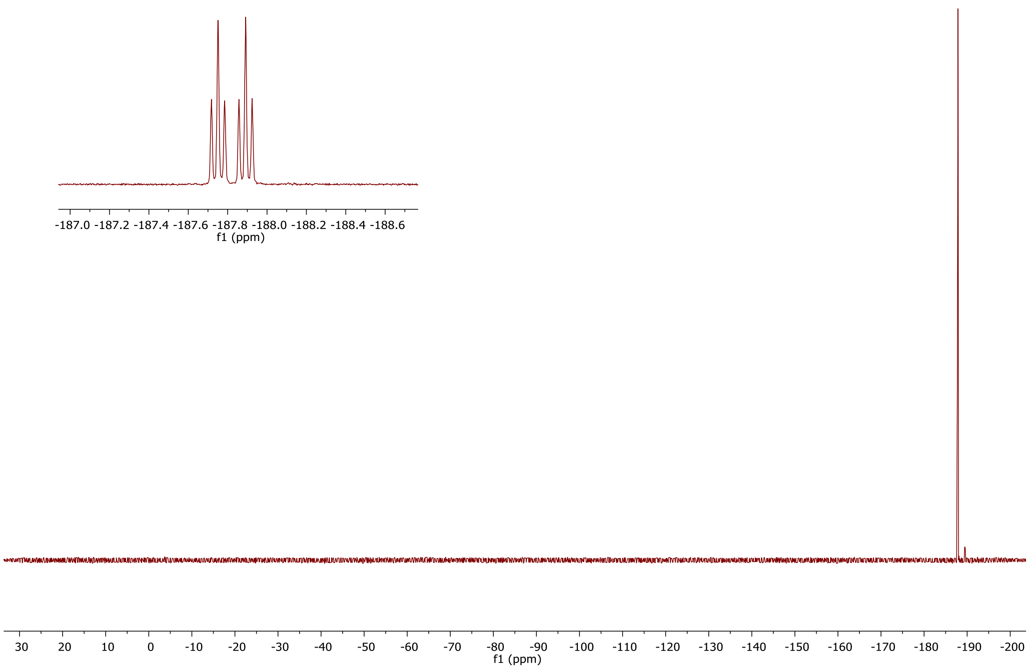

HSQC NMR of S6 ($CDCl_3$)

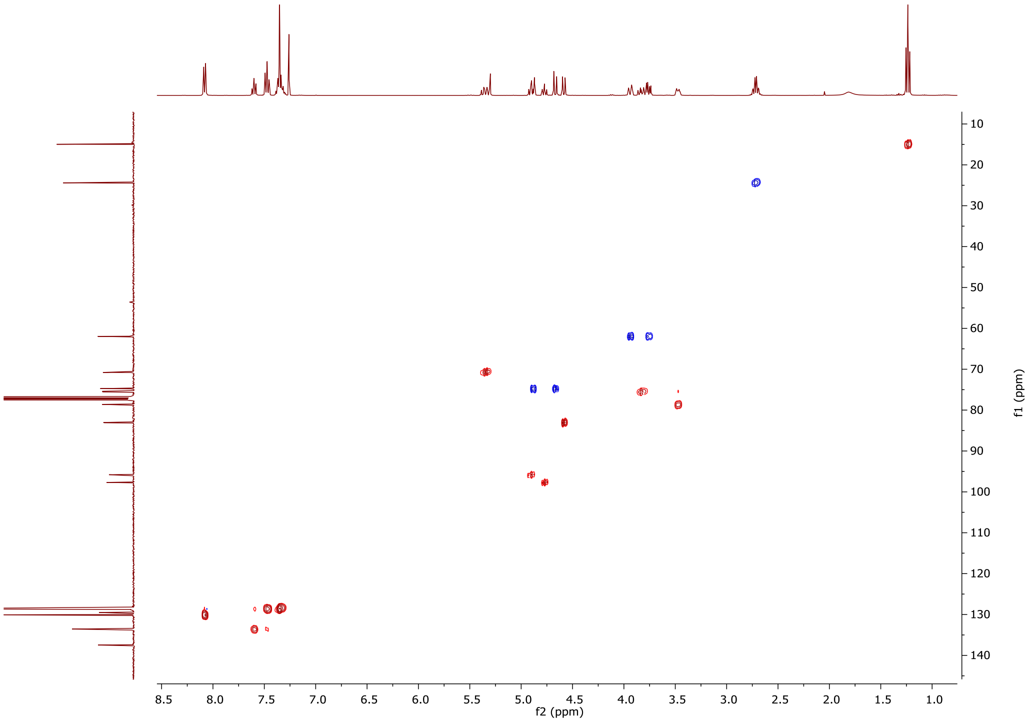

*Synthesis of **S7***

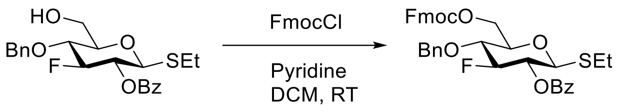

S6 **S7**

Ethyl 2-*O*-benzoyl-4-*O*-benzyl-3-deoxy-3-fluoro-1-thio-β-D-glucopyranoside, **S6** (430 mg, 1.0 mmol) was dissolved in DCM (20 mL) and pyridine was added (250 µL, 3.1 mmol). FmocCl (507 mg, 1.9 mmol) was dissolved in DCM (2 mL) and added to the reaction mixture under Ar atmosphere at RT. The yellow solution was stirred for 2 h then quenched with a 1 M solution of HCl. The organic layer was washed one time with 1 M HCl, one time with sat. aq. solution of $NaHCO_3$ and one time with brine. The crude compound was purified with flash column chromatography (Hex/EtOAc = 4:1→2:1) to give **S7** as a white solid (430 mg, 65%). ^{1}H NMR (400 MHz, $CDCl_3$) δ 8.14 – 8.06 (m, 2H), 7.78 (d, *J* = 7.5 Hz, 1H), 7.67 – 7.57 (m, 3H), 7.53 – 7.39 (m, 4H), 7.39 – 7.27 (m, 7H), 5.47 – 5.34 (m, 1H), 4.97 – 4.74 (m, 2H), 4.64 (d, *J* = 11.1 Hz, 1H), 4.61 – 4.54 (m, 1H), 4.51 (dt, *J* = 11.4, 1.7 Hz, 1H), 4.47 – 4.29 (m, 3H), 4.27 (t, *J* = 7.4 Hz, 1H), 3.88 – 3.75 (m, 1H), 3.71 – 3.61 (m, 1H), 2.81 – 2.62 (m, 2H), 1.23 (t, *J* = 7.5 Hz, 3H). ^{13}C NMR (101 MHz, $CDCl_3$) δ 165.33, 155.01, 143.44, 143.37, 141.41, 137.17, 133.58, 130.09, 129.49, 128.71, 128.60, 128.53, 128.37, 128.07, 127.33, 125.34, 125.30, 120.22, 96.85 (d, *J* = 189.8 Hz), 82.96 (d, *J* = 8.1 Hz), 76.21 (d, *J* = 9.3 Hz), 75.27 (d, *J* = 16.9 Hz), 74.69 (d, *J* = 3.1 Hz), 70.49 (d, *J* = 18.5 Hz), 70.19, 66.39, 46.79, 24.33, 15.02. ^{19}F NMR (376 MHz, $CDCl_3$) δ -187.62 (dt, *J* = 52.2, 12.5 Hz). $[\alpha]_D^{20}$ +20.30 (*c* 1.2 g/100 mL, $CHCl_3$) IR *ν* = 2929, 1731, 1452, 1251, 1092, 740, 709 cm^{-1}. (ESI-HRMS+) m/z 665.1991 $[M+Na]^+$ ($C_{37}H_{35}FO_7SNa$ requires 665.1985).

^{1}H NMR of S7 (400 MHz, $CDCl_3$)

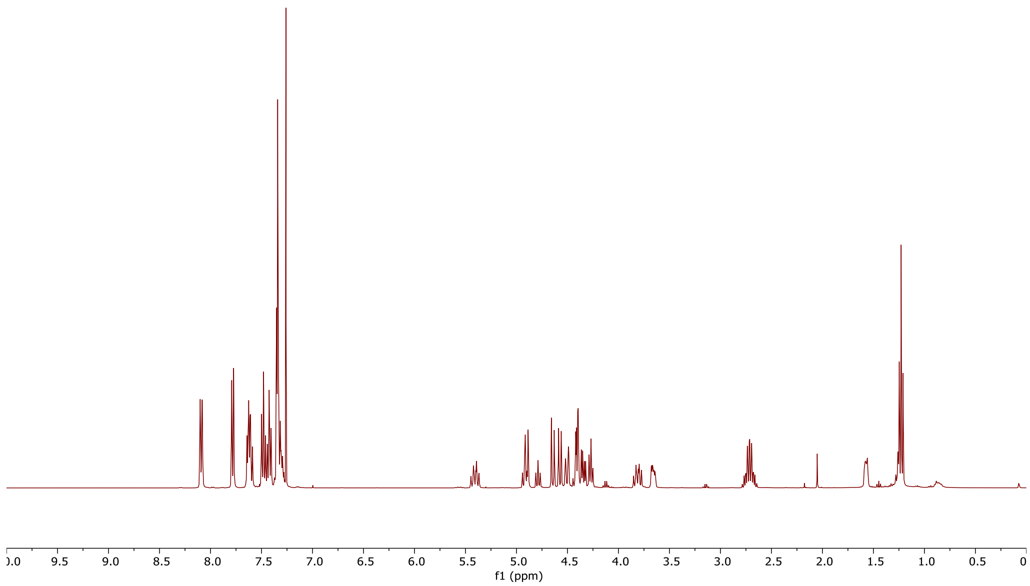

^{13}C NMR of S7 (101 MHz, $CDCl_3$)

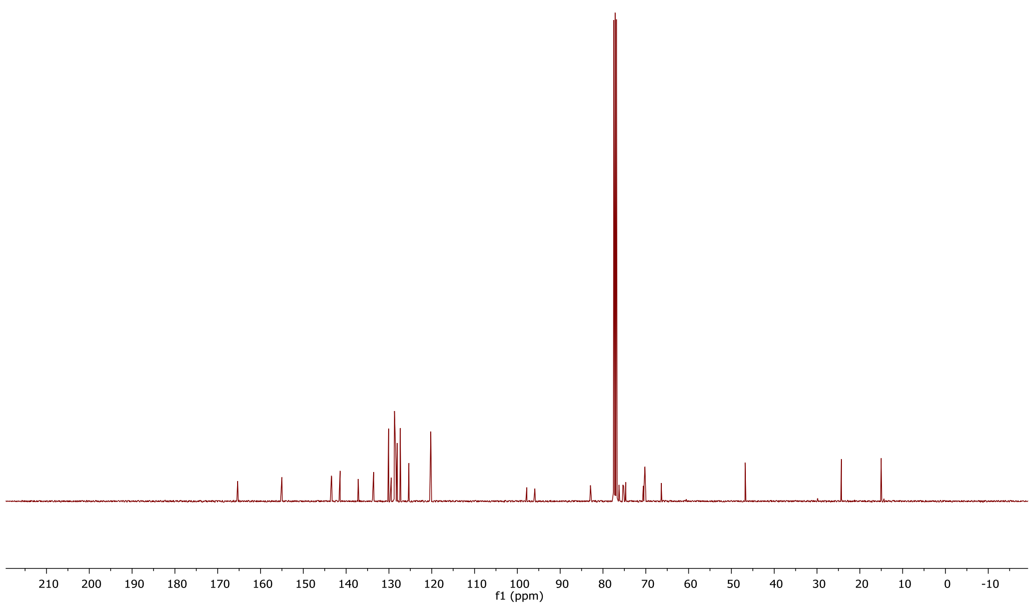

^{19}F NMR of S7 (376 MHz, $CDCl_3$)

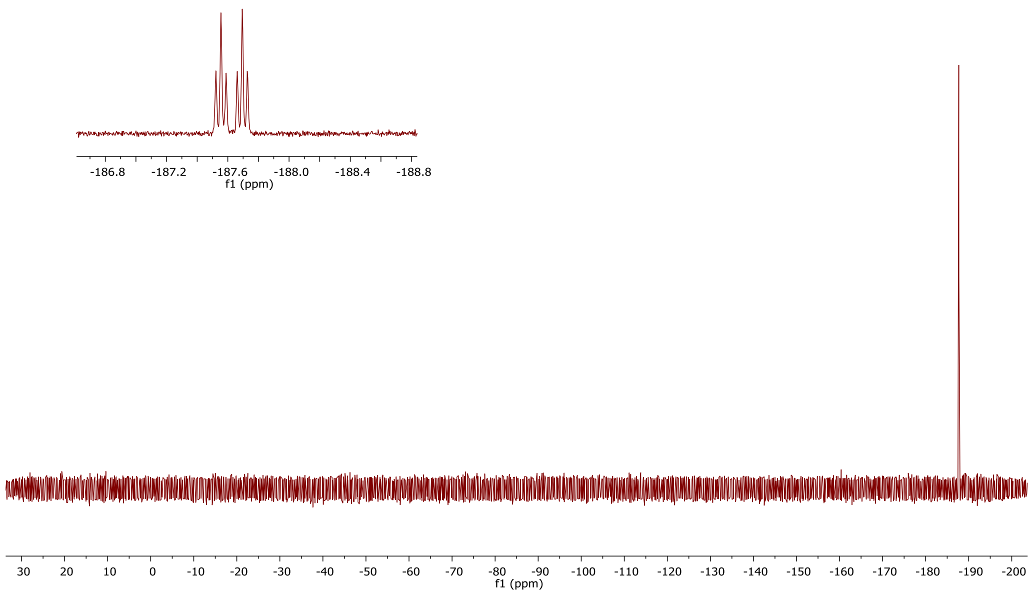

HSQC NMR of S7 ($CDCl_3$)

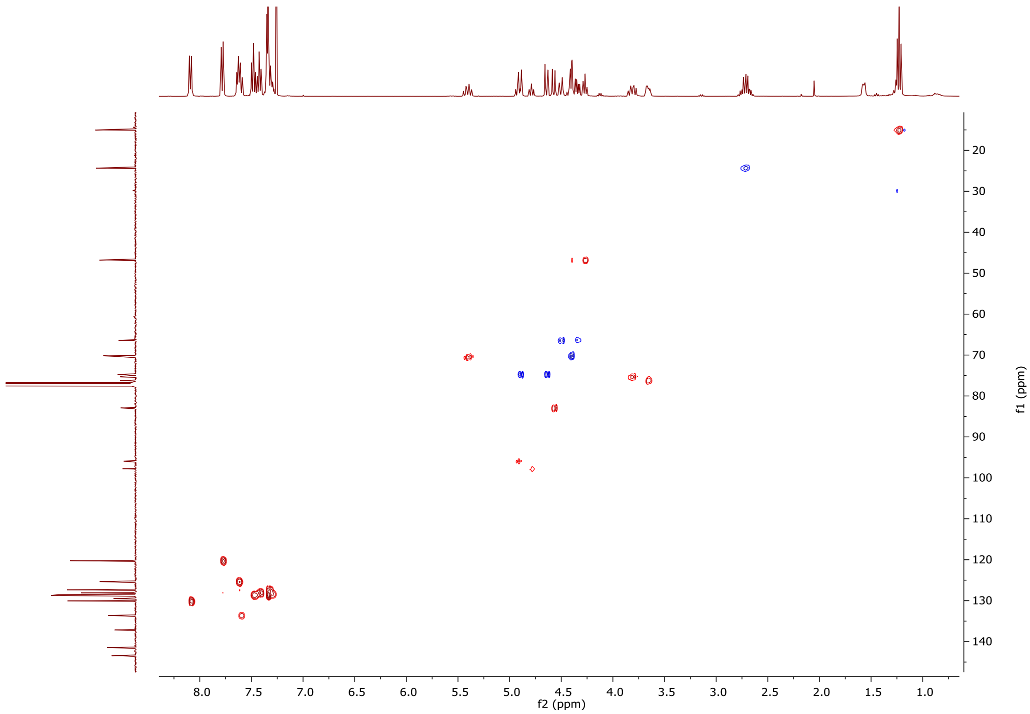

Synthesis of ***S8***

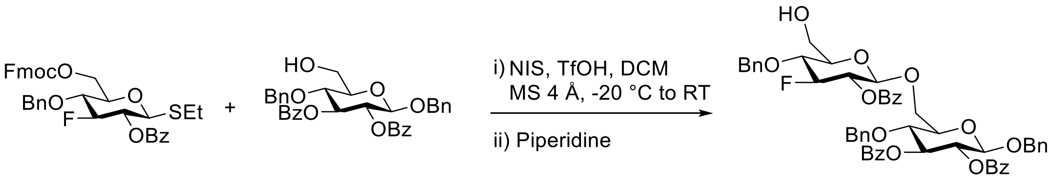

S7 **S2** **S8**

S7 (110 mg, 0.171 mmol) and **S2** (79 mg, 0.139 mmol) were dissolved in anhydrous DCM (1.3 mL). The solution was stirred with molecular sieves (4 Å) for 1 h at RT under Ar atmosphere and then cooled to -20 °C. The activator solution (350 µL of a 133 mg/mL solution of NIS in DCM:dioxane 2:1 with 1% TfOH) was added dropwise and the reaction was stirred for 5 min at -20 °C, after which time the cooling bath was removed to allow the reaction to RT. After 1 h stirring at RT, ESI-MS indicated the disappearance of **S7**. Piperidine (0.5 mL) was added and the reaction was stirred at RT for additional 1 h. The reaction was diluted with DCM and washed once with 1 M HCl, once with $NaHCO_3$ sat. aq. solution and once with brine. The crude was purified by column chromatography (Hexane:EtOAc = 3:1→2:1→1:1→2:3) to give **S8** as a white foam (114 mg, 88%). ^{1}H NMR (400 MHz, $CDCl_3$) δ 8.13 – 8.02 (m, 2H), 7.96 – 7.84 (m, 4H), 7.54 – 7.47 (m, 3H), 7.41 – 7.31 (m, 11H), 7.27 – 7.09 (m, 8H), 7.02 – 6.95 (m, 2H), 5.60 (t, *J* = 9.5 Hz, 1H), 5.48 (ddd, *J* = 13.6, 9.2, 7.9 Hz, 1H), 5.38 (dd, *J* = 9.8, 7.9 Hz, 1H), 4.98 – 4.76 (m, 2H), 4.75 – 4.66 (m, 3H), 4.60 (d, *J* = 7.9 Hz, 1H), 4.48 (d, *J* = 12.7 Hz, 1H), 4.13 (dd, *J* = 11.2, 1.6 Hz, 1H), 4.02 – 3.76 (m, 5H), 3.67 – 3.57 (m, 1H), 3.53 – 3.44 (m, 1H); ^{13}C NMR (101 MHz, $CDCl_3$) δ 165.67, 165.31, 165.18, 137.51, 137.25, 136.82, 133.48, 133.27, 133.17, 100.69 (d, *J* = 11.1 Hz), 99.22, 95.81 (d, *J* = 188.1 Hz), 76.14, 75.63 (d, *J* = 16.7 Hz), 75.17, 74.74, 74.65, 74.37 (d,

J = 8.6 Hz), 72.31 (d, J = 18.8 Hz), 71.94, 70.35, 68.68, 61.84. ^{19}F NMR (376 MHz, $CDCl_3$) δ -191.14 (dt, J = 52.6, 13.5 Hz); (ESI-HRMS) m/z 949.3256 $[M+Na]^+$ ($C_{54}H_{51}FO_{13}Na$ requires 949.3206).

^1H NMR of S8 (400 MHz, $CDCl_3$)

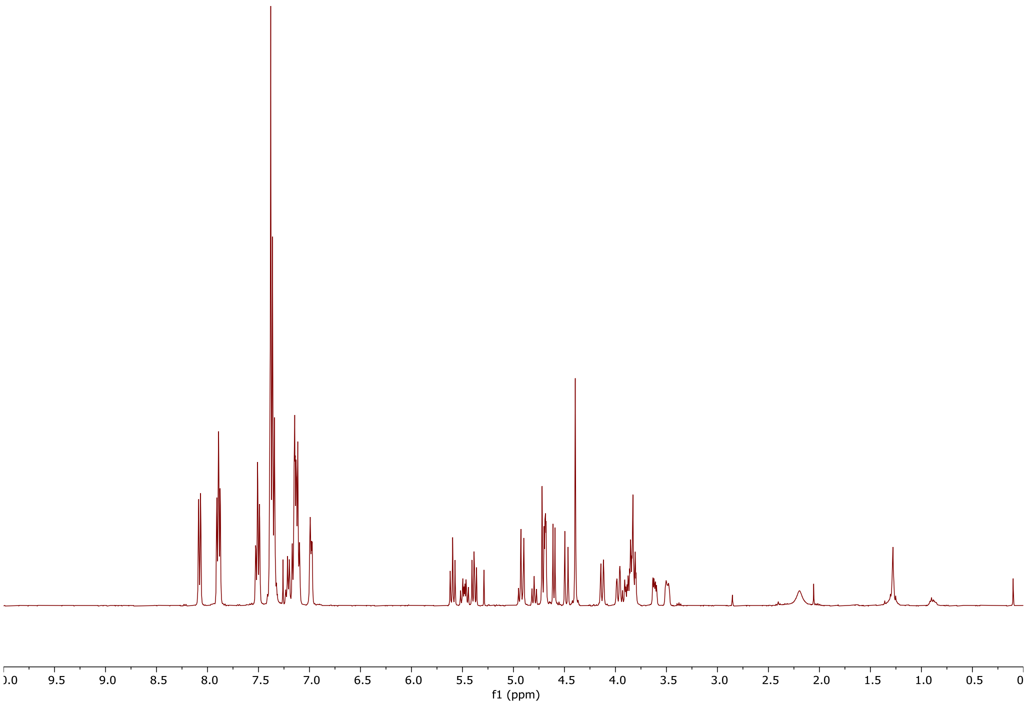

^{13}C NMR of S8 (101 MHz, $CDCl_3$)

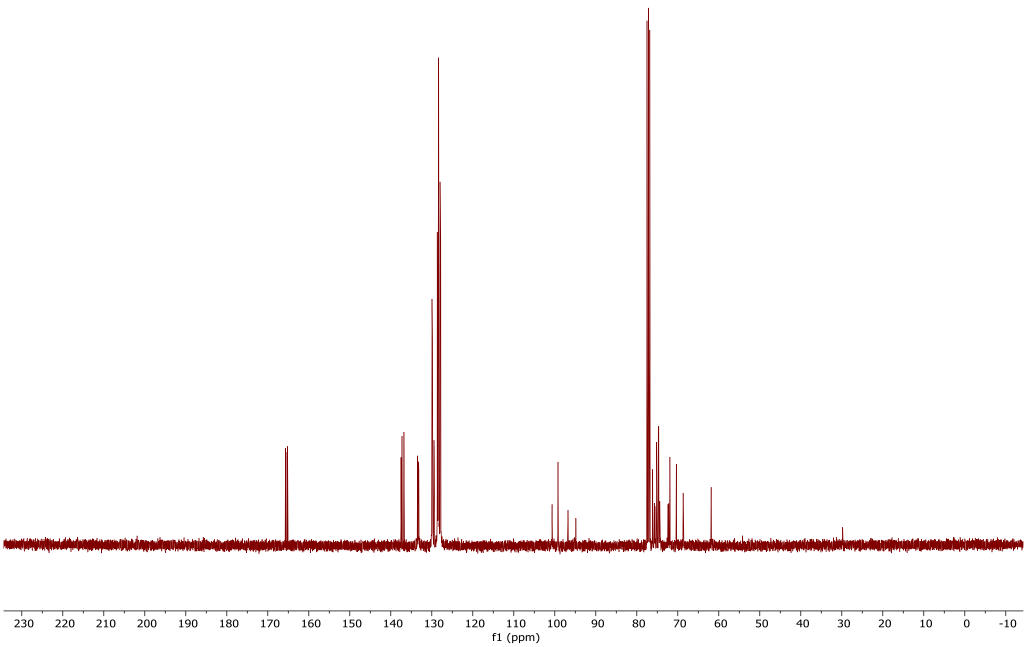

^{19}F NMR of S8 (376 MHz, $CDCl_3$)

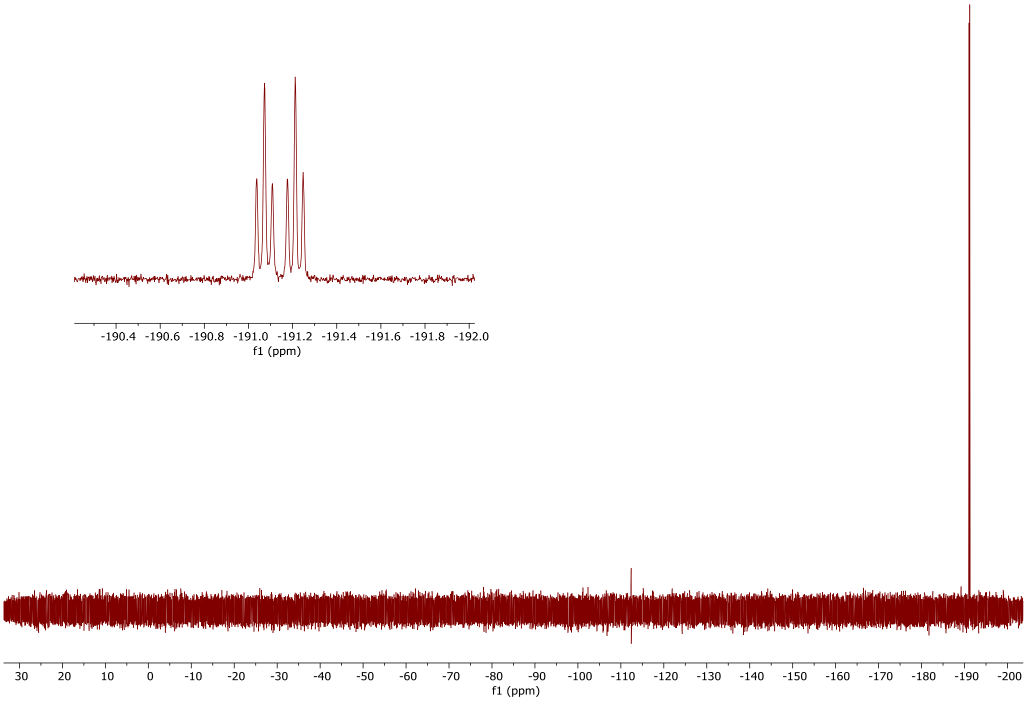

HSQC NMR of S8 ($CDCl_3$)

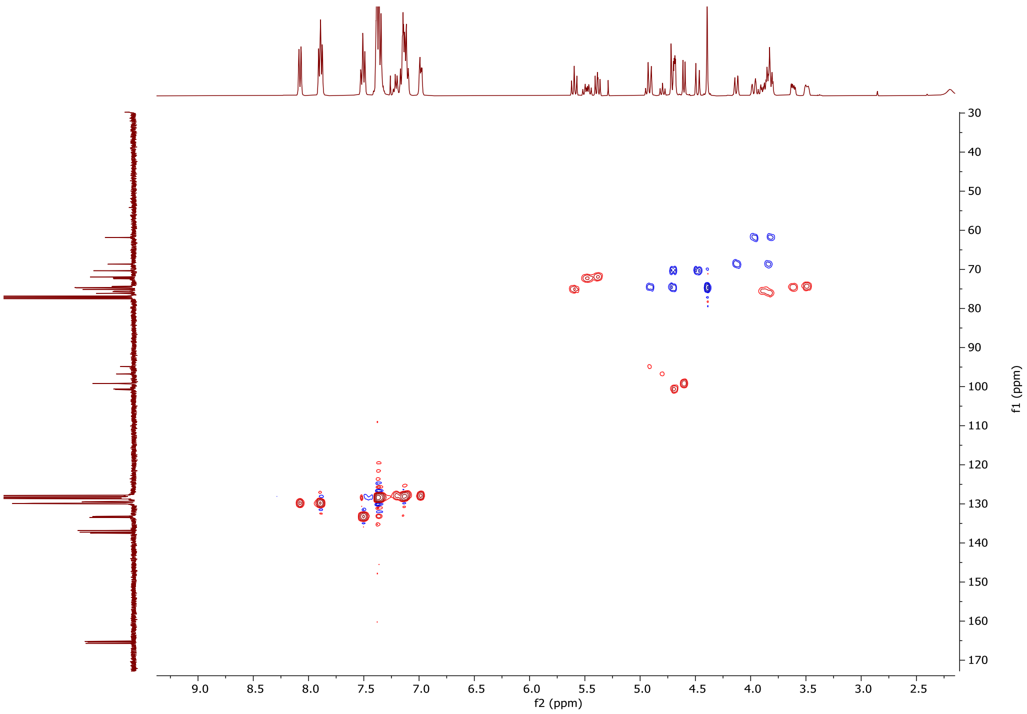

*Synthesis of **4***

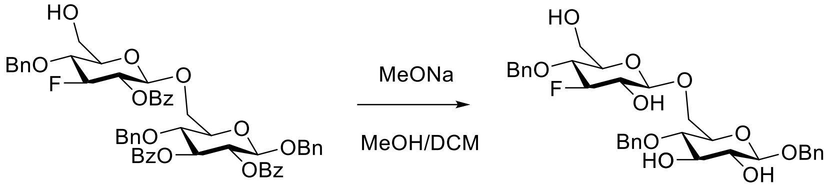

S8 **4**

S8 (52 mg, 0.051 mmol) was dissolved in a 1:1 mixture of MeOH:DCM (4 mL). MeONa in MeOH (0.5 M, 3 equiv. per benzoyl ester) was added and the solution was stirred at RT for 16 h, neutralized with Amberlite IR-120 (H^+ form) resin, filtered, and concentrated *in vacuo*. The resulting yellow oil was purified by column chromatography (DCM: MeOH = 15:1) and recrystallized from DCM:Hex to give **4** as white solid (31 mg, 99%). ^{1}H NMR (400 MHz, CD_3OD) δ 7.51 – 7.16 (m, 15H), 5.01 – 4.89 (m, 3H), 4.75 – 4.61 (m, 3H), 4.38 (d, *J* = 7.8 Hz, 1H), 4.33 (d, *J* = 7.8 Hz, 1H), 4.15 (d, *J* = 10.9 Hz, 1H), 3.88 – 3.72 (m, 2H), 3.72 – 3.62 (m, 1H), 3.62 – 3.45 (m, 4H), 3.41 (t, *J* = 9.3 Hz, 1H), 3.36 – 3.18 (m, 3H). ^{13}C NMR (101 MHz, CD_3OD) δ 140.10, 140.04, 139.10, 129.31, 129.28, 129.16, 129.13, 129.08, 128.69, 128.65, 105.00, 103.42, 79.34, 79.12, 78.51, 78.37, 77.05, 76.17, 75.73, 75.67, 75.37, 75.31, 71.97, 69.56, 62.32. ^{19}F NMR (376 MHz, CD_3OD) δ -191.91 (dt, *J* = 52.8, 14.2 Hz). (ESI-HRMS) m/z 637.2440 [M+Na]$^+$ ($C_{33}H_{40}O_{11}Na$ requires 637.2419).

^{1}H NMR of 4 (400 MHz, CD_3OD)

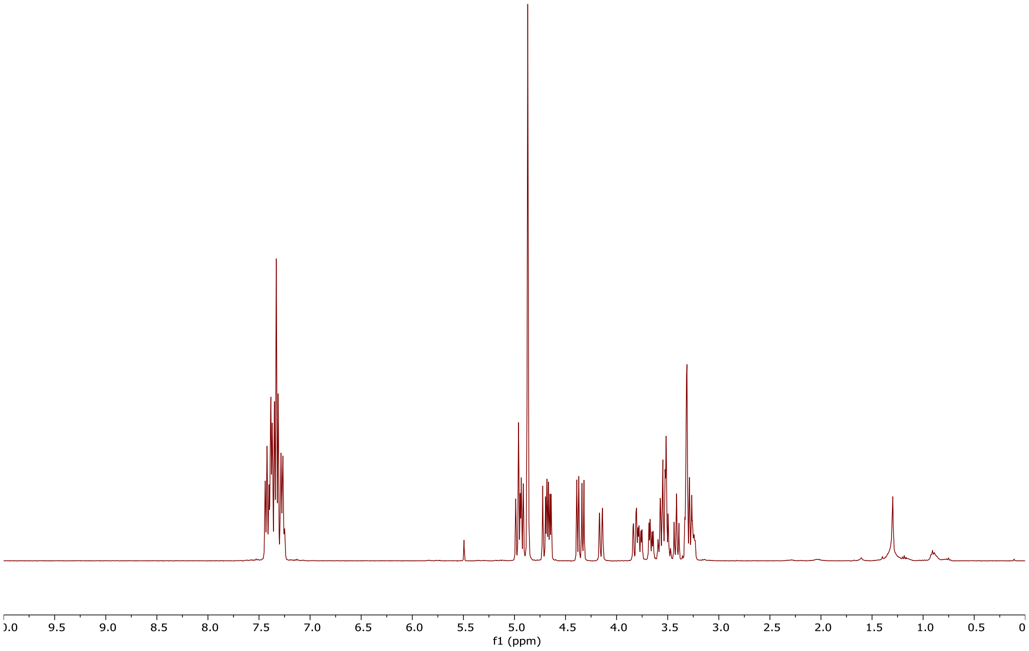

^{13}C NMR of 4 (101 MHz, CD_3OD)

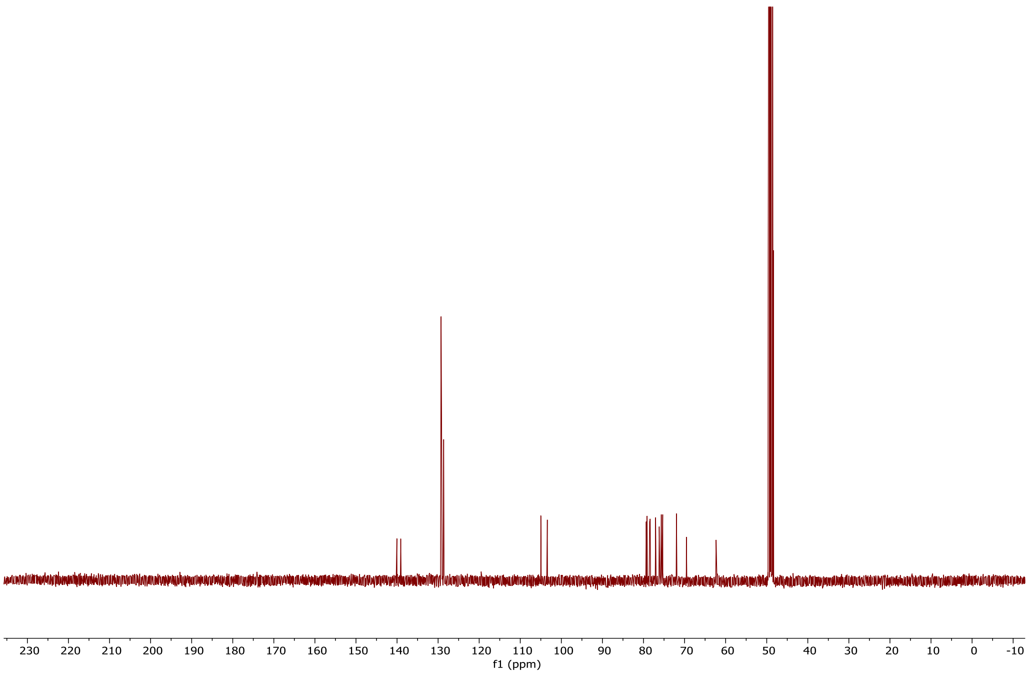

^{19}F NMR of 4 (376 MHz, CD_3OD)

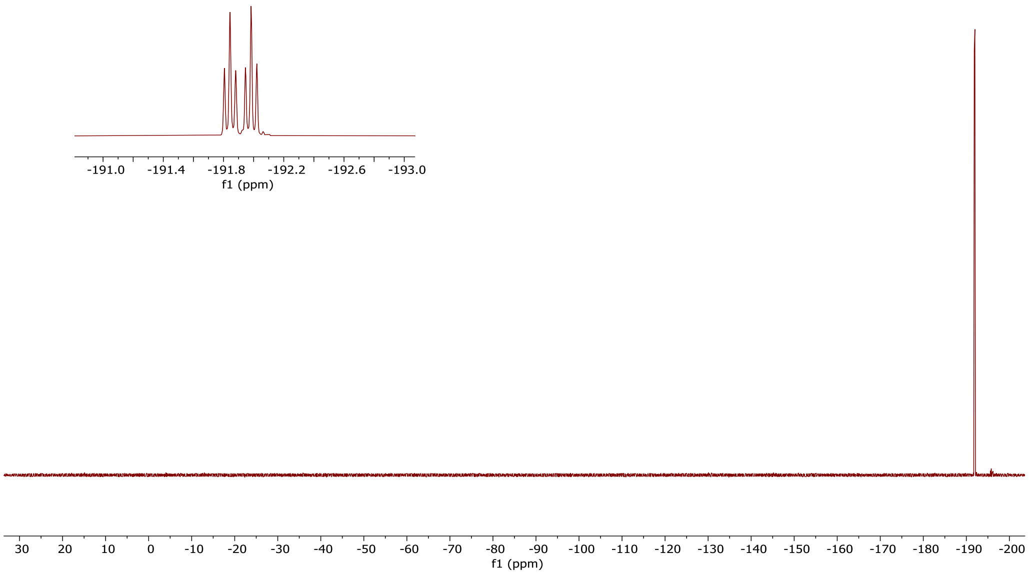

HSQC NMR of 4 (CD_3OD)

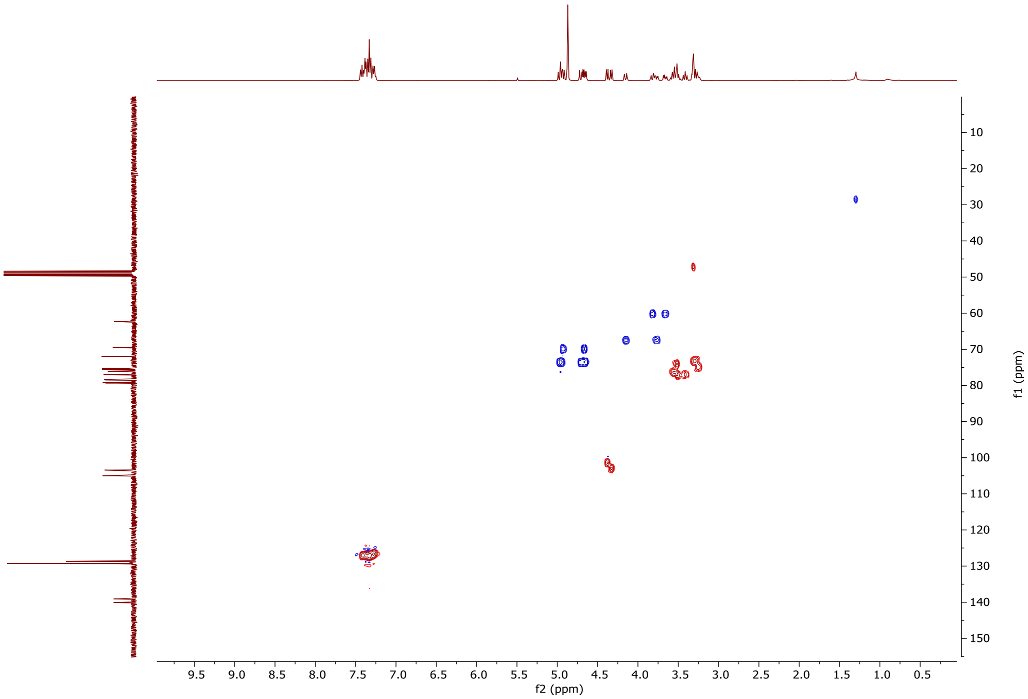

2.4. Synthesis of 5

Synthesis of ***S10***

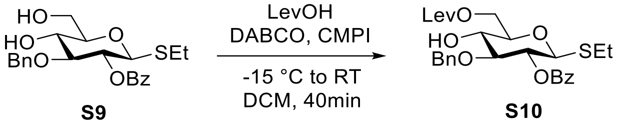

S9 **S10**

S9[6] (7.0 g, 16.75 mmol) was dissolved in DCM (200 mL). Levulinic acid (3.45 mL, 33.5 mmol) and 2-chloro-1-methylpyridinium iodide (8.5 gr, 33.5 mmol) were added. The reaction was stirred for 15 min, then cooled to -15 ^{o}C and DABCO (7.5 g, 67 mmol) was added. The reaction mixture was stirred for 40min and then filtered over a plug of celite and concentrated *in vacuo*. The reaction mixture was quenched with sat. aq. $NaHCO_3$ (200 mL) and extracted with DCM. The organic layer was washed with brine and dried over Na_2SO_4. Removal of the solvent under reduced pressure and purification by flash chromatography (Hexane : EtOAc = 1:1) afforded **S10** as a white solid (7.3 g, 84 %). ^{1}HNMR (400 MHz, $CDCl_3$) δ 8.11 (dt, *J* = 8.1, 1.1 Hz, 2H), 7.70 – 7.63 (m, 1H), 7.57 – 7.49 (m, 2H), 7.26 (s, 5H), 5.39 – 5.29 (m, 1H), 4.78 (q, *J* = 11.4 Hz, 2H), 4.68 – 4.52 (m, 2H), 4.39 (dd, *J* = 12.2, 2.1 Hz, 1H), 3.82 – 3.70 (m, 2H), 2.98 – 2.66 (m, 7H), 2.27 (s, 3H), 1.41 – 1.25 (m, 3H).; ^{13}CNMR (101 MHz, $CDCl_3$) δ 207.00, 173.47, 165.38, 137.91, 133.42, 130.00, 129.85, 128.59, 128.18, 128.03, 83.97, 83.35, 78.07, 74.95, 72.15, 70.15, 63.50, 38.14, 30.03, 28.04, 24.30, 14.98.; m/z (HRMS+) 539.1716 $[M+Na]^+$ ($C_{27}H_{32}O_6SNa$ requires 539.1716) $[\alpha]_D^{20}$ = + 35.51 ; IR (neat) ν_{max}= 2930.2, 1721.1, 1361.4, 1273.8, 1070.6,749.7, 713.5.; R_f = 0.5 (silica, Hex : EtOAc 1 : 1).

^{1}H NMR of S10 (400 MHz, $CDCl_3$)

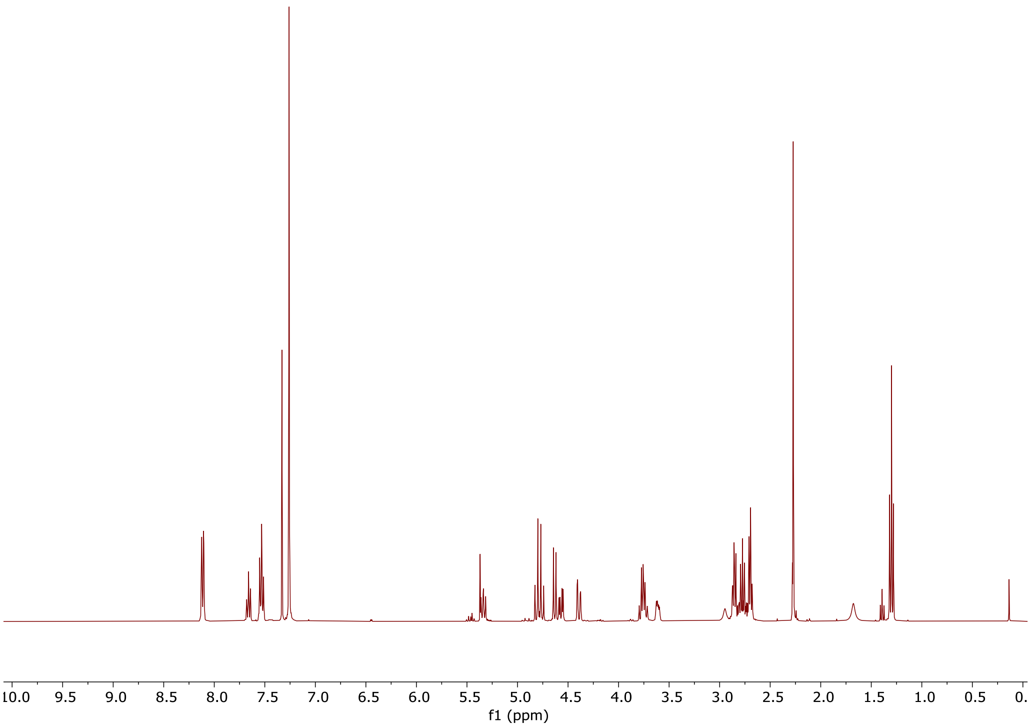

^{13}C NMR of S10 (101 MHz, $CDCl_3$)

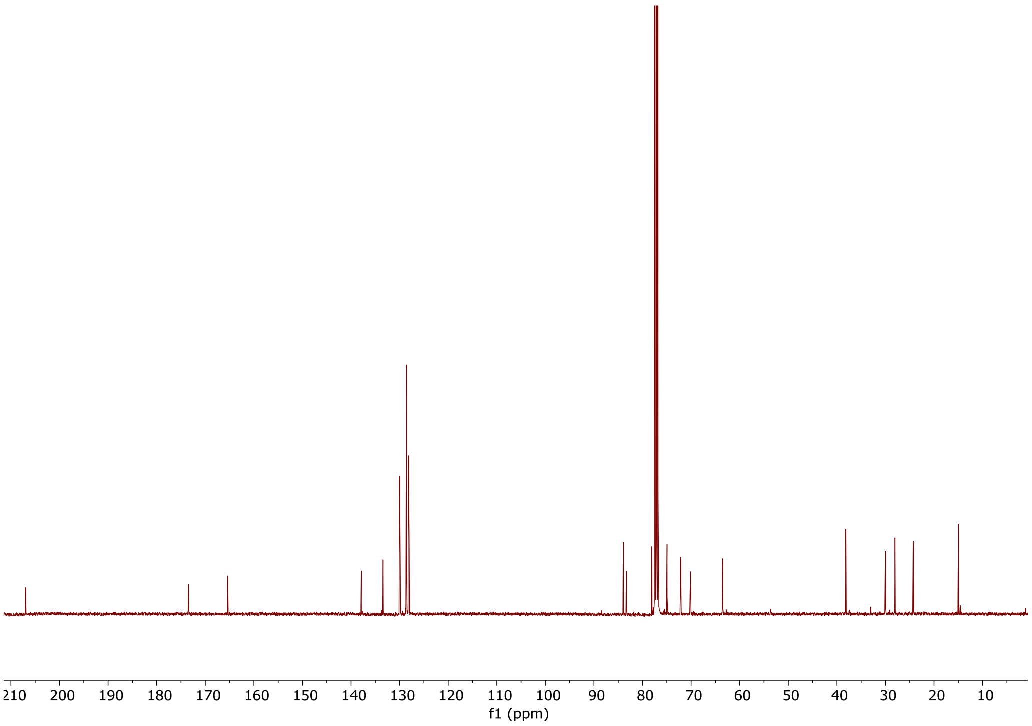

HSQC NMR of S10 ($CDCl_3$)

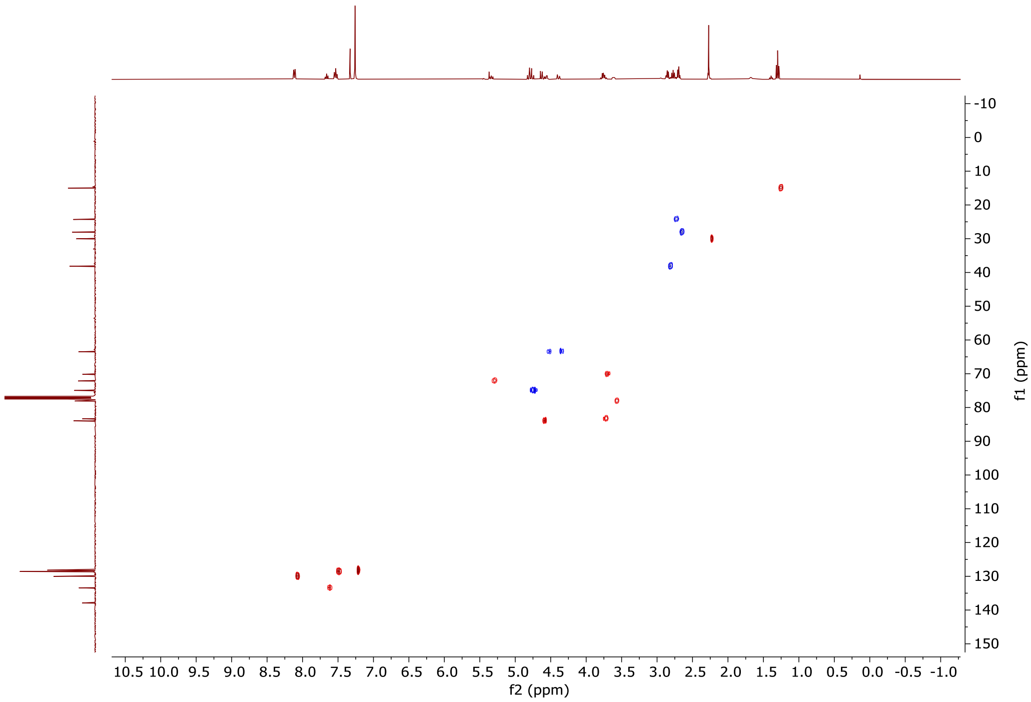

*Synthesis of **S11***

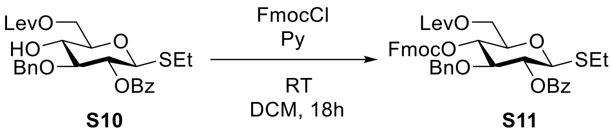

S10 (7.3 g, 14.14 mmol) was dissolved in DCM (100 mL) and pyridine was added (3.5 mL, 42.4 mmol). FmocCl (7.3 gr, 28.28 mmol) was dissolved in DCM (100 mL) and added to the reaction mixture. The yellow solution was stirred for 3h and then quenched with 1M solution of HCl. The organic layer was washed one time with 1M HCl, one time with sat. aq. solution of $NaHCO_3$ and one time with brine. The crude compound was purified with flash column chromatography (Hexane : EtOAc = 2:1) to give **S11** as white solid (6.7 g, 68.2 %) . ^{1}HNMR (400 MHz, $CDCl_3$) δ 8.29 – 8.19 (m, 2H), 7.97 (ddt, J = 7.2, 6.3, 0.9 Hz, 2H), 7.87 – 7.78 (m, 3H), 7.68 (t, J = 7.8 Hz, 2H), 7.65 – 7.57 (m, 2H), 7.55 – 7.47 (m, 2H), 7.33 – 7.22 (m, 5H), 5.57 (dd, J = 10.0, 9.1 Hz, 1H), 5.22 (dd, J = 10.1, 9.3 Hz, 1H), 4.85 – 4.66 (m, 4H), 4.61 (dd, J = 10.5, 7.2 Hz, 1H), 4.53 – 4.40 (m, 3H), 4.12 (t, J = 9.2 Hz, 1H), 3.97 (ddd, J = 10.1, 4.4, 3.4 Hz, 1H), 3.06 – 2.89 (m, 4H), 2.89 – 2.76 (m, 2H), 2.40 (s, 3H), 1.45 (d, J = 7.4 Hz, 3H); ^{13}CNMR (101 MHz, $CDCl_3$) δ 206.54, 172.40, 165.02, 154.20, 143.32, 143.07, 141.34, 141.31, 137.20, 133.38, 129.92, 129.57, 128.49, 128.20, 127.98, 127.96, 127.88, 127.72, 127.27, 127.25, 125.14, 124.99, 120.14, 120.12, 83.84, 80.92, 75.83, 74.49, 74.44, 71.79, 70.23, 62.71, 46.74, 37.90, 29.91, 27.90, 24.18, 14.89.; m/z (HRMS+) 761.2300 $[M+Na]^+$ ($C_{42}H_{42}O_{10}SNa$ requires 761.2396) $[\alpha]_D^{20}$ = +29.48 ; IR (neat) ν_{max}= 3661, 2982, 1463.5, 1383.2, 1252.5, 1153, 1073, 955, 816.5.; R_f = 0.5 (silica, Hex : EtOAc 2 : 1).

^{1}H NMR of S11 (400 MHz, $CDCl_3$)

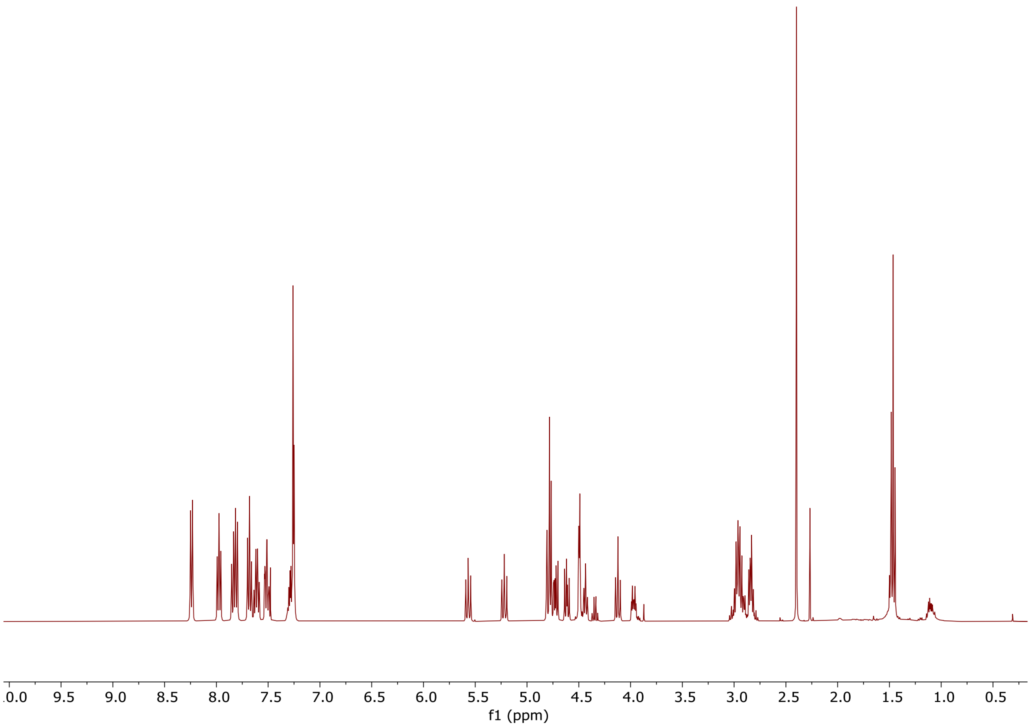

^{13}C NMR of S11 (101 MHz, $CDCl_3$)

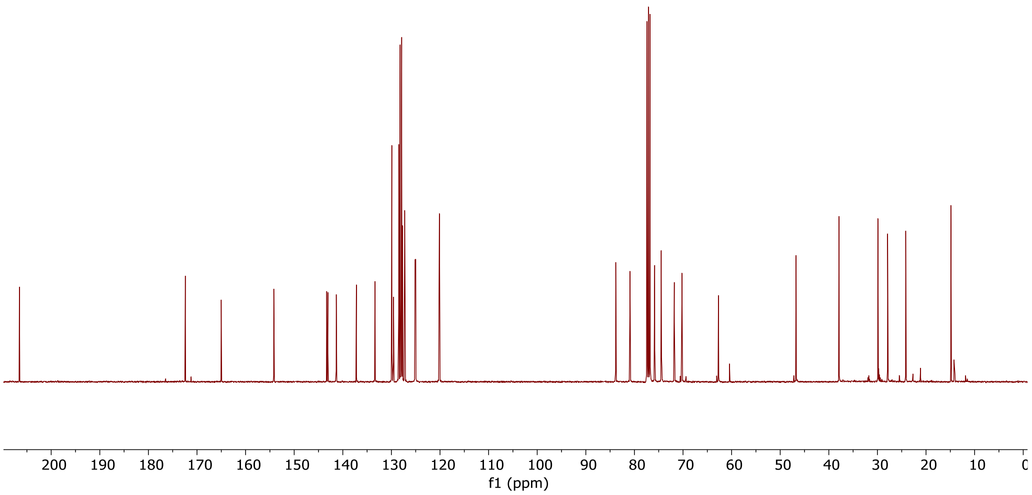

HSQC NMR of S11 ($CDCl_3$)

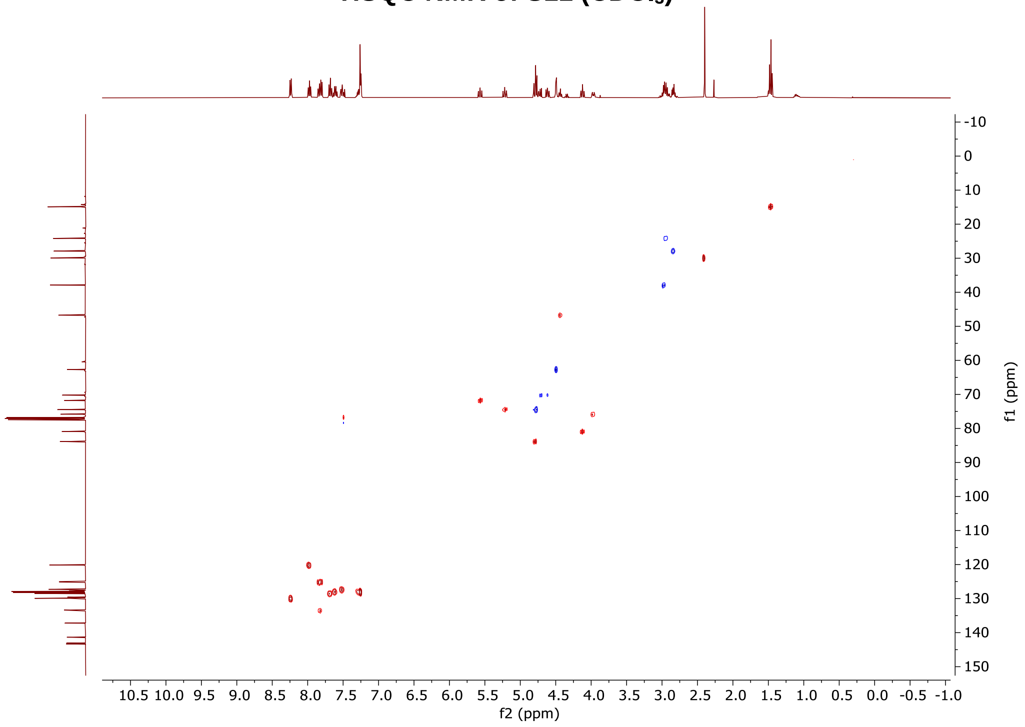

Synthesis of ***S12***

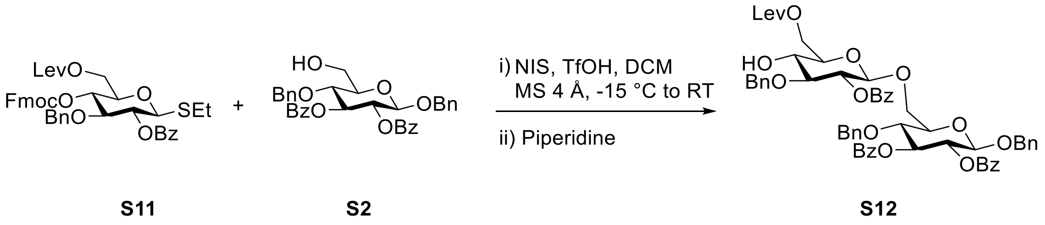

S11 (24.8 mg, 0.044 mmol), **S2** (30.0, 0.048 mmol), and *N*-iodosuccinimide (53.5 mg, 0.023.9 mmol) were dissolved in anhydrous DCM (20 mL). The solution was then stirred with molecular sieve for 1 h at RT under N_2 atmosphere and then cooled to -15 °C. A 1% solution of TfOH in DCM (200 µL, v/v) was added and the reaction was stirred for 30 min at -15 °C before the removal of the cooling bath to allow the reaction to RT. After TLC indicated the disappearance of **S11**, piperidine (0.5 mL) was added and the yellow solution was stirred at RT for additional 30 min. The reaction was diluted with DCM and washed with H_2O and brine. The organic layer was dried over Na_2SO_4, filtered, and evaporated. The resulting yellow oil was purified by column chromatography (hexane: EtOAc = 3:1) to give **S12** as pale oil (35.2 mg, 88%). ^{1}H NMR (400 MHz, $CDCl_3$) δ 8.08 – 8.01 (m, 2H), 7.89 (dt, *J* = 8.4, 1.3 Hz, 4H), 7.57 – 7.46 (m, 3H), 7.42 – 7.31 (m, 6H), 7.24 – 7.09 (m, 13H), 7.03 – 6.96 (m, 2H), 5.59 (dd, *J* = 9.8, 9.0 Hz, 1H), 5.38 (td, *J* = 9.6, 7.9 Hz, 2H), 4.83 – 4.72 (m, 2H), 4.72 – 4.64 (m, 2H), 4.59 (dd, *J* = 7.9, 5.2 Hz, 2H), 4.46 (d, *J* = 12.7 Hz, 1H), 4.42 – 4.30 (m, 3H), 4.24 (dd, *J* = 10.8, 1.6 Hz, 1H), 3.82 – 3.74 (m, 3H), 3.74 – 3.61 (m, 2H), 3.55 (ddd, *J* = 9.5, 4.2, 2.2 Hz, 1H), 2.84 – 2.78 (m, 2H), 2.64 (dd, *J* = 7.6, 6.1 Hz, 2H): ^{13}C NMR (101 MHz, $CDCl_3$) δ 207.10, 177.25, 173.51, 165.58, 165.27, 165.12, 137.92, 137.86, 137.15, 136.87, 133.25, 133.17, 133.09, 129.87, 129.79, 129.75, 129.71, 129.41, 129.35, 129.07, 128.51, 128.46, 128.36, 128.29, 128.26, 128.09, 127.89, 127.81, 127.76, 127.71, 125.33, 101.24, 99.01, 81.75, 76.23, 75.08, 74.61, 74.56, 74.53, 74.04, 73.31, 71.92, 70.02, 69.99, 67.80, 63.09, 38.03, 31.97, 29.92, 29.74, 29.59, 27.95, 22.74, 21.51. (ESI-HRMS) m/z 1045.397 $[M+Na]^+$ ($C_{59}H_{58}O_{16}Na$ requires 1045.362).

^{1}H NMR of S12 (400 MHz, $CDCl_3$)

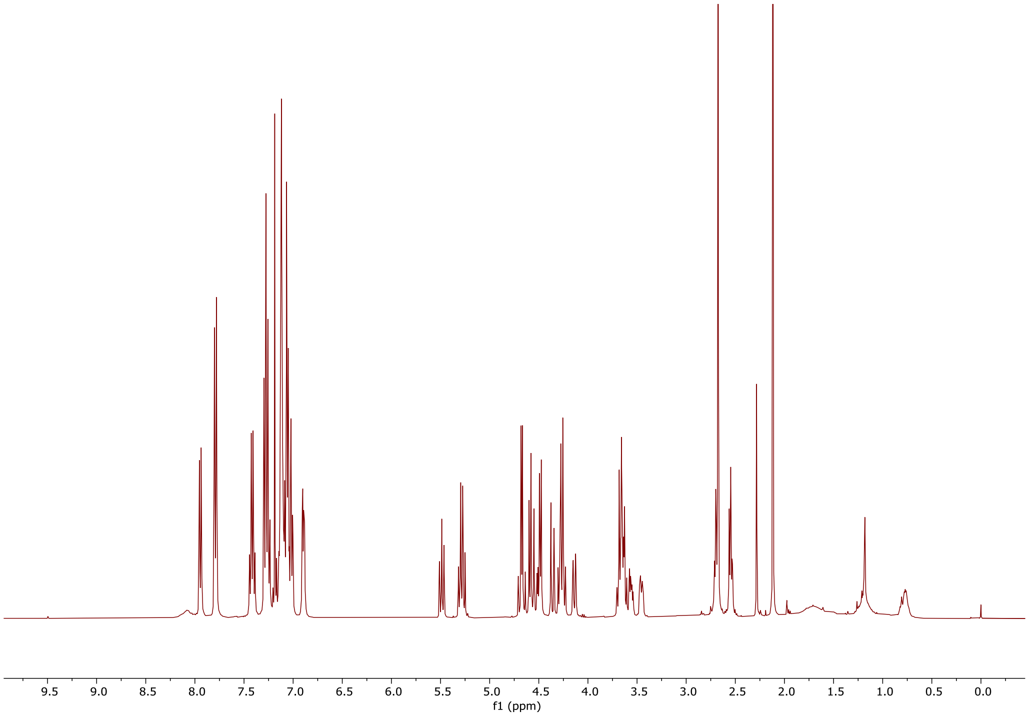

^{13}C NMR of S12 (101 MHz, $CDCl_3$)

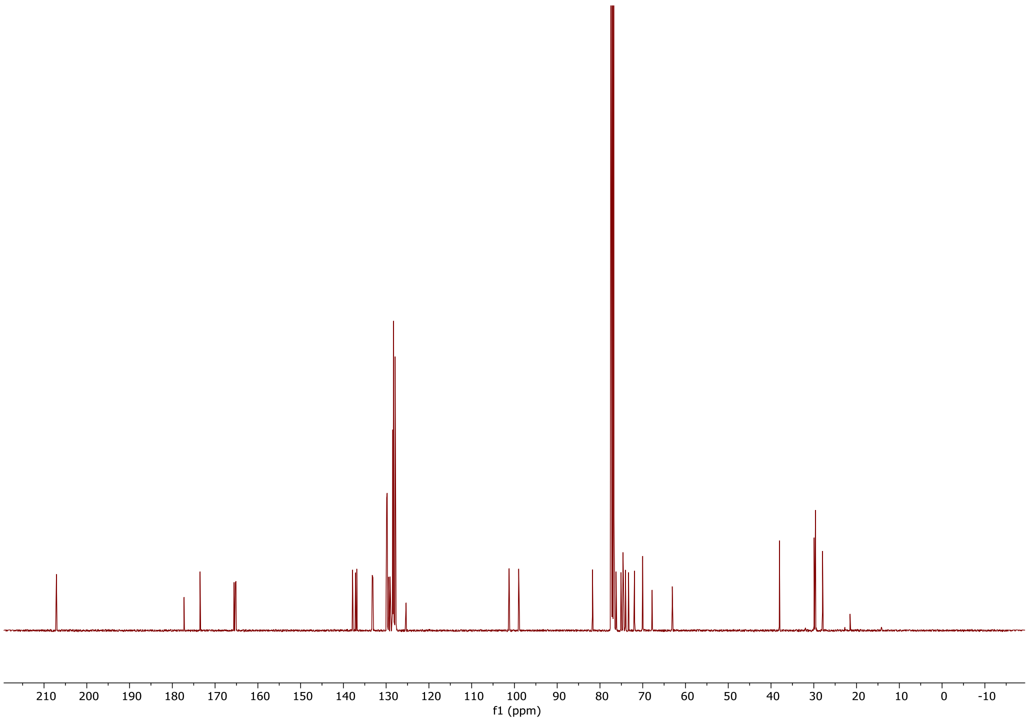

HSQC NMR of S12 ($CDCl_3$)

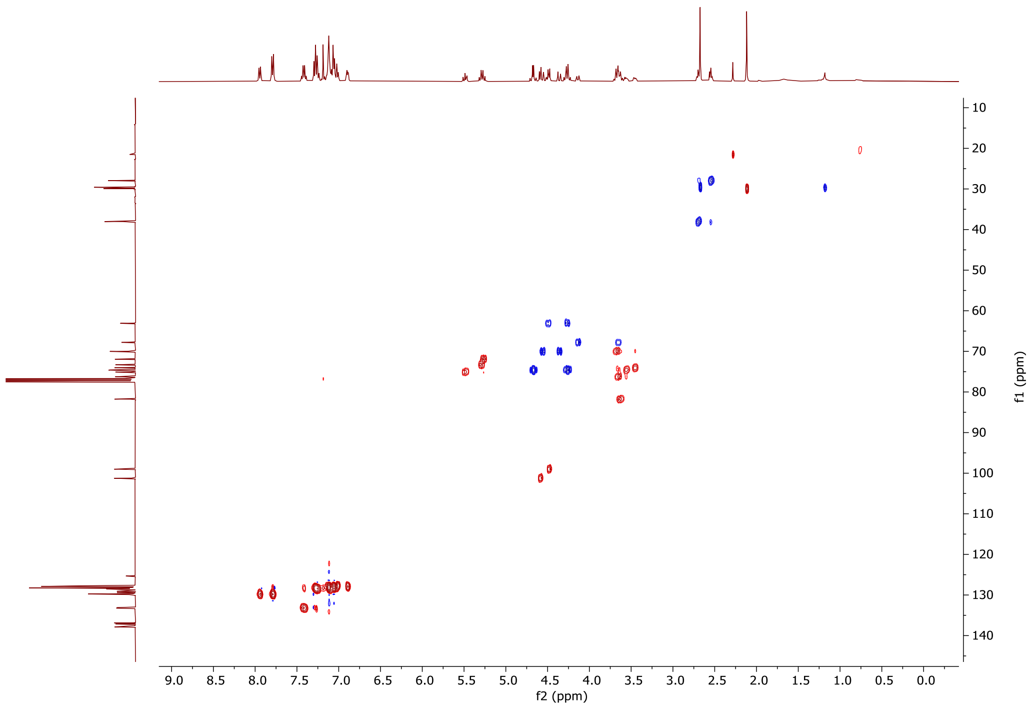

Synthesis of ***5***

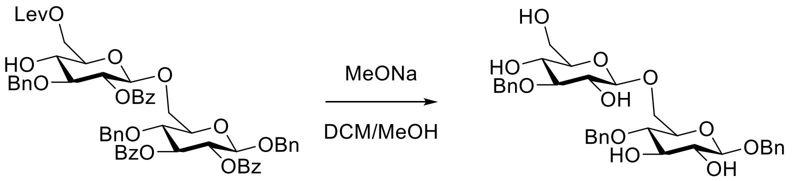

S12 **5**

Compound **S12** (35.2 mg, 0.039 mmol) was dissolved in a 1:1 mixture of DCM:MeOH (4 mL). A solution of NaOMe (0.5 mL, 0.5 M in MeOH) was added. The solution was stirred overnight at RT and then quenched with Amberlite IR-120 (H^+ form) resin, filtered, and concentrated. The crude compound was purified with flash column chromatography (DCM:MeOH = 20:1) to give **5** as white solid (15.2 mg, 64 %). ^1H NMR (400 MHz, $CDCl_3$) δ 7.29 – 7.18 (m, 15H), 4.95 – 4.79 (m, 3H), 4.63 (t, *J* = 11.7 Hz, 2H), 4.48 (d, *J* = 11.6 Hz, 1H), 4.27 (dd, *J* = 9.7, 7.8 Hz, 2H), 4.11 – 4.02 (m, 1H), 3.75 – 3.57 (m, 5H), 3.56 – 3.38 (m, 4H), 3.29 (t, *J* = 9.0 Hz, 1H), 3.19 (dd, *J* = 9.7, 3.8 Hz, 1H); ^{13}C NMR (101 MHz, $CDCl_3$) δ 138.50, 138.18, 136.83, 128.58, 128.53, 128.18, 128.09, 128.02, 127.95, 103.73, 101.62, 83.72, 77.48, 77.26, 75.40, 74.76, 74.60, 73.99, 73.87, 71.36, 69.48, 68.27, 62.12, 60.48. (ESI-HRMS) m/z 635.2485 $[M+Na]^+$ ($C_{33}H_{40}O_{11}Na$ requires 635.2463).

^{1}H NMR of 5 (400 MHz, $CDCl_3$)

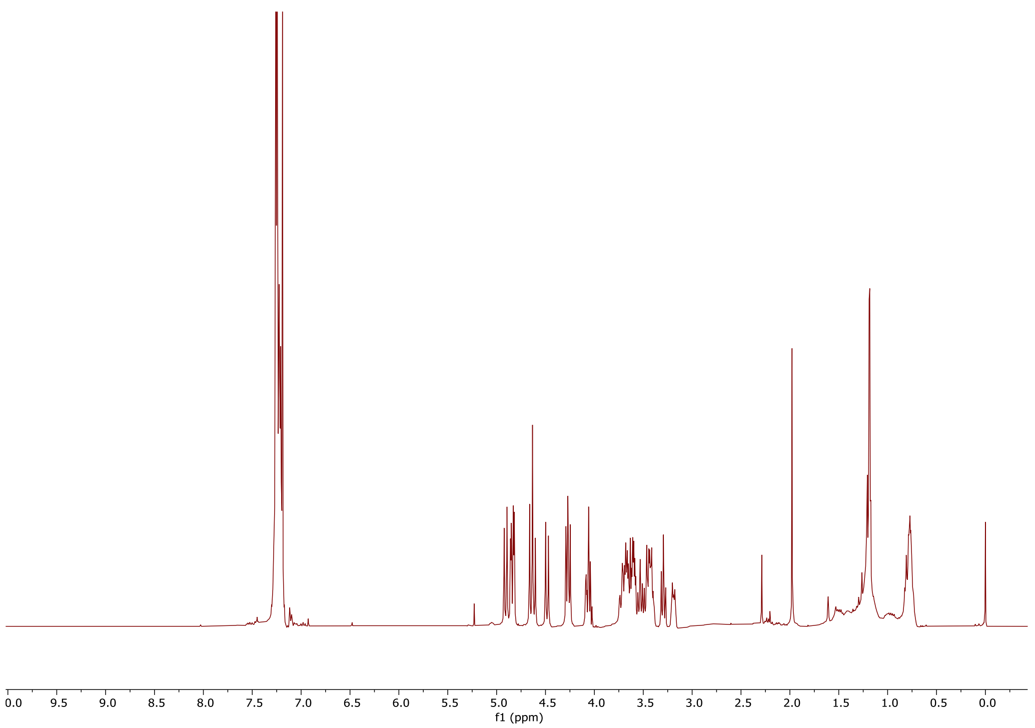

^{13}C NMR of 5 (101 MHz, $CDCl_3$)

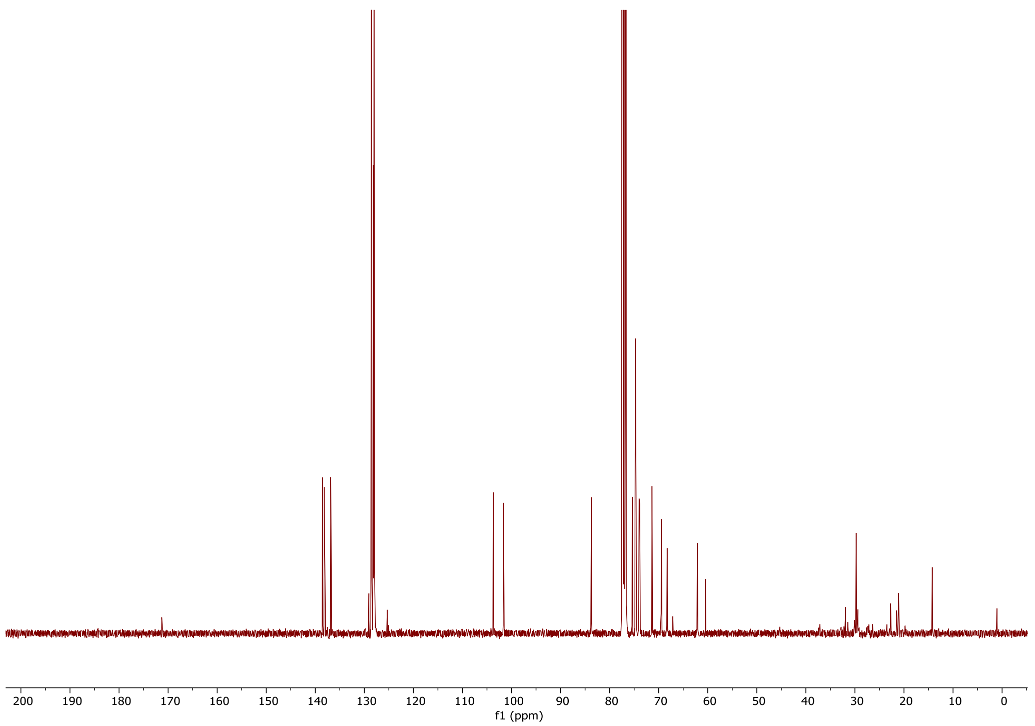

HSQC NMR of 5 ($CDCl_3$)

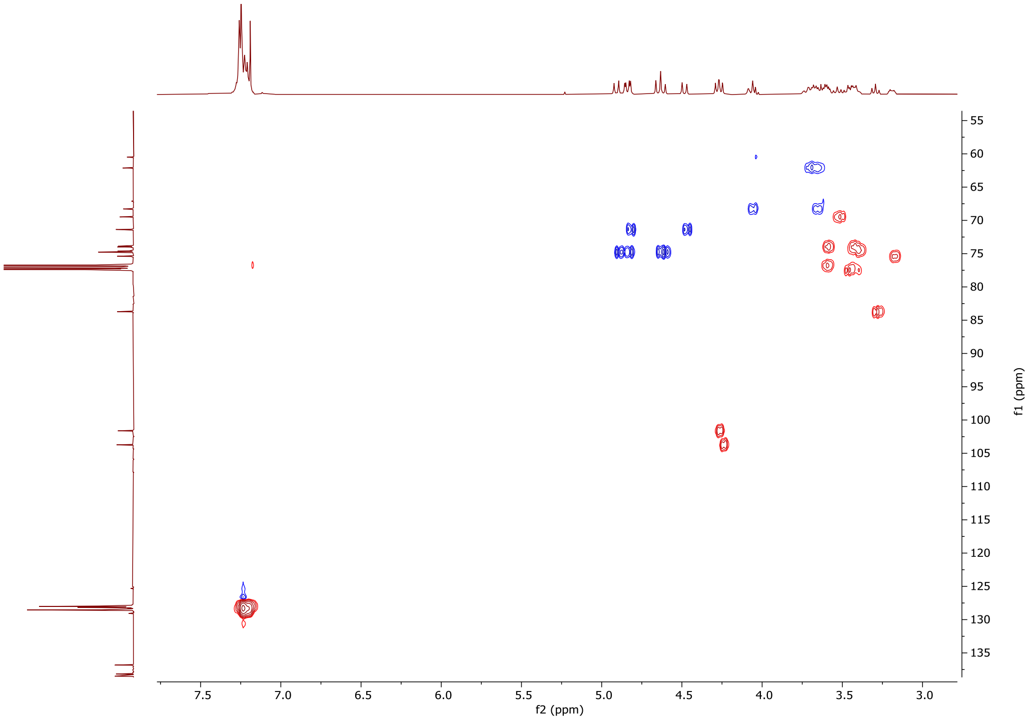

2.5. Synthesis of 6

*Synthesis of **S14***

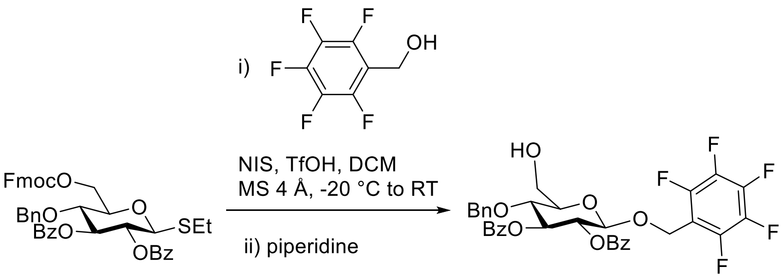

S13 **S14**

Ethyl 2,3-di-*O*-benzoyl-4-*O*-benzyl-6-*O*-(9-fluorenylmethoxycarbonyl)-1-thio-β-D-glucopyranoside, **S13**[7] (70 mg, 0.094 mmol) and 2,3,4,5,6-pentafluorobenzyl alcohol (40 mg, 0.20 mmol) were dissolved in anhydrous DCM (1 mL). The solution was stirred with molecular sieves (4 Å) for 1 h at RT under Ar atmosphere and then cooled to -20 °C. The activator solution (250 µL of a 200 mg/mL solution of NIS in DCM:dioxane 2:1 with 1% TfOH) was added dropwise and the reaction was stirred for 5 min at -20 °C, after which time the cooling bath was removed to allow the reaction to RT. After 1 h at RT, ESI-MS indicated the disappearance of **S13**. Piperidine (0.5 mL) was added and the reaction was stirred at RT for additional 1 h. The reaction was diluted with DCM and washed once with 1 M HCl, once with $NaHCO_3$ sat. aq. solution and once with brine. The crude was purified by column chromatography (Hexane:EtOAc = 3:1→2:1→1:1) to give **S14** as a white foam (53 mg, 81%). ^{1}H NMR (400 MHz, $CDCl_3$) δ 7.95 – 7.89 (m, 2H), 7.86 – 7.79 (m, 2H), 7.55 – 7.46 (m, 2H), 7.40 – 7.31 (m, 4H), 7.21 – 7.12 (m, 5H), 5.71 (t, *J* = 9.6 Hz, 1H), 5.35 – 5.26 (m, 1H), 4.93 (d, *J* = 12.0 Hz, 1H), 4.78 (d, *J* = 8.0 Hz, 1H), 4.75 (d, *J* = 12.0 Hz, 1H), 4.01 – 3.92 (m, 2H),

3.85 (dd, J = 12.2, 3.6 Hz, 1H), 3.63 (dt, J = 9.6, 3.0 Hz, 1H), 1.84 (s, 1H). ^{13}C NMR (101 MHz, $CDCl_3$) δ 165.77, 165.22, 137.22, 133.39, 133.36, 129.89, 129.62, 129.41, 129.18, 128.54, 128.40, 128.34, 128.16, 101.17, 75.92, 75.32, 74.99, 74.83, 71.87, 61.54, 58.92. ^{19}F NMR (376 MHz, $CDCl_3$) δ -142.48 (dd, J = 22.2, 8.5 Hz, 2F), -152.63 (t, J = 20.8 Hz, 1F), -161.49 (td, J = 21.7, 8.5 Hz, 2F). $[\alpha]_D^{20}$ 11.41 (c 1.06 g/100 mL, $CHCl_3$). IR v = 2927, 1729, 1507, 1273, 1096, 1071 cm^{-1}. (ESI-HRMS) m/z 681.1479 $[M+Na]^+$ ($C_{34}H_{27}F_5O_8Na$ requires 681.1518).

^{1}H NMR of S14 (400 MHz, $CDCl_3$)

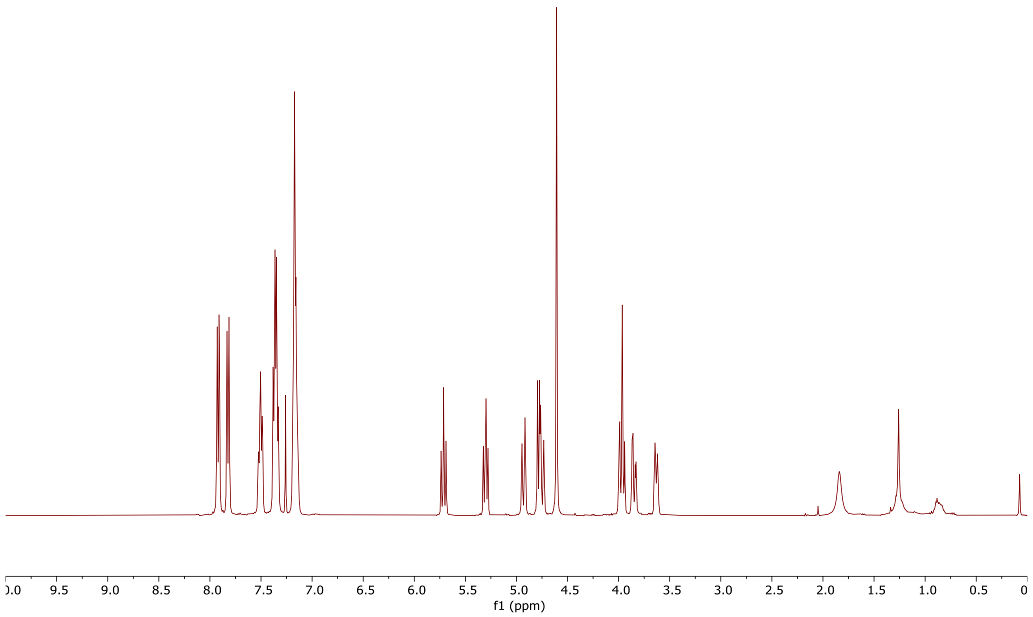

^{13}C NMR of S14 (101 MHz, $CDCl_3$)

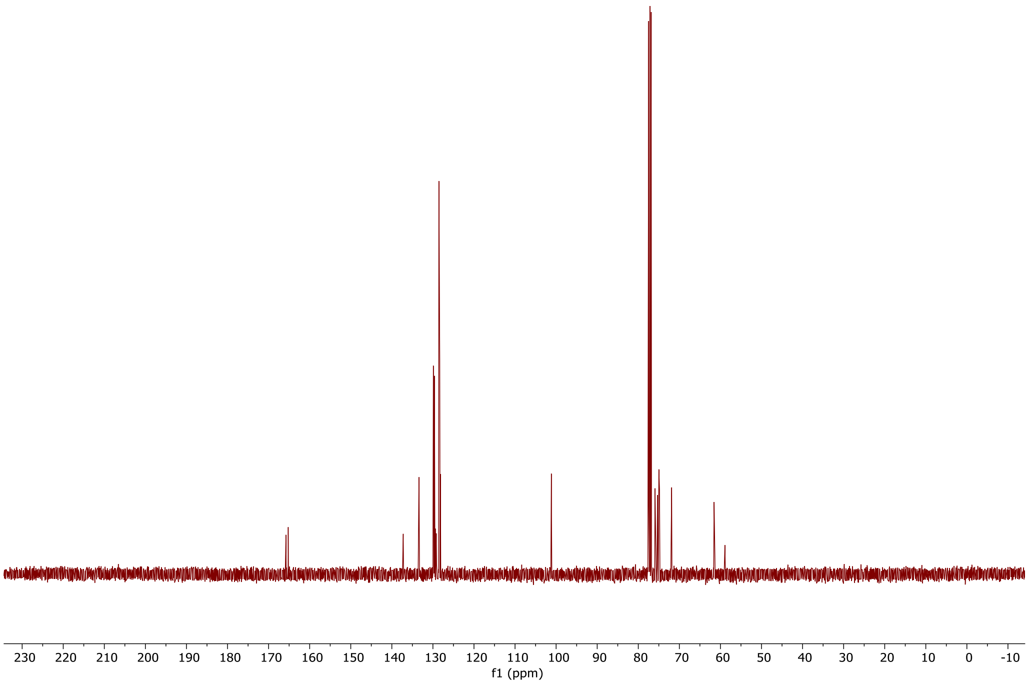

^{19}F NMR of S14 (376 MHz, $CDCl_3$)

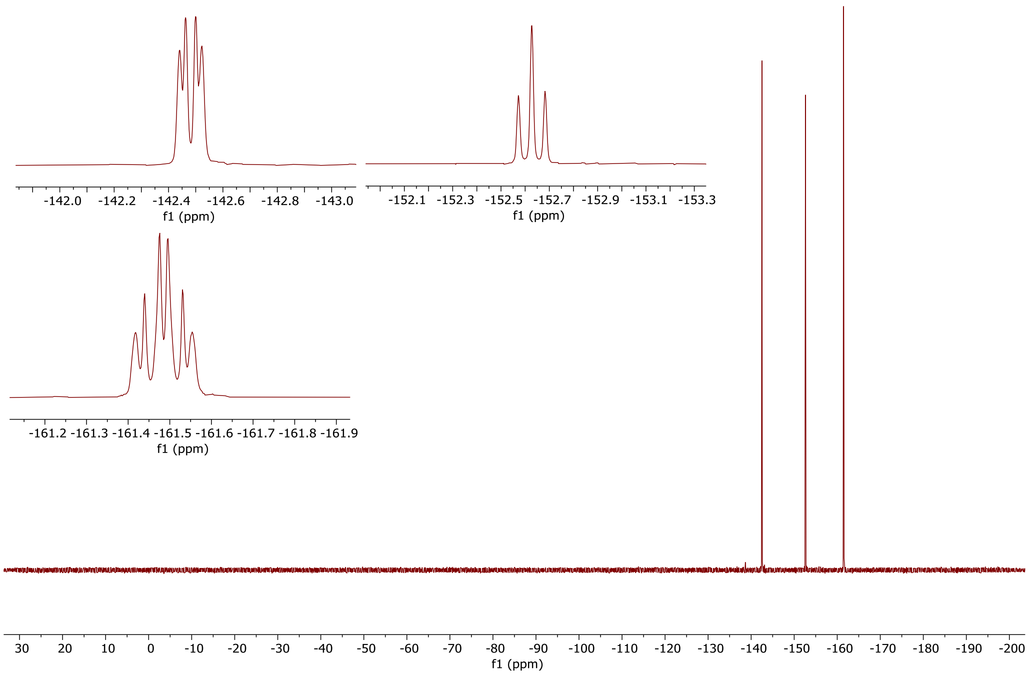

HSQC NMR of S14 ($CDCl_3$)

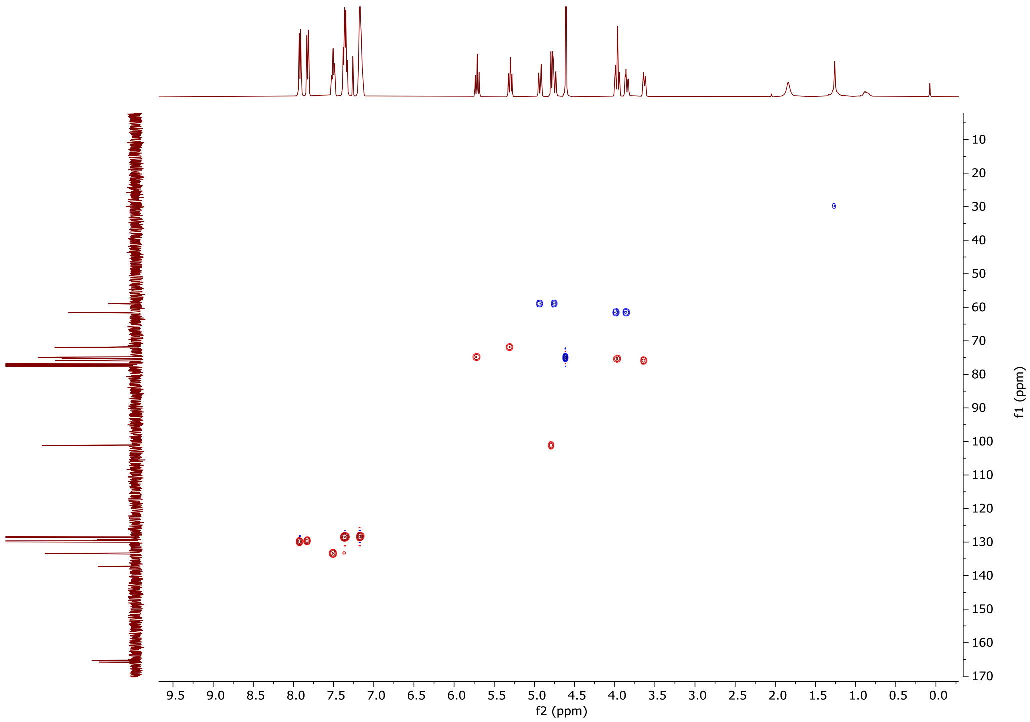

Synthesis of ***S15***

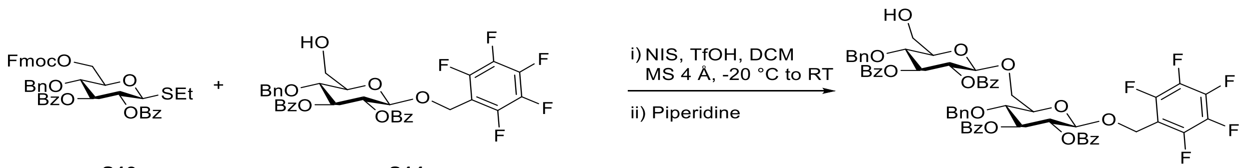

S13 **S14** **S15**

S14 (53 mg, 0.080 mmol) and **S13** (72 mg, 0.097 mmol) were dissolved in anhydrous DCM (1 mL). The solution was stirred with molecular sieves (4 Å) for 1 h at RT under Ar atmosphere and then cooled to -20 °C. The activator solution (250 µL of a 200 mg/mL solution of NIS in DCM:dioxane 2:1 with 1% TfOH) was added dropwise and the reaction was stirred for 5 min at -20 °C, after which time the cooling bath was removed to allow the reaction to RT. After 1 h at RT, ESI-MS indicated the disappearance of **S14**. Piperidine (0.5 mL) was added and the reaction was stirred at RT for additional 1 h. The reaction was diluted with DCM and washed once with 1M HCl, once with $NaHCO_3$ sat. aq. solution and once with brine. The crude product was purified by column chromatography (Hexane:Acetone = 4:1→3:1→2:1) to give **S15** as a white solid (68 mg, 76 %). ^{1}H NMR (600 MHz, $CDCl_3$) δ 7.95 – 7.89 (m, 4H), 7.88 – 7.83 (m, 2H), 7.82 – 7.77 (m, 2H), 7.54 – 7.46 (m, 3H), 7.41 – 7.30 (m, 7H), 7.25 – 7.21 (m, 2H), 7.21 – 7.10 (m, 8H), 6.99 – 6.94 (m, 2H), 5.76 (t, J = 9.5 Hz, 1H), 5.61 (t, J = 9.4 Hz, 1H), 5.45 (dd, J = 9.8, 7.8 Hz, 1H), 5.24 (dd, J = 9.8, 7.8 Hz, 1H), 4.78 (d, J = 7.8 Hz, 1H), 4.75 (d, J = 12.0 Hz, 1H), 4.66 – 4.61 (m, 3H), 4.52 (dt, J = 12.1, 1.4 Hz, 1H), 4.34 (s, 2H), 4.16 (dd, J = 11.2, 1.7 Hz, 1H), 4.02 – 3.96 (m, 2H), 3.89 – 3.78 (m, 3H), 3.67 (ddd, J = 9.8, 4.8, 1.7 Hz, 1H), 3.63 (ddd, J = 9.6, 4.2, 2.6 Hz, 1H), 1.88 (s, 1H). ^{13}C NMR (151 MHz, $CDCl_3$) δ 165.71, 165.51, 165.16, 165.00, 137.17, 137.12, 129.71, 129.66, 129.44, 129.33, 129.25, 129.11, 128.40, 128.33, 128.31, 128.25, 128.20, 127.99, 127.82, 127.80, 101.28, 100.62, 75.78, 75.62, 75.53, 74.93, 74.91, 74.82, 74.79, 74.68, 72.21, 71.70, 68.24, 61.64, 58.16. ^{19}F NMR (564 MHz, $CDCl_3$) δ -142.26 (dd, J = 22.6, 8.6

Hz, 2F), -153.08 (t, *J* = 20.8 Hz, 1F), -161.70 (td, *J* = 22.0, 8.5 Hz, 2F). (ESI-HRMS) m/z 1141.276 $[M+Na]^+$ ($C_{61}H_{51}F_5O_{15}Na$ requires 1141.304).

^{1}H NMR of S15 (600 MHz, CDCl$_3$)

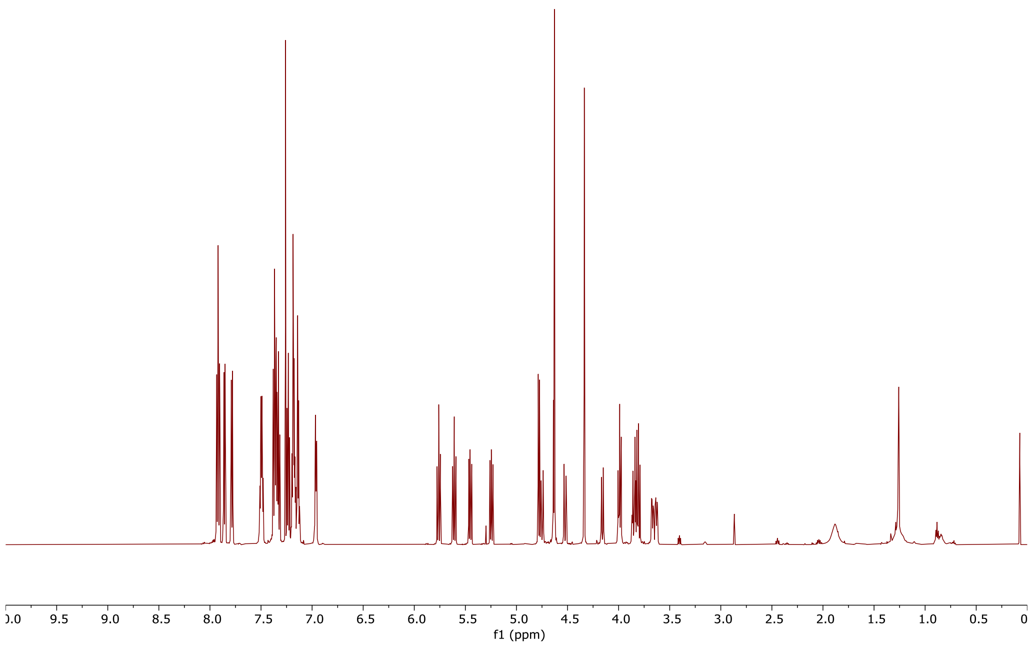

^{13}C NMR of S15 (151 MHz, CDCl$_3$)

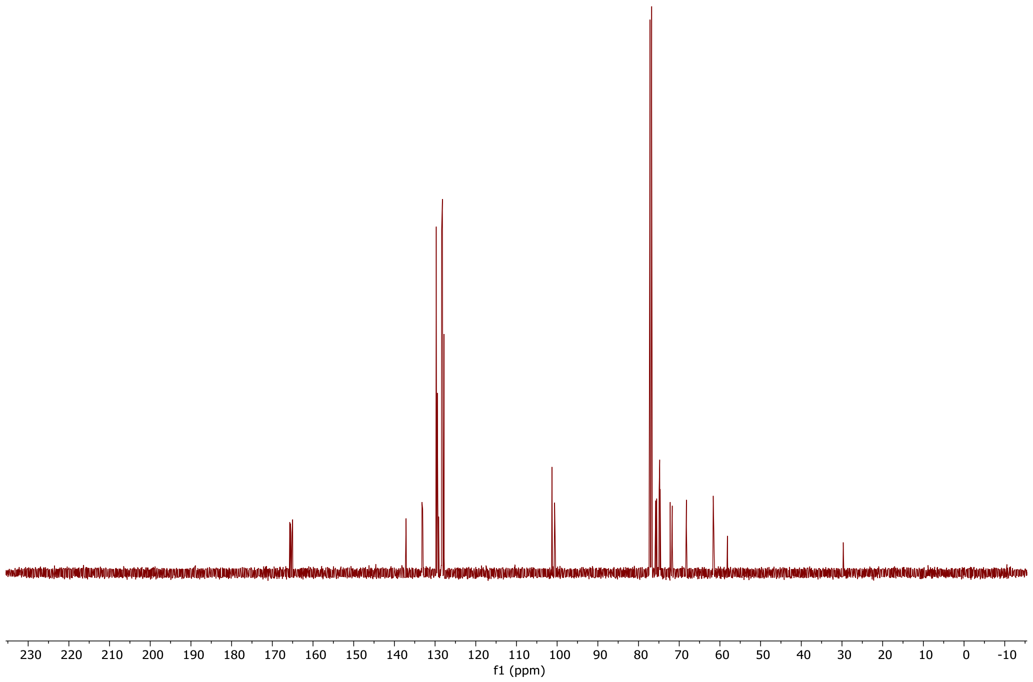

^{19}F NMR of S15 (564 MHz, $CDCl_3$)

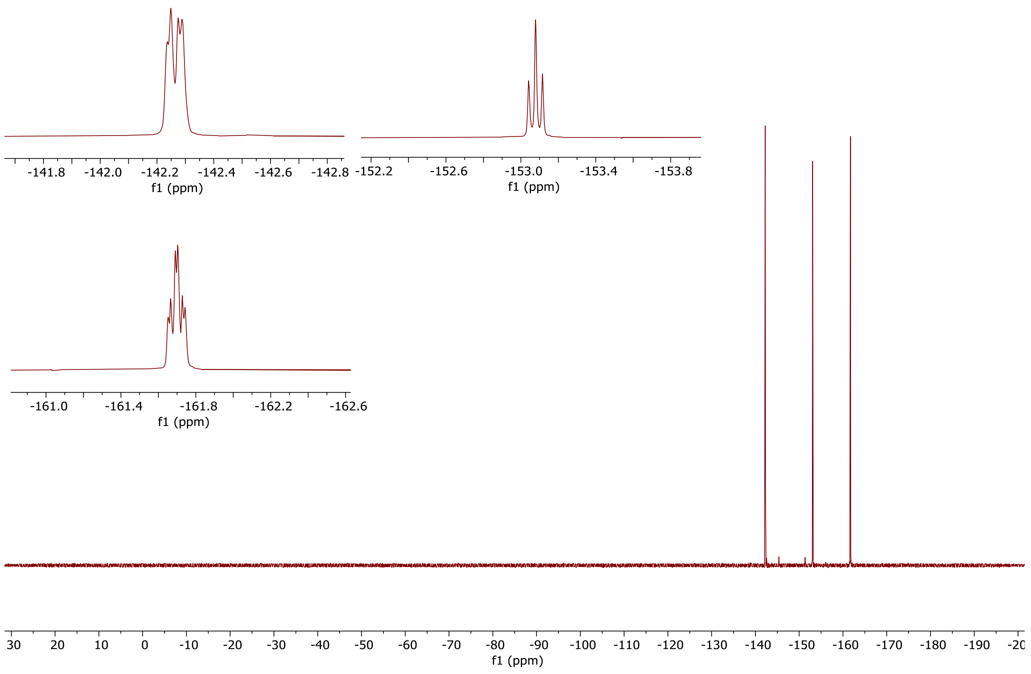

HSQC NMR of S15 ($CDCl_3$)

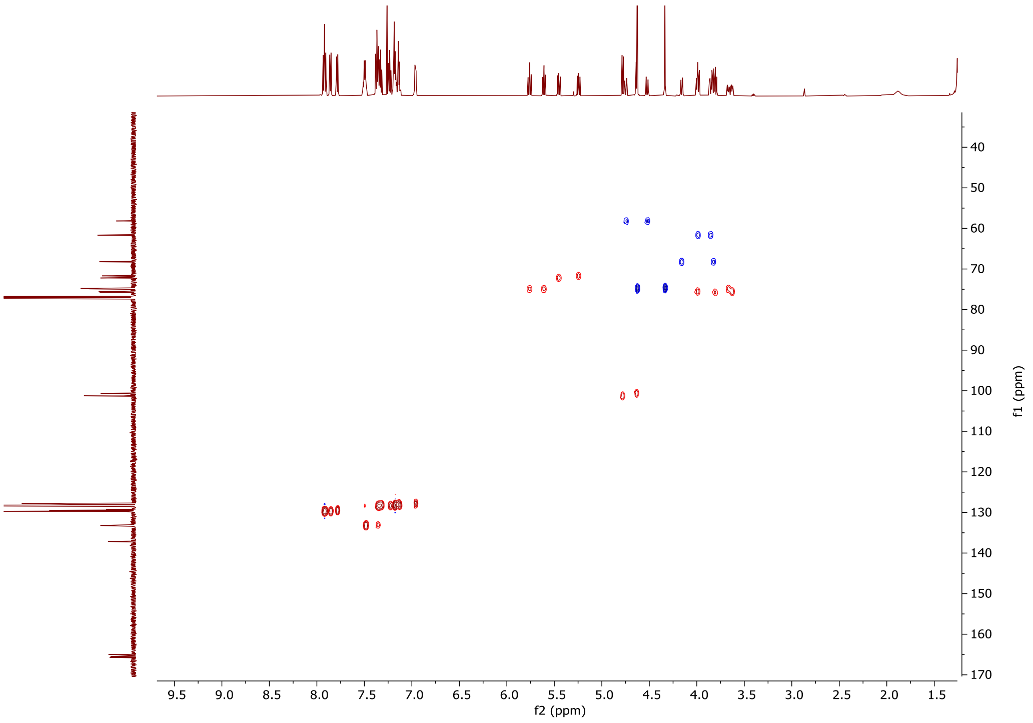

*Synthesis of **6***

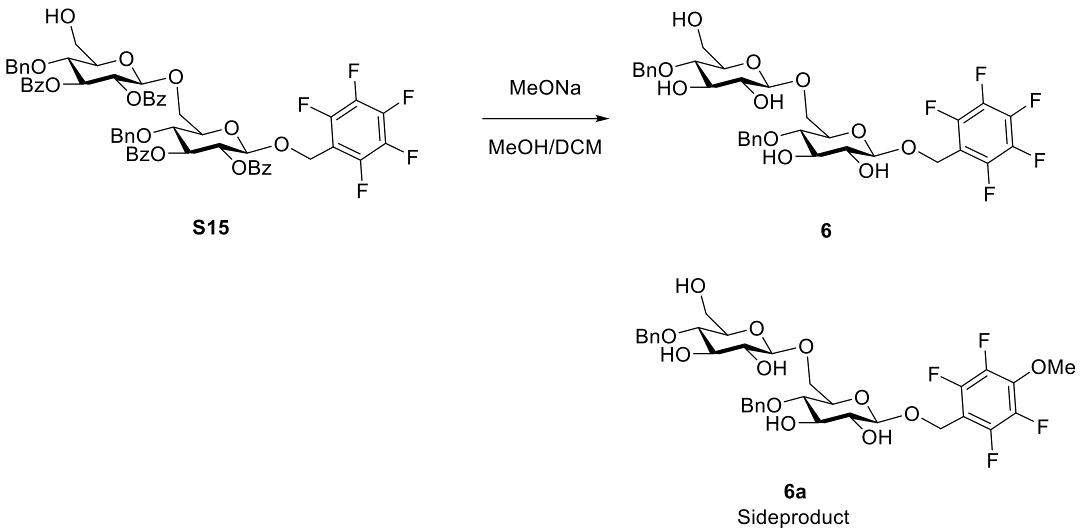

S15 (62 mg, 0.061 mmol) was dissolved in a 1:1 mixture of MeOH:DCM (3 mL). MeONa in MeOH (0.5 M, 3 equiv. per benzoyl ester) was added and the solution was stirred at RT for 16 h, neutralized with Amberlite IR-120 (H^+ form) resin, filtered, and concentrated *in vacuo*. ^{19}F NMR and ESI-MS analysis of the crude revealed the presence of side product **6a** (suggested in the scheme above) lacking the *para* fluorine (^{19}F NMR (564 MHz, CD_3OD) δ -146.61 (dd, J = 20.8, 8.9 Hz, 2F), -160.76 (dd, J = 21.0, 8.9 Hz, 2F) and (ESI-HRMS) m/z 737.2225 $[M+Na]^+$ ($C_{34}H_{38}F_4O_{12}Na$ requires 737.2197)). This side product **6a** was inseparable from **6** by TLC or silica gel manual column chromatography. The resulting yellow oil was passed through a short plug of silica gel (DCM:MeOH = 15:1), precipitated from DCM:Hexane and further purified with NP-HPLC (Method: YMC Diol, isocratic 30% EtOAc (5 min), linear gradient to 100% EtOAc (30 min), isocratic 100% EtOAc (5min)) to yield **6** as a colorless solid (9 mg, 21% yield). ^{1}H NMR (400 MHz, $CDCl_3$) δ 7.39 – 7.27 (m, 10H), 4.91 (dd, J = 11.6, 9.1 Hz, 2H), 4.85 (d, J = 11.3 Hz, 1H), 4.75 (d, J = 11.7 Hz, 1H), 4.69 (dd, J = 11.4, 5.2 Hz, 2H), 4.34 (d, J = 7.7 Hz, 1H), 4.29 (d, J = 7.8 Hz, 1H), 4.06 (d, J = 11.7 Hz, 1H), 3.86 (dd, J = 12.0, 2.6 Hz, 1H), 3.80 – 3.73 (m, 1H), 3.73 – 3.64 (m, 3H), 3.52 (d, J = 5.0 Hz, 2H), 3.48 – 3.34 (m, 3H), 3.34 – 3.27 (m, 1H).^{13}C NMR (101 MHz, $CDCl_3$) δ 138.16, 128.73, 128.71, 128.24, 128.19, 128.11, 103.38, 102.10, 77.27, 76.50, 75.52, 75.08, 74.84, 74.74, 73.71, 73.66, 68.27, 62.14, 58.27.^{19}F NMR (376 MHz, $CDCl_3$) δ -142.51 (dd, J = 22.5, 8.6 Hz, 2F), -152.60 (t, J = 20.8 Hz, 1F), -161.38 (td, J = 22.1, 8.5 Hz, 2F). (ESI-HRMS) m/z 725.2022 $[M+Na]^+$ ($C_{33}H_{35}F_5O_{11}Na$ requires 725.1992).

NP HPLC of crude reaction mixture (ELSD trace, t_R (6) = 22.4 min 6, t_R (6a) = 23.3 min)

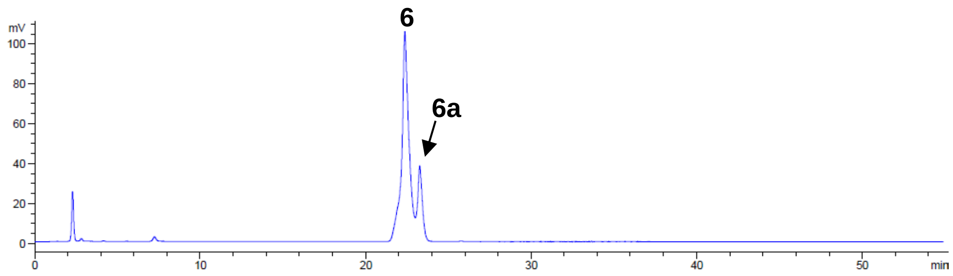

^{1}H NMR of 6 (400 MHz, $CDCl_3$)

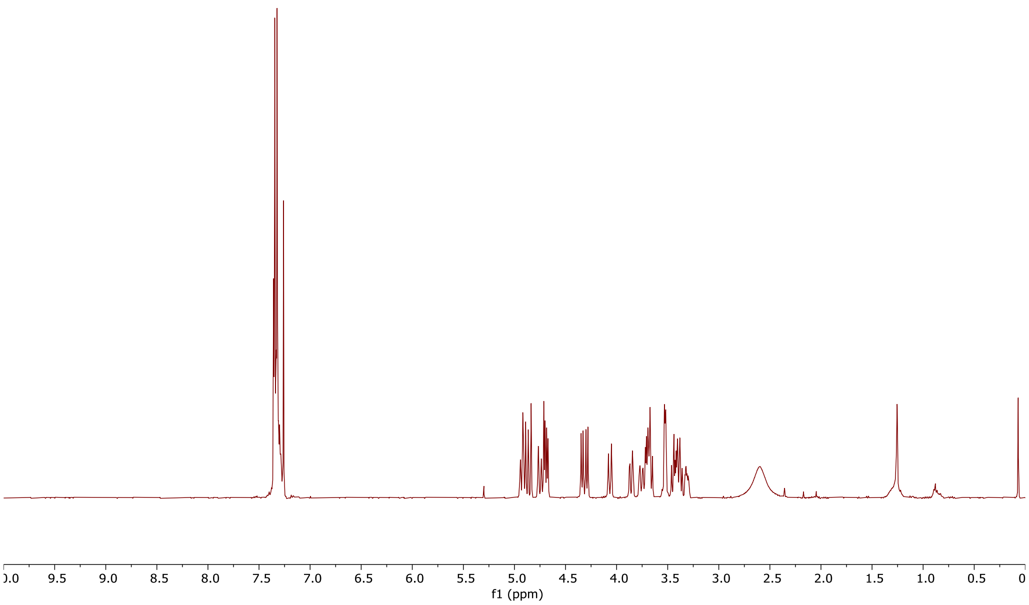

^{13}C NMR of 6 (101 MHz, $CDCl_3$)

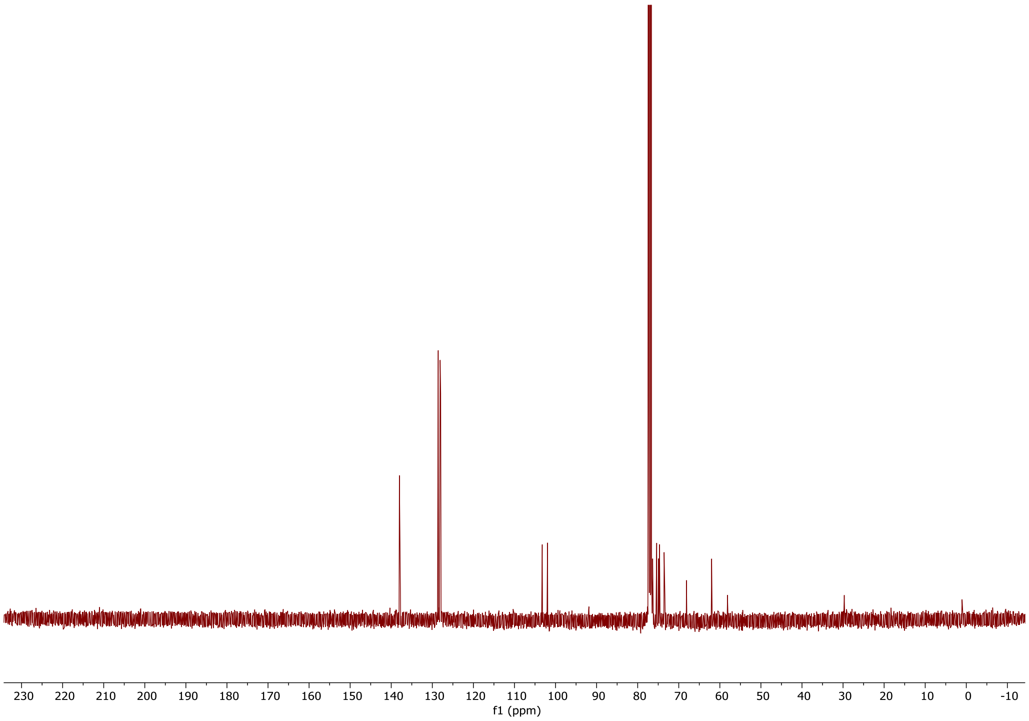

^{19}F NMR of 6 (376 MHz, $CDCl_3$)

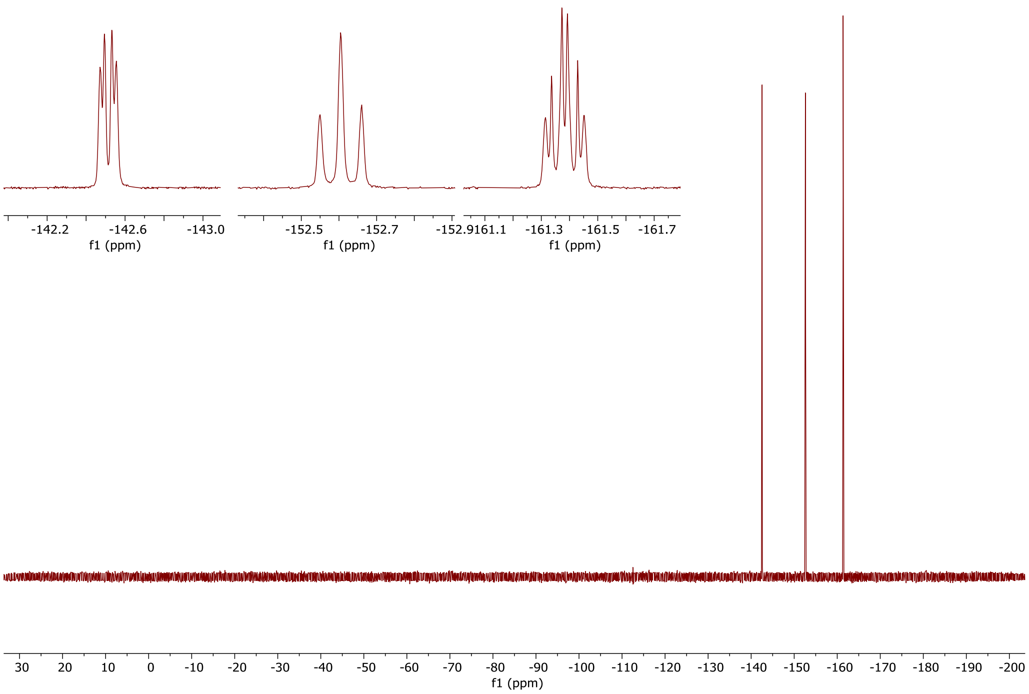

HSQC NMR of 6 ($CDCl_3$)

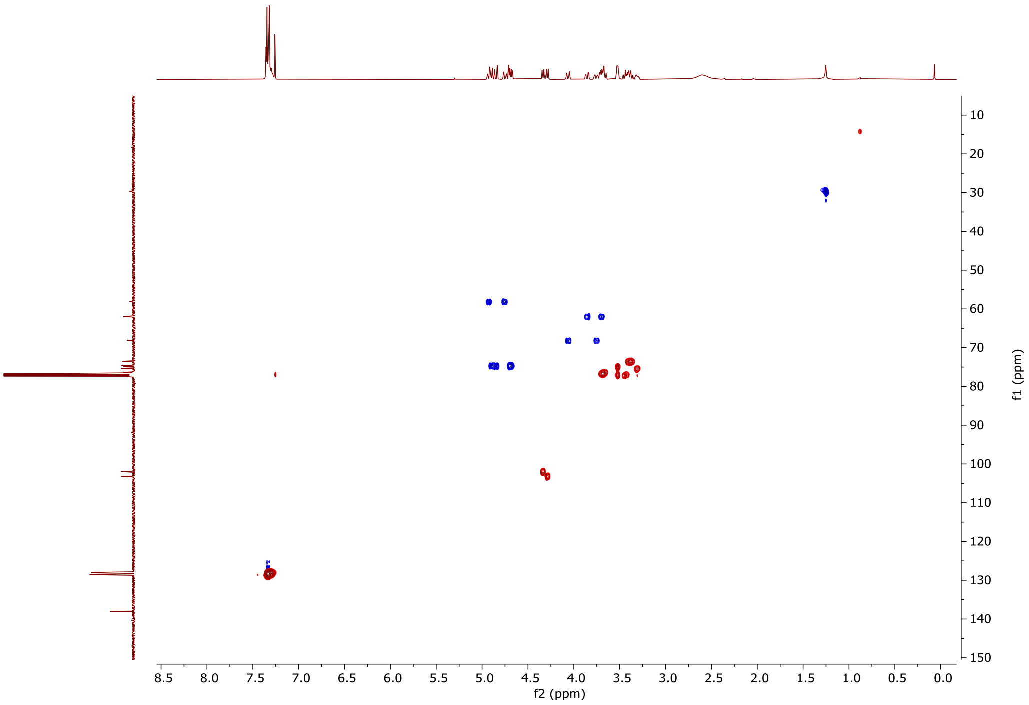

2.6. Synthesis of 7

Synthesis of ***S17***

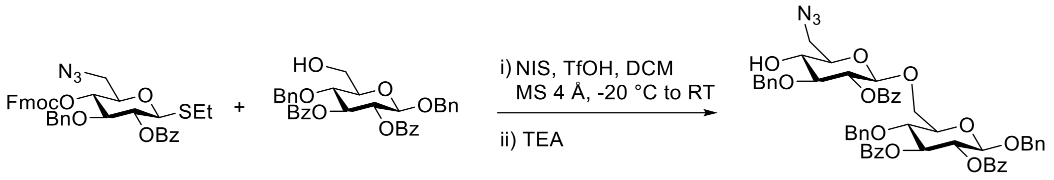

S16 **S2** **S17**

S16 was prepared according to previously established procedures.[6]

S16 (96 mg, 0.14 mmol), **S2** (82 mg, 0.14 mmol) and *N*-iodosuccinimide (39 mg, 0.17 mmol) were dissolved in anhydrous DCM (2 mL), then 4 Å molecular sieves were added. The system was charged with N_2, stirred for 30 min and cooled to -20 °C. TfOH (1.2 µL in 0.1 mL DCM, 0.014 mmol) was slowly added, and the reaction was warmed to 0 °C. After 1 h, triethylamine (1 mL) was added and the reaction was allowed to RT and stirred overnight. The reaction was diluted with DCM (10 mL) and washed with citric acid (0.5 M in water, 10 mL) twice and the water layer was extracted with additional DCM (10 mL). The organic layers were combined and dried over Na_2SO_4, filtered, and evaporated. The resulting crude product was purified by column chromatography (Hexane:EtOAc = 6:1→1:2) to give the **S17** as a white solid (104 mg, 78% over two steps). ^{1}H NMR (400 MHz, $CDCl_3$) δ 8.09 (d, *J* = 7.7 Hz, 2H), 7.94 – 7.84 (m, 4H), 7.58 – 7.46 (m, 3H), 7.45 – 7.33 (m, 7H), 7.32 – 7.08 (m, 12H), 6.99 – 6.93 (m, 2H), 5.60 (t, *J* = 9.5 Hz, 1H), 5.51 – 5.34 (m, 2H), 4.79 (d, *J* = 11.4 Hz, 1H), 4.74 (d, *J* = 7.9 Hz, 1H), 4.70 – 4.55 (m, 3H), 4.46 (d, *J* = 12.8 Hz, 1H), 4.39 (d, *J* = 10.9 Hz, 1H), 4.36 – 4.24 (m, 2H), 3.87 – 3.76 (m, 2H), 3.72 (dd, *J* = 7.2, 2.6 Hz, 2H), 3.68 – 3.57 (m, 2H), 3.55 (t, *J* = 4.3 Hz, 2H), 2.48 (s, 1H); ^{13}C NMR (101 MHz, $CDCl_3$) δ 165.61, 165.30, 165.05, 137.64, 137.17, 136.90, 133.39, 133.15, 133.09, 129.90, 129.78, 129.57, 129.43, 129.37, 128.70, 128.61, 128.34, 128.28, 128.21, 128.10, 127.85, 127.81, 127.72, 101.18, 99.00, 82.48, 76.22, 75.45, 75.08, 74.67, 74.64, 74.49, 73.63, 71.93, 70.78, 70.00, 68.10, 51.52; m/z (HRMS$^+$) [M + Na]$^+$ 972.3355 ($C_{54}H_{51}N_3O_{13}Na^+$ requires 972.3314).

^{1}H NMR of S17 (400 MHz, $CDCl_3$)

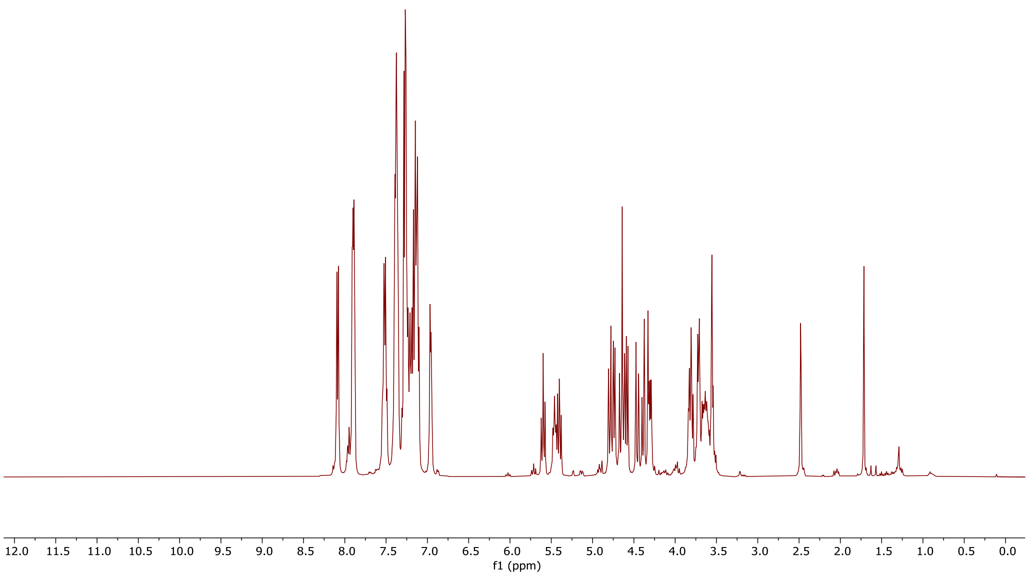

^{13}C NMR of S17 (101 MHz, $CDCl_3$)

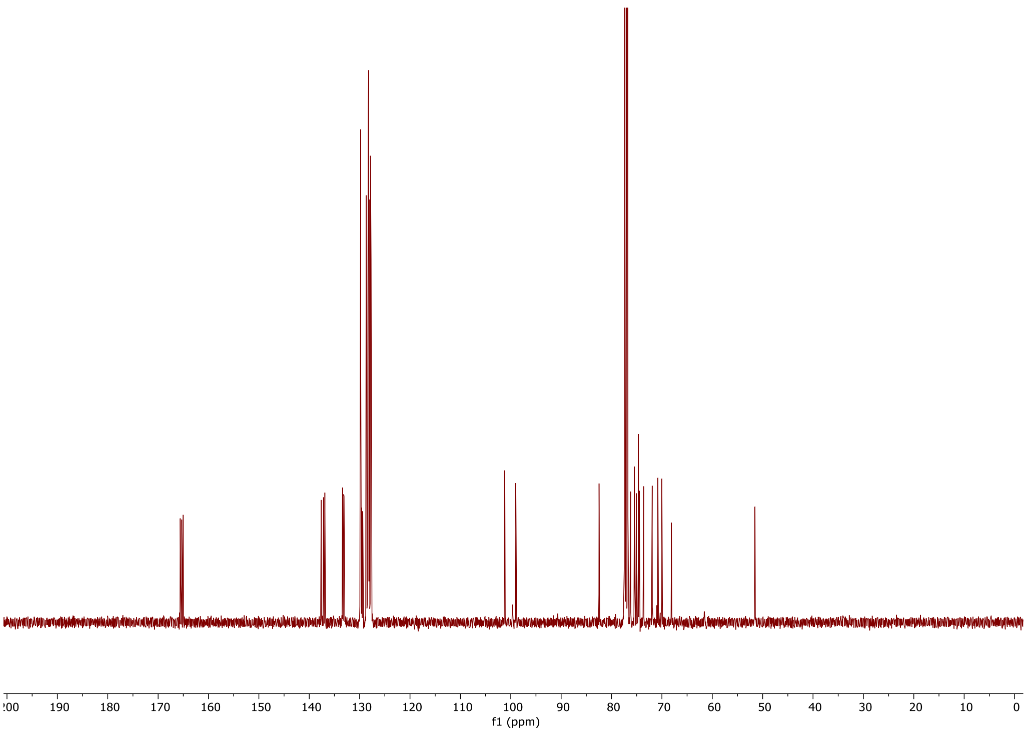

HSQC NMR of S17 ($CDCl_3$)

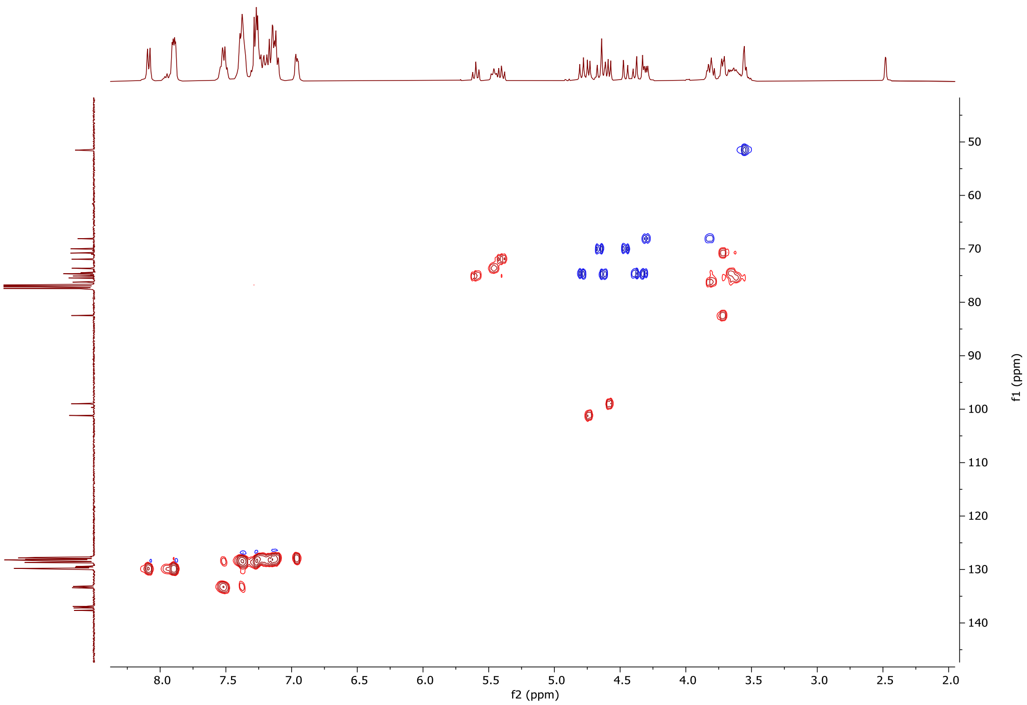

*Synthesis of **7***

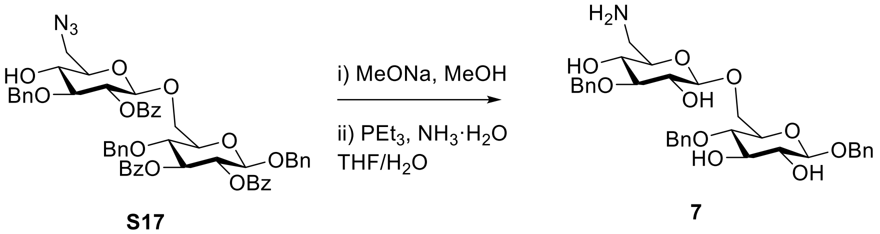

S17 (104 mg, 0.11 mmol) was dissolved in MeOH (1 mL), NaOMe in MeOH (0.5 M, 3 equiv. per benzoyl ester) was added, and the solution stirred at RT overnight. The reaction was neutralized with Amberlite IR-120 (H^+ form), filtered, and concentrated under reduced pressure. The obtained compound was dissolved in THF (1 mL), a triethylphosphine solution (1.0 M in THF, 0.5 mL) and an ammonia solution (30% in water, 0.2 mL) were added and the mixture was stirred overnight at RT. After evaporation, the resulting crude mixture was purified by column chromatography (DCM:MeOH = 15:1→3:1) to give **7** as a white solid (52 mg, 77% over two steps). ^{1}H NMR (400 MHz, CD_3OD) δ 7.46 – 7.41 (m, 6H), 7.38 – 7.24 (m, 9H), 5.05 – 4.85 (d×4, 4H), 4.74 (d, *J* = 11.1 Hz, 1H), 4.70 (d, *J* = 11.9 Hz, 1H), 4.46 (d, *J* = 7.7 Hz, 1H), 4.41 (d, *J* = 7.9 Hz, 1H), 4.22 (dd, *J* = 11.5, 1.1 Hz, 1H), 3.89 – 3.80 (m, 1H), 3.66 – 3.53 (m, 3H), 3.48 (dtd, *J* = 7.8, 4.9, 2.8 Hz, 2H), 3.41 – 3.29 (m, 4H), 3.07 (dd, *J* = 13.1, 8.4 Hz, 1H); ^{13}C NMR (101 MHz, CD_3OD) δ 138.85, 138.74, 137.64, 127.95, 127.94, 127.79, 127.74, 127.67(×2), 127.37, 127.29, 127.16, 103.92, 102.13, 84.05, 77.80, 77.07, 74.76, 74.71, 74.39, 73.97, 73.65, 71.93, 71.34, 70.66, 68.27, 40.52; m/z (HRMS$^+$) [M + H]$^+$ 612.2874 ($C_{33}H_{42}NO_{10}^+$ requires 612.2803).

^{1}H NMR of 7 (400 MHz, CD_3OD)

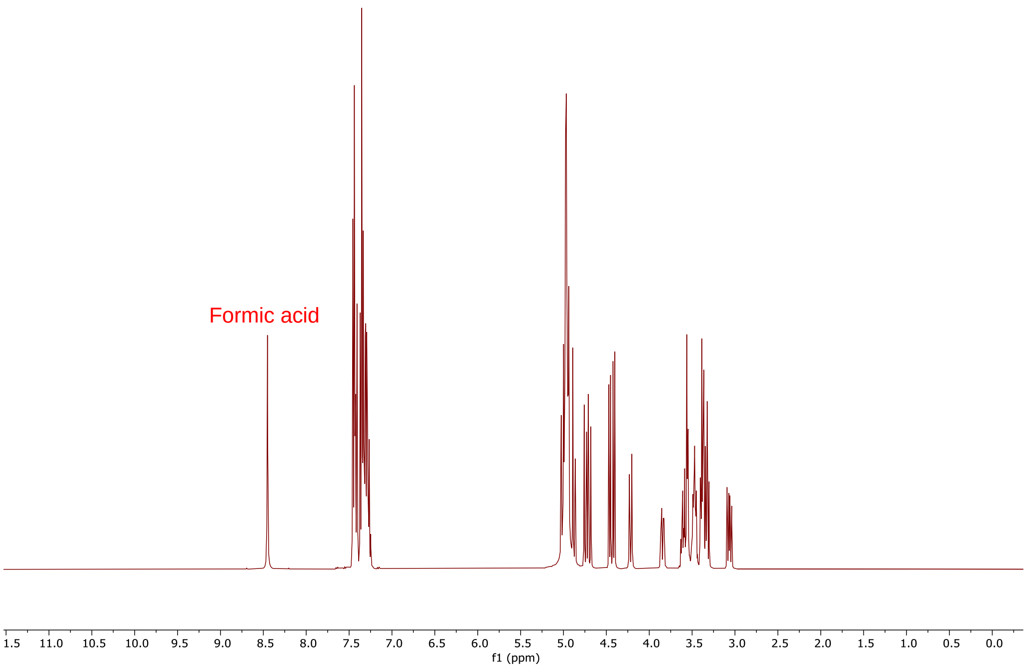

^{13}C NMR of 7 (101 MHz, CD_3OD)

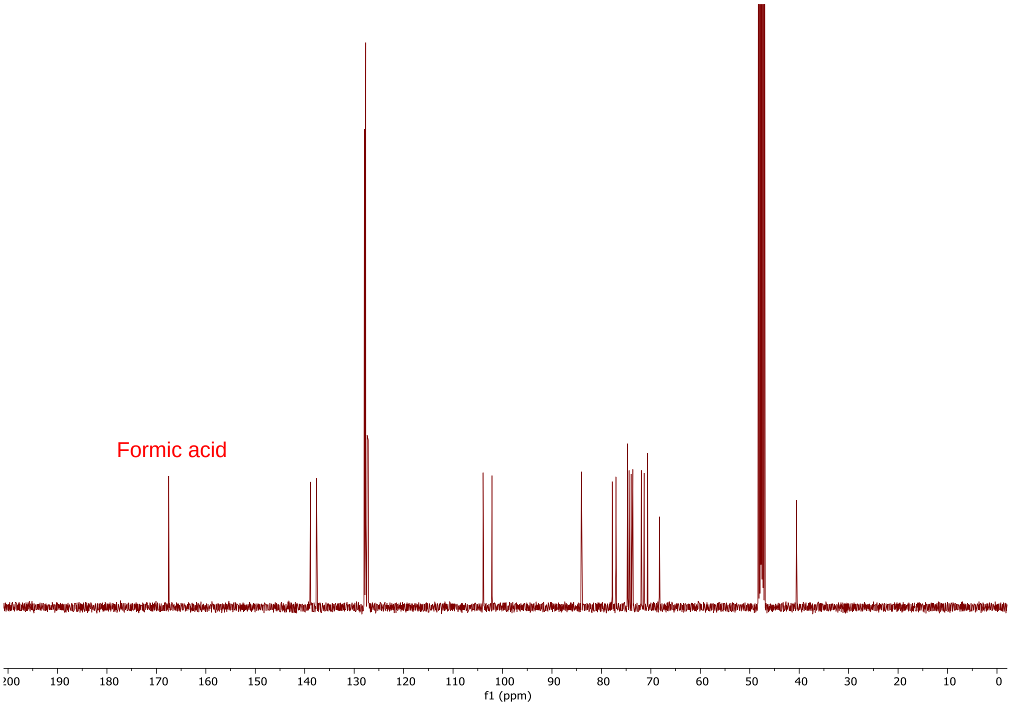

HSQC NMR of 7 (CD_3OD)

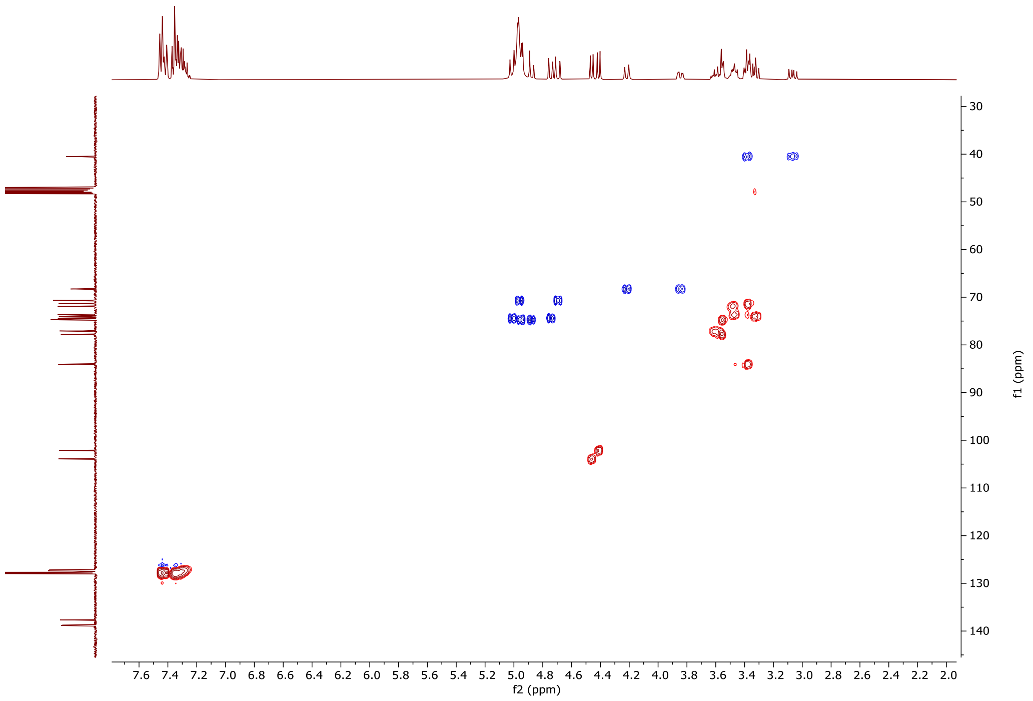

2.7. Synthesis of 8

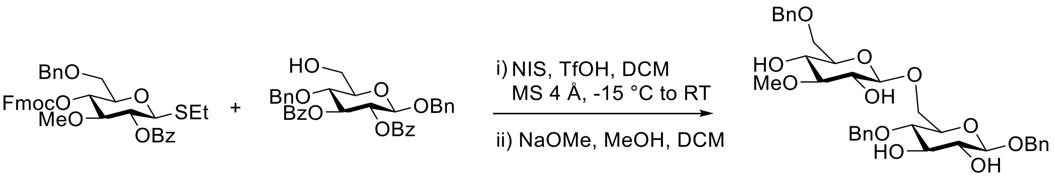

S18 **S2** **8**

S18 was prepared according to previously established procedures.[5]

S18 (40.3 mg, 0.062 mmol), **S2** (35.0 mg, 0.062 mmol), and *N*-iodosuccinimide (14.0 mg, 0.065 mmol) were dissolved in anhydrous DCM (5.0 mL). The solution was stirred with molecular sieve (4 Å) for 1 h at RT under nitrogen atmosphere and then cooled to -15 °C. A 1% solution of TfOH in DCM (20 µL) was added and the reaction was stirred for 30 min at -15 °C before the removal of the cooling bath to allow the reaction to RT. After TLC indicated the disappearance of **S18**, the reaction was diluted with DCM (10 mL) and washed with H_2O, then brine. The organic layer was dried over Na_2SO_4, filtered and evaporated. The resulting yellow oil was purified by column chromatography (hexane:EtOAc = 3:1) to give the fully protected disaccharide as white solid. The disaccharide was then dissolved in a 1:1 mixture of DCM:MeOH (4 mL). MeONa in MeOH (0.5 M, 3 equiv. per benzoyl ester) was added to the solution and the mixture was stirred at RT overnight, neutralized with Amberlite IR-120 (H^+ form) resin, filtered and concentrated *in vacuo*. The resulting yellow oil was purified by column chromatography (DCM:MeOH = 30:1) to give **8** as white solid (28.1 mg, 72%). ^{1}H NMR (400 MHz, CD_3OD) δ 7.41 – 7.19 (m, 15H), 4.91 (t, *J* = 11.1 Hz, 2H), 4.64 (dd, *J* = 11.5, 9.3 Hz, 2H), 4.52 (d, *J* = 2.1 Hz, 2H), 4.34 (d, *J* = 7.8 Hz, 1H), 4.29 (d, *J* = 7.9 Hz, 1H), 4.09 (dd, *J* = 11.5, 2.0 Hz, 1H), 3.80 – 3.67 (m, 2H), 3.57 (d, *J* = 22.2 Hz, 6H), 3.46 – 3.37 (m, 1H), 3.37 – 3.24 (m, 4H), 3.02 (t, *J* = 8.7 Hz, 1H); ^{13}C NMR (101 MHz, CD_3OD) δ 140.12, 139.72, 139.17, 129.46,

129.40, 129.38, 129.24, 129.22, 128.95, 128.76, 128.73, 105.05, 103.40, 87.81, 79.48, 78.53, 76.95, 76.10, 75.70, 75.42, 74.83, 74.54, 71.98, 71.33, 70.74, 69.81, 61.21; (ESI-HRMS) m/z 649.2522 $[M+Na]^+$ ($C_{34}H_{42}O_{11}Na$ requires 649.2619).

^{1}H NMR of 8 (400 MHz, CD_3OD)

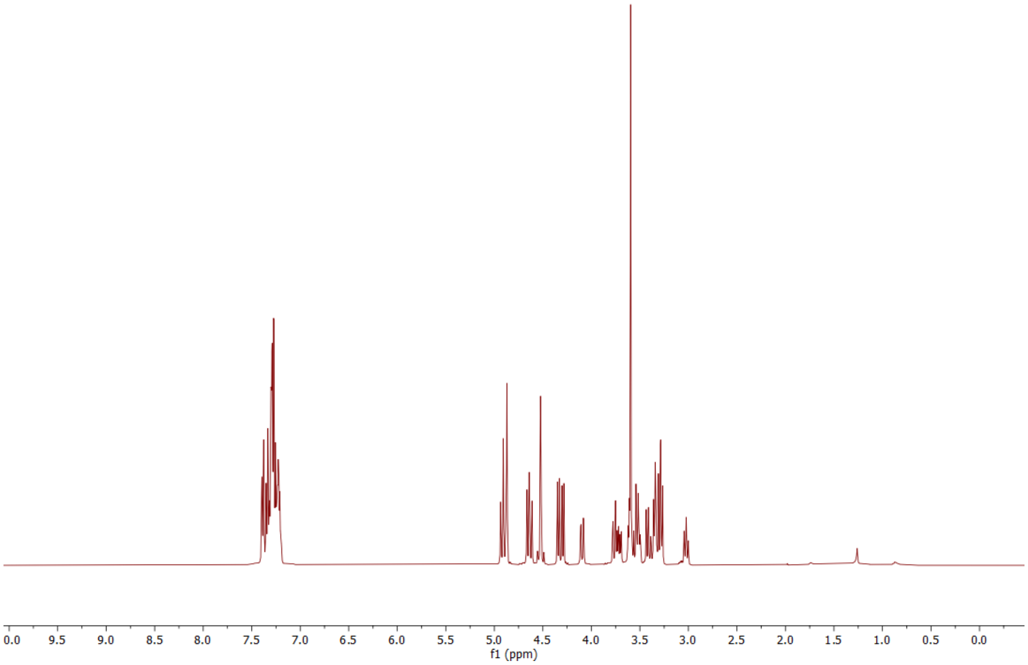

^{13}C NMR of 8 (101 MHz, CD_3OD)

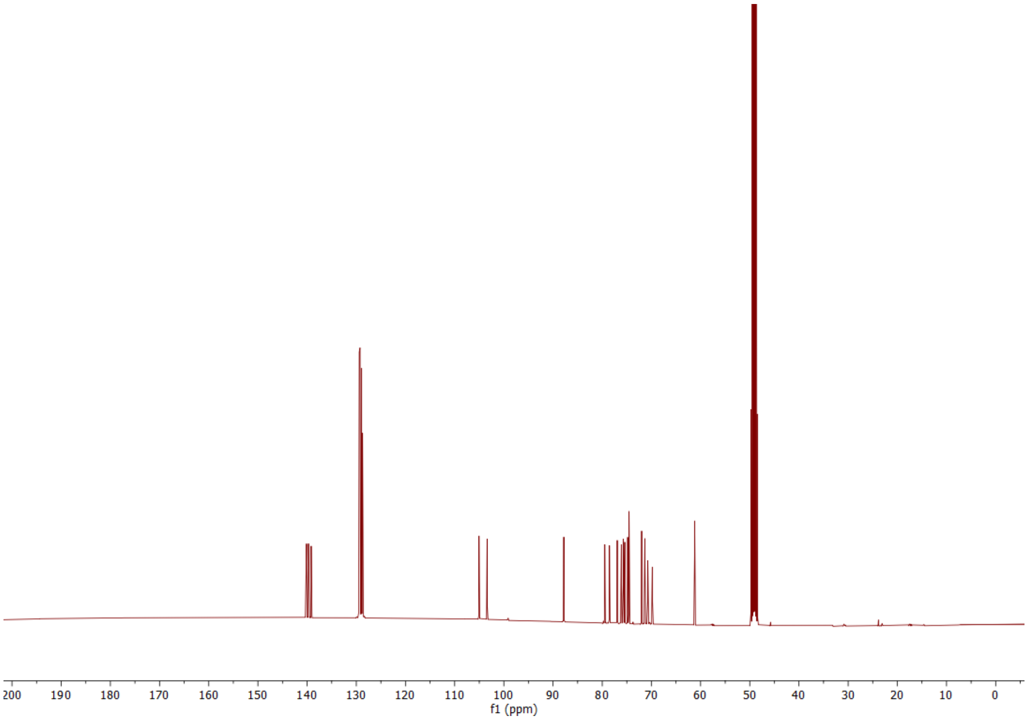

HSQC NMR of 8 (CD_3OD)

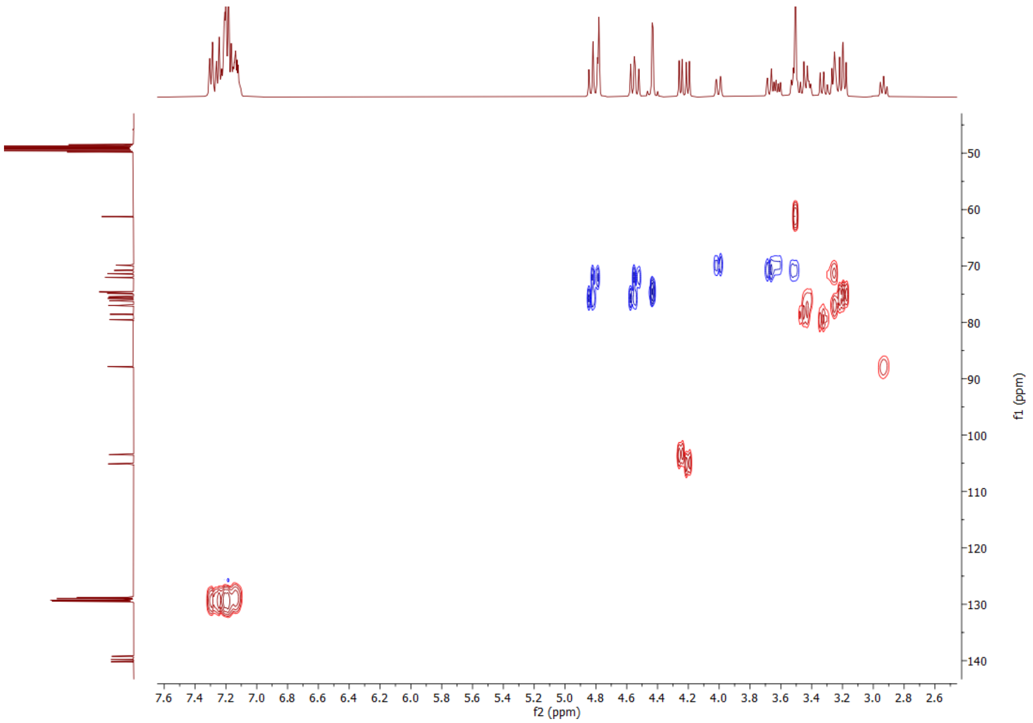

3. Assembly of carbohydrates

The sample preparation methods were previously established.[1, 3]

- **Solvent-switch method:** A stock solution of the oligosaccharide (100 mg mL^{-1}) in HFIP was diluted with milliQ water to reach the final concentration of 2 mg mL^{-1}**.** For the co-assembly of two compounds, two stock solutions were mixed in the desired mass ratio before addition of water. The final concentration of all self-assembled and co-assembled samples was 2 mg mL^{-1}.

- **Film-rehydration method:** 2 µL of the stock solution was drop casted on a glass microscope slide. After drying, the substrate was placed in a humidity chamber and the structure transition was observed with a polarized optical microscope at different time intervals.

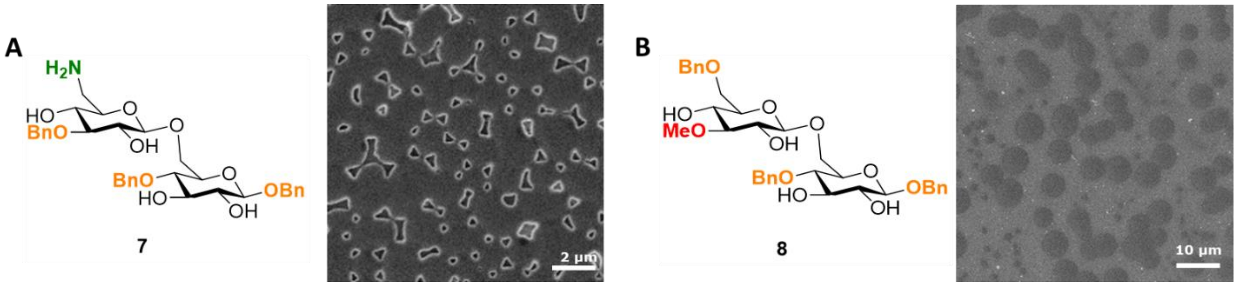

Figure S2 Chemical structures and assembled architectures for **7** (A) and **8** (B) prepared upon solvent-switch.

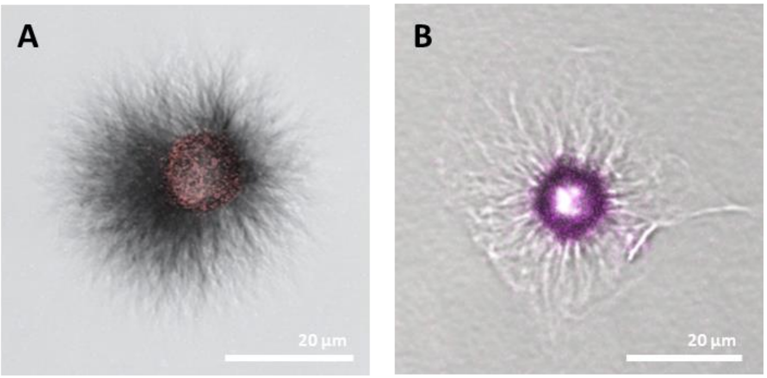

Figure S3 Confocal microscopy images of the fiber formation of **1** (A) and **3** (B) obtained upon switch method. The fibers are generated radially from a droplet of oligosaccharide in HFIP upon addition of water. The images were captured after 10 minutes adding water.

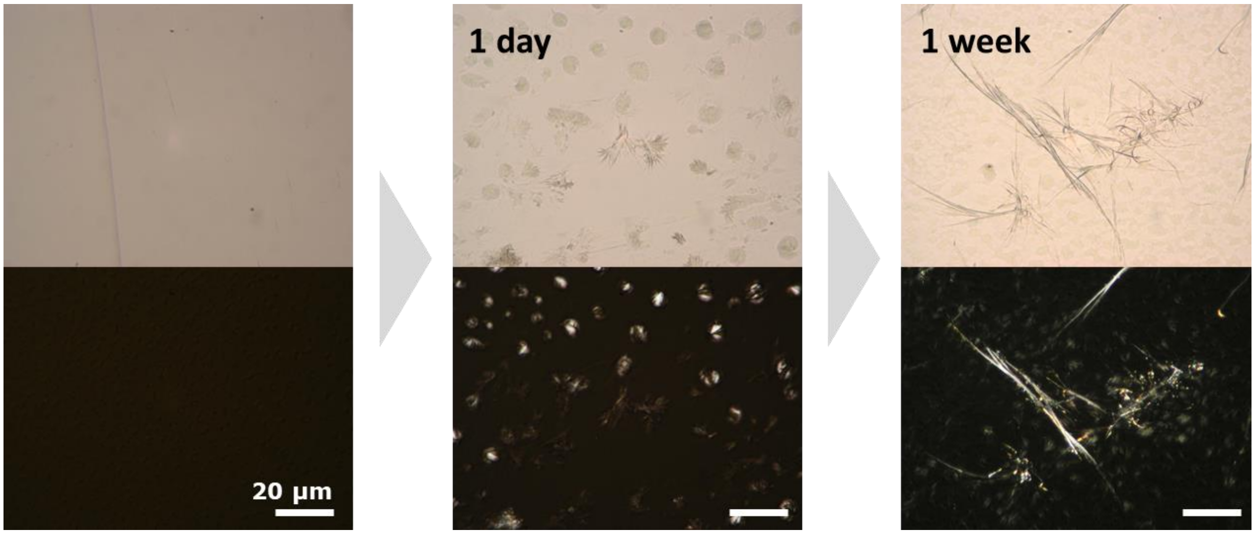

Figure S4 Polarized optical microscopy images of the fiber formation of **2** obtained upon film-rehydration. The assembly of **2** is much slower than for **1** and **3**, therefore it was not possible to observe the transition in real time. The film-rehydration method was chose as it was reported to generate a similar morphology for **1**.[1]

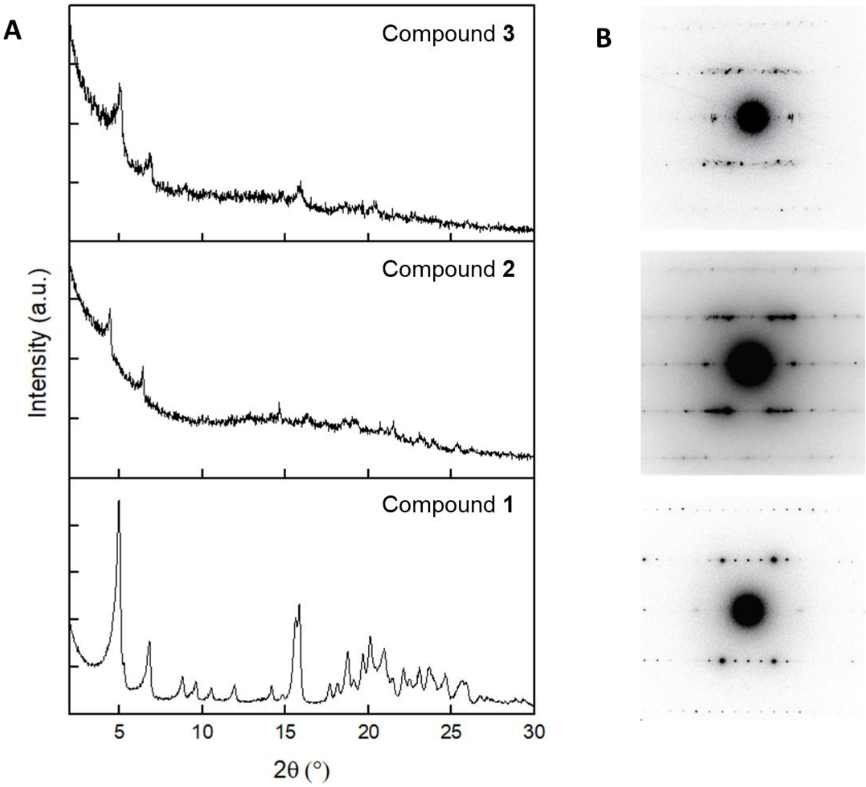

Figure S5 Powder X-ray (A) and microcrystal electron diffraction (B) patterns of **1** (bottom), **2** (middle), and **3** (top). The XRD for compounds **2** and **3** show relatively strong intensities in the lower Q, whereas the higher-angle reflections are weak. This observation corresponds to what observed in the electron diffraction diagram (B).

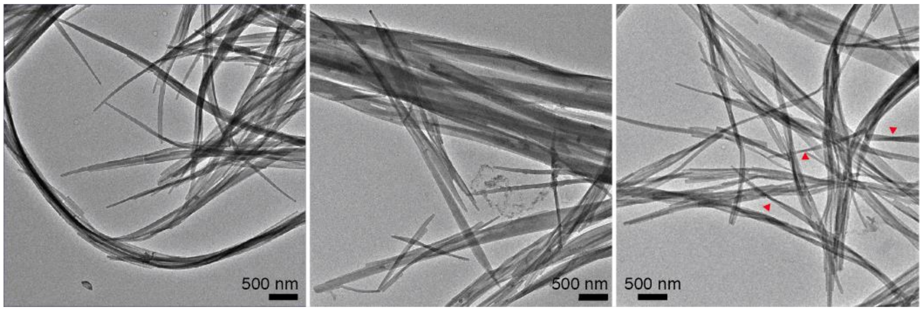

Figure S6 TEM image of the assembly of **3**. Twisted parts are highlighted with red arrowheads.

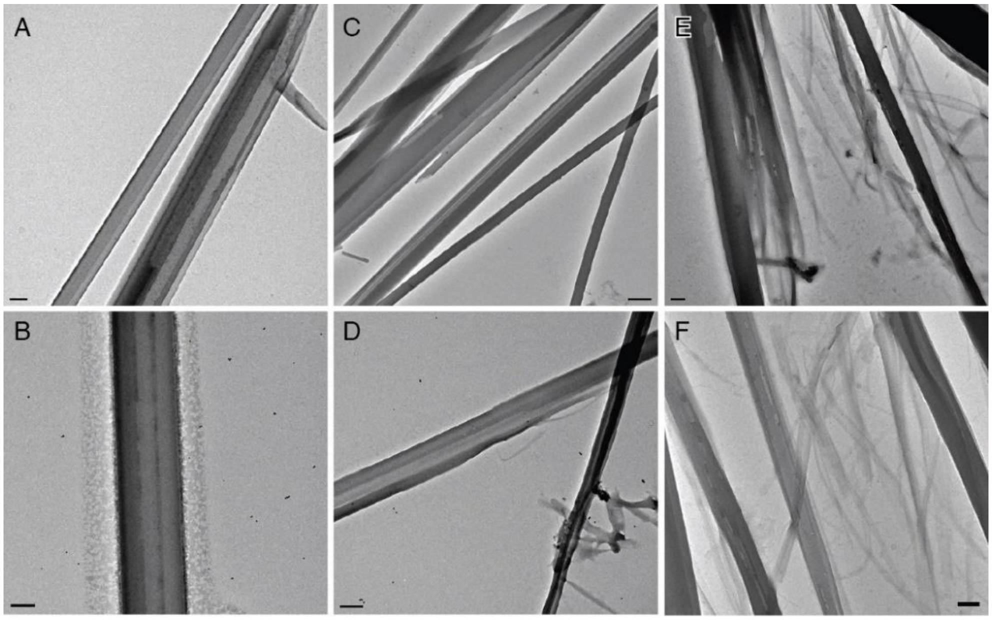

Figure S7 TEM images of the assembly of **2** at pH 3 (A, B), 7 (C, D), and 11 (E, F). (Scale bars: 500 nm)

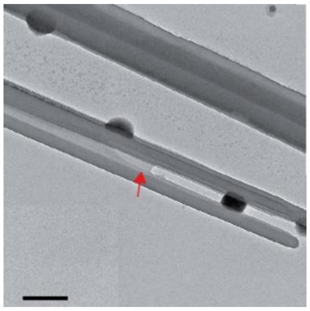

Figure S8 TEM image of the assembly of **2** at pH 3. Red arrow indicates thin membrane-like structure connecting two fibrous crystallites. (Scale bar: 500 nm)

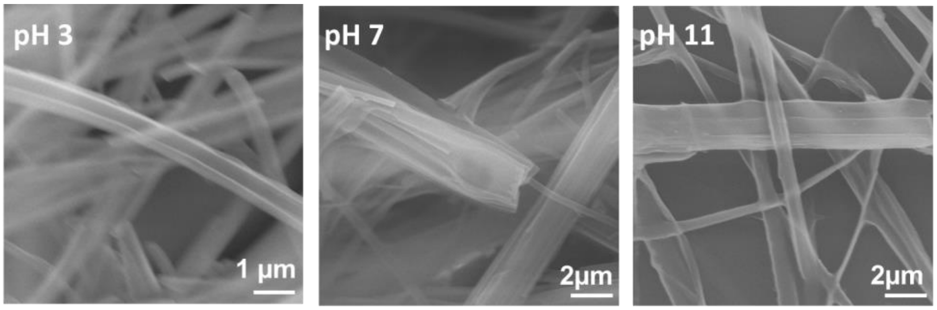

Figure S9 SEM images of the self-assembly of **2** at different pH.

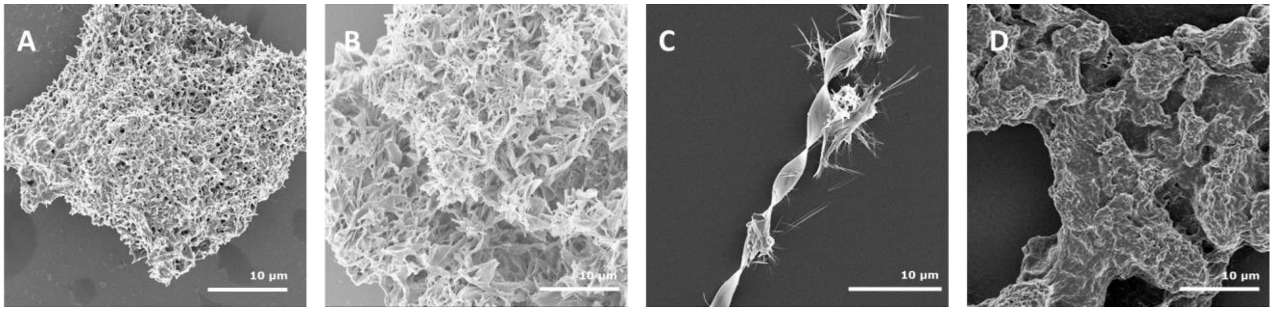

Figure S10 SEM images of the (1:1 mass ratio) co-assembly of **1** with **5** (A), **6** (B), **7** (C), and **8** (D).

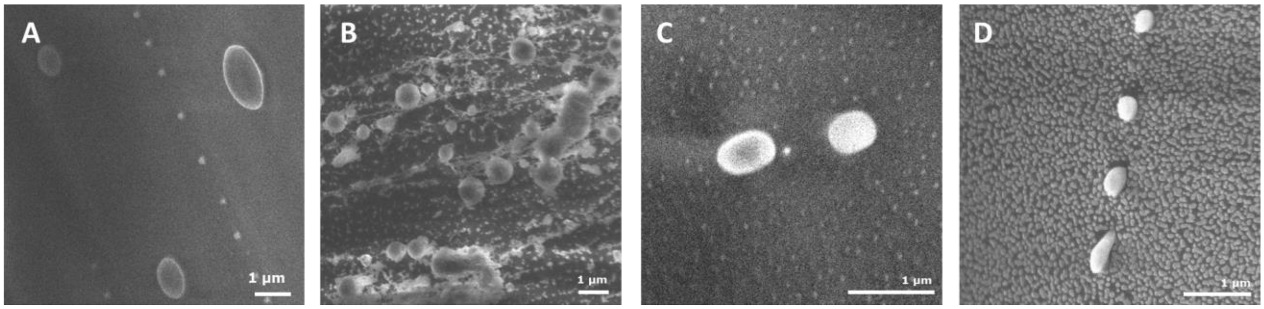

Figure S11 Cryo SEM images of the self-assembly of **4** (A) and the co-assembly of **1** and **4** with 1:1 (B), 1:2 (C), and 1:4 (D) mass ratio.

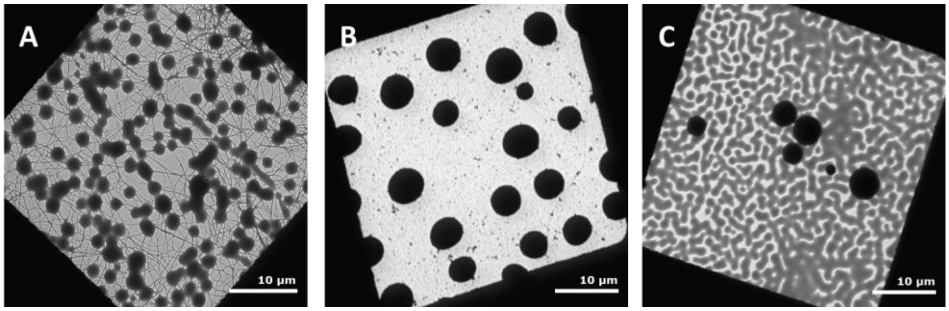

Figure S12 TEM images of the co-assembly of **1** and **4** with 1:1 (A), 1:2 (B), and 1:4 (C) mass ratio.

4. References

[1] S. Gim, G. Fittolani, Y. Nishiyama, P. H. Seeberger, Y. Ogawa, M. Delbianco, *Angew. Chem. Int. Ed.* **2020**, *132*, 22766-22772.

[2] D. N. Mastronarde, *Microsc. Microanal.* **2003**, *9*, 1182-1183.

[3] Y. Yu, S. Gim, D. Kim, Z. A. Arnon, E. Gazit, P. H. Seeberger, M. Delbianco, *J. Am. Chem. Soc.* **2019**, *141*, 4833-4838.

[4] M. Delbianco, A. Kononov, A. Poveda, Y. Yu, T. Diercks, J. Jiménez-Barbero, P. H. Seeberger, *J. Am. Chem. Soc.* **2018**, *140*, 5421-5426.

[5] Y. Yu, T. Tyrikos-Ergas, Y. Zhu, G. Fittolani, V. Bordoni, A. Singhal, R. J. Fair, A. Grafmüller, P. H. Seeberger, M. Delbianco, *Angew. Chem. Int. Ed.* **2019**, *58*, 1433-7851.

[6] Y. Zhu, T. Tyrikos-Ergas, K. Schiefelbein, A. Grafmüller, P. H. Seeberger, M. Delbianco, *Org. Biomol. Chem.* **2020**, *18*, 1349-1353.

[7] M. Delbianco, A. Kononov, A. Poveda, Y. Yu, T. Diercks, J. s. Jiménez-Barbero, P. H. Seeberger, *J. Am. Chem. Soc.* **2018**, *140*, 5421-5426.
